# Estimating the extent and sources of model uncertainty in political science

**DOI:** 10.1073/pnas.2414926122

**Published:** 2025-06-17

**Authors:** Michael Ganslmeier, Tim Vlandas

**Affiliations:** ^a^Centre for Computational Social Science, Department of Humanities, Arts and Social Sciences, University of Exeter, Exeter EX4 4PY, United Kingdom; ^b^Department of Social Policy and Intervention, University of Oxford, Oxford OX1 2ER, United Kingdom

**Keywords:** model uncertainty, sensitivity analysis, robustness checks, modeling choices, quantitative political science

## Abstract

Assessing model uncertainty is crucial to quantitative political science. Yet, most available sensitivity analyses focus only on a few modeling choices, most notably the covariate space, while neglecting to jointly consider several equally important modeling choices simultaneously. In this article, we combine the exhaustive and systematic method of the Extreme Bounds Analysis with the more multidimensional logic underpinning the multiverse approach to develop an approach to sensitivity analyses. This allows us to systematically assess the degree and sources of model uncertainty across multiple dimensions, including the control set, fixed effect structures, SE types, sample selection, and dependent variable operationalization. We then apply this method to four prominent topics in political science: democratization, institutional trust, public good provision, and welfare state generosity. Results from over 3.6 bn estimates reveal widespread model uncertainty, not just in terms of the statistical significance of the effects, but also their direction, with most independent variables yielding a substantive share of statistically significant positive and negative coefficients depending on model specification. We compare the strengths and weaknesses of three distinct approaches to estimating the relative importance of different model specification choices: nearest 1-neighbor; logistic; and deep learning. All three approaches reveal that the impact of the covariate space is relatively modest compared to the impact of sample selection and dependent variable operationalization. We conclude that model uncertainty stems more from sampling and measurement than conditioning and discuss the methodological implications for how to assess model uncertainty in the social sciences.

## Significance

In social sciences, assessing model uncertainty is essential. Yet traditional sensitivity analyses often focus on a few modeling choices, most notably the selection of control variables. This article develops a systematic approach to evaluate model uncertainty across various dimensions, including fixed effect structures, SE types, sample selection, and dependent variable operationalization. We apply our method to four political science topics: democratization, institutional trust, public good provision, and welfare state generosity. Our findings show that model uncertainty arises primarily from sampling and measurement rather than the choice of covariates, even when excluding problematic specifications and models with low predictive power. We develop an R library that can be used by social science researchers to implement these systematic sensitivity checks.

One of the fundamental methodological challenges in the quantitative social sciences concerns how to measure the nature and extent of model uncertainty. Empirical analyses inevitably require making assumptions and choices, which encompass a broad range of modeling choices, for instance about the data structure, sample selection, measurement, covariate set, and estimation strategy. Ideally, key results should exhibit minimal variability to equally reasonable modifications in methodological choices. However, even minor changes in the statistical assumptions and modeling choices can in practice significantly affect the stability of empirical findings. While certain methodological choices are relatively straightforward as they can be clearly and transparently motivated with reference to established methodological conventions and statistical theory, others remain subject to debate and are challenging to validate a priori. Ideally, applied researchers therefore require a method that can be used to evaluate how sensitive their results are to all possible combinations of equally justifiable choices in their empirical approach.

Several approaches have been developed to evaluate the robustness of empirical estimates to different methodological choices. Prominent examples include the Extreme Bounds Analysis (EBA) method (1, 2), the perturbation analysis (3), Bayesian statistics (4), the Bayesian Model Averaging (5) and the Weighted Average Least Squares (6) approaches. While these methods have been successfully used in numerous studies to carry out sensitivity analyses both in economics and political science (7–9), they focus on the assessment of robustness regarding the covariate set. This is undeniably one of the key choices faced by applied researchers, but not considering robustness to other modeling choices risks underestimating the actual degree of model uncertainty. Indeed, more recent methodological contributions have demonstrated the importance of other empirical choices, for instance in terms of outliers, the structure and processing of the chosen dataset, and the construction of the null hypothesis (10–15). In addition, the multiverse approach has considered more dimensions across data and models (13, 16–20). Thus, there has been much progress in considering sensitivity to a wide range of empirical choices. However, one key challenge is how to ensure that all relevant robustness checks are fully and transparently explored, while at the same time excluding robustness checks that cannot be justified on theoretical or statistical grounds. Running all possible models risks introducing errors in the empirical approach, but selecting a subset of robustness checks risks reintroducing the “researcher’s degree of freedom” that sensitivity analyses were trying to address in the first place (cf. 14).

In this article, we apply an approach to systematically quantify the level and source of model uncertainty across multiple model specification choices. Specifically, our sensitivity analysis assesses the impact of all possible combinations of five crucially important model assumptions and specification choices, including the fixed effect structure, the SE, the country as well as period sample, and the operationalization of the dependent variable. We apply this method to four prominent topics in political science concerning welfare state generosity, democratization, regional public good provision, and individual trust in institutions, respectively. Running all possible combinations of these model assumptions and specification choices yields approximately 3.6 billion estimated coefficient–SE pairs. We analyze these in two steps: first, by examining how sensitive the statistical significance and direction of an effect are to these choices; and second, by identifying the factors that drive this sensitivity, assessing how different empirical choices influence the stability of the results.

Our analysis yields two key findings. First, most determinants under scrutiny are highly sensitive to even minor modifications in the underlying empirical assumptions and modeling choices. The resulting model uncertainty is substantially larger than previously assumed in terms of the varying share of significance and the direction of the coefficient. Indeed, we show that the coefficient space encompasses many statistically significant estimates that point in both positive and negative directions. This bidirectionality is especially concerning for factors which can be theorized in opposing directions. These results remain when imposing restrictions by excluding problematic (i.e., due to violation of statistical assumptions) and low prediction models.

Next, we compare the strengths and weaknesses of three potential approaches to evaluating the relative importance of different modeling choices. First, we introduce a method, the “1-nearest-neighbor method,” to measure the average share of cases where a coefficient change leads to a shift in significance class when a single modeling choice is randomly altered. This approach is intuitive, easy to implement, and computationally efficient, making it a valuable tool for capturing broad patterns of model sensitivity. Specifically, it identifies how often a coefficient’s significance class changes in response to a single modification in the specification vector on which it is estimated. Second, we complement this approach with a multinomial logistic model which predicts the probability that a result falls into a particular significance category (“not significant,” “positive significant,” or “negative significant”) based on the modeling choices. Notably, this approach provides a continuous probability scale from 0 to 1, allowing us to detect more subtle shifts in predicted significance which makes it particularly useful for capturing marginal effects, even in cases where the significance class itself does not change. While this second approach improves sensitivity to small shifts in the predicted probability, it still imposes assumptions on the functional form capturing the relationship between inputs and outcomes. To address this limitation, we adopt a third approach: a deep learning model using neural networks. This method provides a highly flexible predictive framework, capable of capturing complex interactions and nonlinear relationships. However, this flexibility comes at the cost of interpretability and ease of implementation. Although all three approaches consistently indicate that control variable selection has a relatively minor impact on statistical significance compared to the measurement of the outcome variable and sample selection, they have distinct strengths and offer different perspectives on the relative importance of key modeling choices.

Overall, our findings emphasize that model uncertainty emanates primarily from sampling and measurement rather than conditioning. Although we have purposefully selected four datasets that have very different structures and sample sizes, we do not wish to claim that the sensitivity we find in these topics is necessarily representative of other topics in political and social sciences. Instead, our article provides evidence and a proof of concept of the benefits of using our methodological approach to assess empirical sensitivity, so that other researchers can then apply this method to other cases. Indeed, given our conclusion about the importance of expanding the range of robustness checks, we provide an R library that is available to other scholars in their respective fields. To make our approach tractable in a more time and computing efficient manner, we show that the model space increases exponentially with the covariate space so focusing on a slightly smaller control set reduces the time it takes to perform these robustness checks. In addition, we demonstrate that researchers wishing to assess a large covariate space would only need to carry out a random sample of all possible permutations to arrive at a fairly accurate approximation of sensitivity of the full model space. Whether or not carrying out the full model space is worth the additional temporal costs depends on the number of modeling choices being tested and the importance of accuracy in sensitivity: The lower the former, the faster the full model space can be implemented; but as the number of modeling dimension increases, a random sample approach may be more cost effective, as our simulation tests have shown (*SI Appendix*).

In the next section, we present our main results concerning the extent, nature, and sources of model uncertainty. Next, we discuss their wider implications for robustness checks and sensitivity analyses in the political sciences and beyond. Finally, we describe our cases, data and methodological approach in a separate *Material and Methods* section.

## Results

In this section, we present the results from our sensitivity analysis applied to four prominent topics in political science: welfare state generosity, democratization, regional public good provision, and individual trust in institutions. For each topic, drawing inspiration from the EBA approach developed by Leamer (1, 2), we systematically vary all possible combinations of our key modeling choices (for more background on this approach, see *SI Appendix*, section S0.3). We go beyond EBA’s focus on the control set by including a wider range of empirical specification choices, building on the insights of the multiverse literature (13, 16–20). Specifically, we assess the extent to which the statistical significance and direction of an estimate for a given independent variable varies when changing the accompanying control set as well as the unit and period samples, the operationalization of the dependent variable, the fixed effect structure, and/or the type of SE (see the *Material and Methods* section at the end of this article for more information). Presenting a comprehensive literature review and discussion of variable choice in the main text for each topic is not possible given the word count limit. Instead, this information can be found in *SI Appendix*, sections S1.1, S2.1, S3.1, and S4.1. Nevertheless, we first illustrate and test the approach in more detail for the case of the welfare state and then extend it to other cases in a second step.

### The Extent of Model Uncertainty: The Case of Welfare State Development.

To test our method on the case of welfare state development, we construct a panel dataset consisting of country-year observations covering 33 countries between 1960 and 2016. Based on a literature review (*SI Appendix*, section S1.1), we select 18 independent variables capturing relevant economic, political, and institutional factors that have been previously found to be associated with welfare state development. In addition, we specify five additional sets of model specifications. First, we vary the sample restrictions by welfare state regime classification (21). Second, we split each of these country datasets along several time periods: 1980-2000, 2000-2016, and 1980-2016. Third, we run the analysis for six different measures of welfare state generosity. Finally, fourth and fifth, we apply different fixed effect structures and SE types. Overall, with 18 independent variables, we create a control set for each combination of variables, resulting in a total of 262,143 control sets. Each of these sets is used in combination with seven country samples, three period samples, six dependent variables, four fixed effect structures, and three SE types. Thus, in total, with 262,143 control sets and 1,512 model specifications (7 country samples × 3 period samples × 6 dependent variables × 4 fixed effect structures × 3 SE types), we end up with 396,360,216 regressions in the entire model space.

**Fig. 1.**
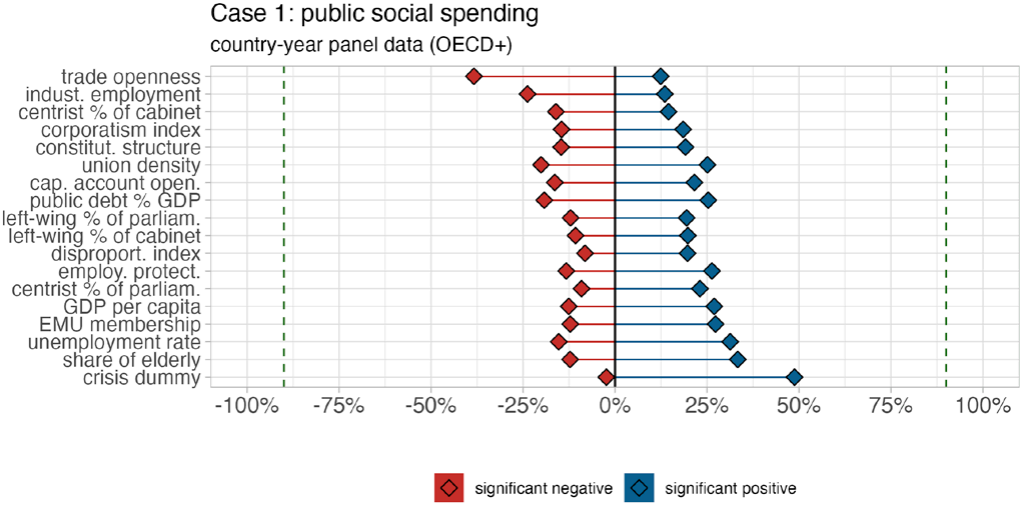
Significance shares of the independent variables. The figure plots the share of positive (blue) and negative (red) significant coefficients of all independent variables in the full model space. A coefficient is classified as “significant” if its *P*-value is below 0.1. The dashed line indicates 90%.

Our results underscore the prevalence of model uncertainty with low levels of robustness in terms of the statistical significance for all independent variables (Fig. 1). Specifically, the vast majority of independent variables exhibit coefficients that are significant in between 20% and 40% of all estimates. In addition, the results also indicate a high share of coefficients switching signs, i.e., a bidirectional sensitivity in the model space, for almost all variables. In other words, almost all independent variables reveal a large number of estimates that point in either positive or negative direction. Our approach also makes it possible to explore how the significance shares of the independent variables vary by different SE types, fixed effect structures, dependent variables, country samples, and period samples (*SI Appendix*, Figs. S1.2.2–S1.2.6), thereby allowing researchers to identify which modeling choices are particularly consequential for which independent variable.

One potential shortcoming of the multiverse approach (17, 22), which might also be problematic in our unrestricted model space, is that there might be a large fraction of misspecified or statistically problematic models. If this is the case, the unrestricted model space can skew the results in both directions and thus bias the conclusions. As we describe in *Material and Methods* section, we reproduce our analysis while applying three types of restrictions/weighting schemes to the model universe. First, we exclude all regression models that suffer from multicollinearity by dropping all regressions in which the independent variable of interest has a Pearson correlation coefficient (absolute size) that is larger than 0.5 with one (or more) covariates. Second, we shrink the model universe to estimations based on a goodness of fit measure to the top 10% best fitted models. The significance shares of these two restricted model universes are very similar to the findings we derived from the unrestricted model universe (*SI Appendix*, Figs. S1.3.1 and S1.3.2). Third, we weigh individual estimates with their model’s respective likelihood ratio index: Our analysis reveals that all independent variables exhibit probability distributions with masses substantially below the 90% threshold on both sides of zero (*SI Appendix*, Figs. S1.3.3 and S1.3.4).

One potential constraint of our method is computational. Indeed, running 396,360,216 regressions to create and extract the 3.57 billion estimates in the entire model space is computationally expensive. As this will not be practical for many applications in most cases, we provide the demonstration in *Material and Methods* section that selecting and running a much smaller random sample of models from the entire model space approximates the distribution across the entire model universe (*SI Appendix*, Fig. S1.5.1). Thus, our approach shows that one does not have to run the full universe of modeling choices, but instead that one can adopt a random sample on this full universe while considering many more modeling choices than is currently the case and applying plausible restrictions to the model space. In the next section, we consider three other topics, with very different data structures, to explore the external validity of our findings beyond the case of the welfare state.

### The Extent of Model Uncertainty in Other Cases.

While we have documented a large degree of sensitivity in the statistical significance and direction of estimates for our main case, it could be that this was driven by the panel data structure typical of research on this topic, the fact that the selected variables were chosen on the basis of a literature review which we carried out, the region and time period coverage, or the sample size. Thus, we replicate our analysis for three other topics with vastly different data structures, time and country periods, and variable selection methods: democratization, regional public good provision, and individual trust in institutions (for more information, see *SI Appendix*, sections S2–S4).

**Fig. 2.**
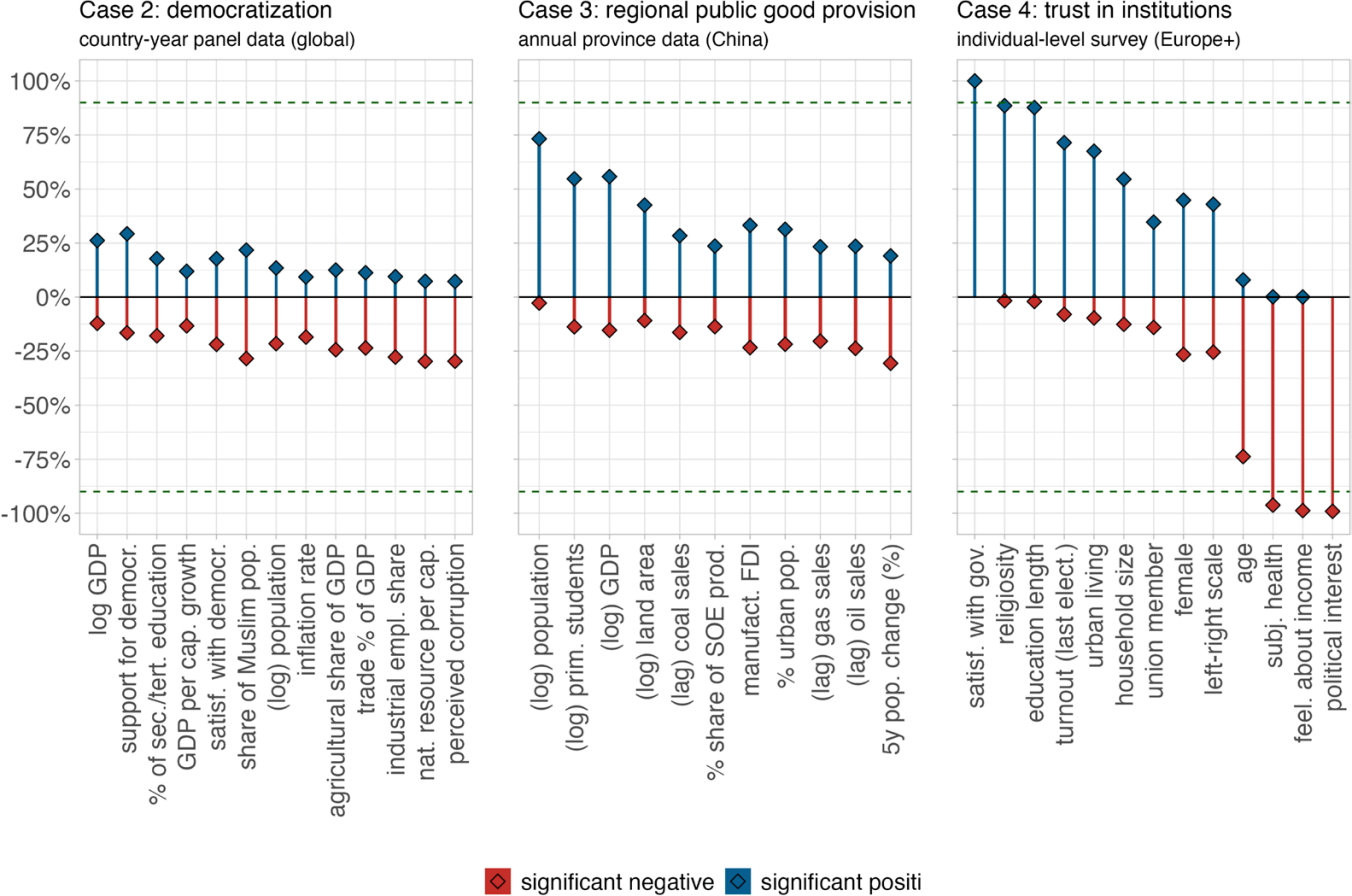
Share of significant coefficients in the model space of the four test cases. The panels present the share of (positive and negative) significant coefficients (blue and red, respectively) of all independent variables in the unrestricted model universe for the three test cases: democratization, regional provision, and institutional trust. The dashed line indicates 90%.

Similar to our main case, our results underscore the prevalence of model uncertainty, particularly in relation to questions surrounding democratization and regional public good provision (Fig. 2). Our analyses do not only reveal relatively low levels of robustness in terms of statistical significance for these cases, but also continue to indicate a high share of coefficients switching signs, i.e., a bidirectional sensitivity in the model space. Put differently, for almost all indicators, we find a substantial probability mass of significant coefficients with both positive and negative signs. Our approach also makes it possible to explore how the significance shares of the independent variables vary by different SE types, fixed effect structures, dependent variables, country samples, and period samples (*SI Appendix*, sections S2.2 and S3.2).

In contrast to the first three cases, the examination of the determinants of institutional trust reveals more robust results, particularly for indicators such as satisfaction with the government, education, or feelings about household income. All of these indicators exhibit the expected signs and seem to be stable predictors when examining individual-level trust in institutions. Thus, a larger sample size contributes not just to more stable findings in terms of significance but also in terms of direction of estimates, underscoring the role of sampling uncertainty in overall model uncertainty (for robustness checks, see *SI Appendix*, section S4.2). The results for the case of institutional trust also demonstrate that our method does not mechanically or necessarily result in a high sensitivity of results.

### The Sources of Model Uncertainty.

**Fig. 3.**
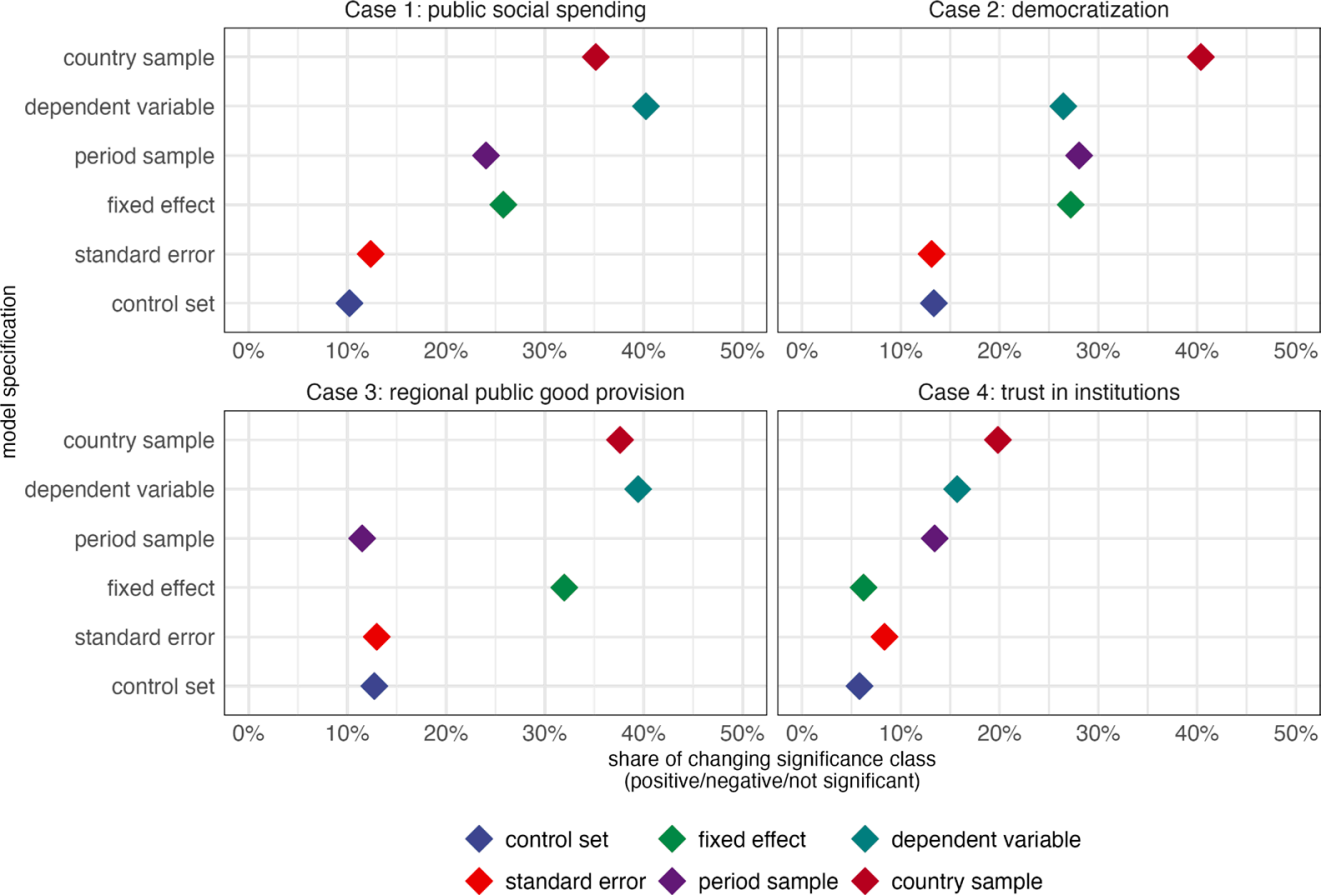
Share of changing significance class of 1-nearest neighbor approach. The figure shows the results of a 1-nearest neighbor simulation applied to the four different cases. For each case and independent variable, we randomly selected a model from the model space and estimated its coefficient, categorizing it into a significance class (negative, positive, or not significant). A single specification was then altered at random, and the new coefficient’s significance class was determined. This process was repeated 50,000 times. The panels report the share of times the significance class changed when altering only one model specification, averaged across the six model specification dimensions (samples, fixed effects, SE types, control set).

In this section, we report the results from three approaches to assessing how different empirical choices impact model uncertainty, captured by the significance and sign of a coefficient for a given independent variable. We provide more detail and compare the strengths and weaknesses of each approach in *Material and Methods* section. As a first test, we apply an approach to describe model sensitivity to one specification choice, which we call a “1-nearest-neighbor approach.” For each case and independent variable, this approach consists of randomly drawing one model from the model space. Next, it compares the significance and sign of the coefficient to the coefficient when randomly changing only one model specification of the original model. This process is repeated 50,000 times, which makes it possible to then calculate how often the significance class (positive, negative, not significant) changes in response to a particular model specification choice (i.e., dependent variable, fixed effect structure, etc.). As shown in Fig. 3, changes of the unit and time sample have a far larger impact on the significance class than alterations in the control set. As we discuss in the *Materials and Methods* section, this approach maximizes the ease of implementation and interpretation, while other approaches capture more subtle shifts in the predicted probability and can model the potentially nonlinear and complex processes underlying predictions of significance.

As a second test, we conduct a multiclass classification exercise using the model specification choices as input features to predict the significance classification of an estimate. The outcome variable, which we call “significance class,” is a categorical variable denoting “positive significant,” “negative significant,” or “not significant” (reference category), based on the sign and *P*-value (significant if *P*-value < 0.1) of the underlying estimate. Predictors are a series of specified binaries assigned a value of 1 if a particular model specification is applied (e.g., whether a specific control variable is included, a certain dependent variable used, etc.) and 0 otherwise. We employ a multinomial logistic regression. While less straightforward to implement and interpret than our first test, it makes it possible to model the marginal and nonlinear effects of different modeling choices on the predictive probability of significance. For reasons of space, the results are presented in *SI Appendix* and discussed in the *Materials and Methods* section.

**Fig. 4.**
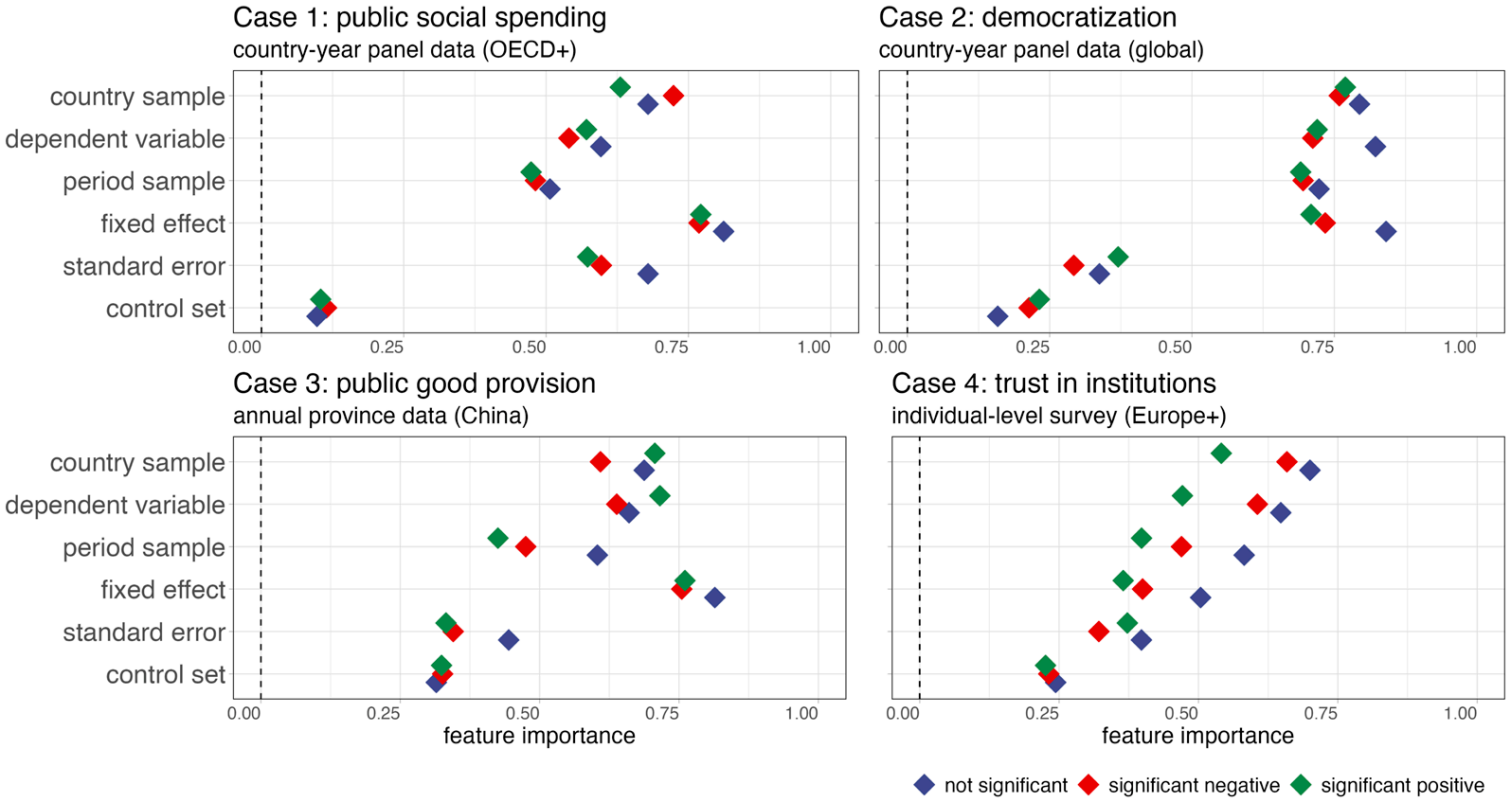
Feature importance scores of model specification decisions. The panels show the feature importance scores (SHAP values) for different model specification choices. To estimate them, we extracted a random set of 250,000 regression coefficients from the unrestricted model universe for each test case. Then, we fit a multilayer neural network to predict whether an estimate is “negative significant,” “positive significant” or “not significant.” After employing a grid-search algorithm to define the most suitable hyperparameter structure, we use the best model with the highest classification accuracy to estimate the SHAP values of each model specification binary.

Third, we adopt a deep learning approach using alternative feature importance measures. As we discuss in the *Materials and Methods* section, this approach achieves very high predictive accuracy while at the same time not making any restrictive assumptions about the underlying functional form linking the inputs (modeling choices) to outputs (significance). However, these advantages come at the costs of being less easy to implement and interpret than the other two approaches. Fig. 4 plots the feature importance for different model specification types, demonstrating the impact of each specification decision on the probability of obtaining a significant positive, significant negative, or nonsignificant coefficient. The results indicate that the choice of the control set plays, for all four test cases, the least important role in the significance classification of a coefficient. Specifically, the selection of the control set yields the lowest feature importance scores (see *SI Appendix*, Figs. S1.5.4, S2.3.4, S3.3.4, and S4.3.4 for feature importance score of single model specification choices), followed by the SE type. In contrast, sampling and the operationalization of the dependent variable carry substantially larger weight for the *P*-value distribution than the covariate set.

While these results are very much in line with what we found in Fig. 3 with our “1-nearest-neighbor” approach, each approach has a distinct strength and limitation. On the one hand, the 1-nearest-neighbor approach is easier to interpret and focuses on big changes in significance (either present or absent). On the other hand, the neural network models are able to capture even small marginal effects on probability of significance and they do not hold all model specifications constant. The results largely corroborate our main finding that model uncertainty primarily arises from sampling and measurement considerations rather than the conditioning aspect, at least in the four cases we consider in this article (*SI Appendix*, sections S1.3, S2.3, S3.3, and S4.3).

## Discussion

Combining the relative strengths of the Extreme Bounds Analysis and the multiverse approach (1, 2, 7, 13, 16–19), this article has developed a method that evaluates the extent, nature, and sources of model uncertainty across multiple dimensions, including the time period and unit coverage samples, dependent variable, control set, as well as the fixed effect and SE structure. An examination of the robustness of coefficients across four prominent topics in political science—welfare state generosity, democratization, public good provision, and trust in institutions—reveals that estimates are highly sensitive to changes in the underlying model structure. This suggests that quantitative analyses in political science may be subject to significantly higher levels of model uncertainty than what more conventional sensitivity analysis approaches typically can account for. Specifically, our findings reveal a substantial probability mass of statistically significant coefficients with both negative and positive signs for the majority of independent variables. This bidirectional nature of the model space implies that minor alterations in the model specification can yield estimates pointing in theoretically opposing directions.

One should not necessarily nor automatically conclude from this analysis that none of the factors under consideration are associated with each outcome. Instead, our results highlight the importance of considering a wider range of sensitivity analyses when assessing robustness. Regardless of which of three approaches to estimating the sources of sensitivity is adopted, our main contribution is to show that evaluating the robustness of quantitative results requires a comprehensive examination of model specification choices beyond the selection of the covariate space. Crucially, we find that model uncertainty stems primarily from issues related to sampling and measurement, rather than conditioning. In other words, the sign and statistical significance of estimates is more contingent on the underlying sample and the operationalization of indicators than on the inclusion of the control set on the right-hand side. This insight is particularly noteworthy, especially in light of longstanding concerns about “researcher degrees of freedom” (14, 23–27) and considering that existing sensitivity approaches often do not systematically evaluate coefficient fragility across many dimensions exhaustively and simultaneously.

While we have tried to consider very different cases in terms of data structure, sample size, unit of observations, time period, and geographical coverage, we cannot ascertain the extent to which our four cases are representative of the wide range of datasets and debates in the social sciences. On the one hand, focusing on a few cases might not capture the true range of all possible datasets and debates one could explore, thereby underestimating the degree of sensitivity. On the other hand, it is also possible that other social science debates are more “settled” than the ones considered here, so sensitivity could therefore be lower. Unfortunately, we do not believe there exists an uncontroversial metric for us to objectively assess how settled a particular debate is. As a result, we leave it for future research to apply our method to other topics in the social sciences.

One limitation of our selected case studies is their reliance on classic regression analysis of nonrandomized, observational data. Thus, our results primarily evaluate robustness from a correlational perspective applied to a range of observational data. Causal methods have rightfully gained increased attention in the last two decades. Yet, correlational approaches have been a prevalent methodology in the social sciences for much of the second half of the twentieth century. They also remain an integral and substantial methodological toolkit in the study of many crucial topics in the social sciences, particularly in domains where (full or quasi-) randomization is impractical or impossible, and/or where external validity is considered more relevant. Our article could also be used for more causal approaches, where the prevalence of model uncertainty has started to be explored (28, 29).

In response to the challenge of model uncertainty in the political sciences and beyond, we propose several ways forward. Recognizing the limitations of existing sensitivity approaches, our proposed method along with our accompanying R library aims to assist applied researchers to identify the sources of model uncertainty, in terms of the empirical decisions that matter most. The results from our simulation exercise have shown that it does not require extensive time and computing power. Thus, researchers facing too many dimensions with limited computing power can opt for a reduced form approach focusing on a random subset of the universe of all possible combinations, which approximates the overall distribution of sensitivity. As more cases are considered, the field will be in a position to assess the generalizability of the results we present in this article. Using the methods in this paper should go hand in hand with an increased emphasis on theory building to enhance the quality of model selection for specific research questions, especially for parts of the empirical approach where greater sensitivity is detected. Grounding empirical decisions more explicitly in previous theoretical contributions or systematic literature reviews (30) as well as statistical theory can help inform which combinations of choices are more or less warranted. Finally, we have proposed three different approaches that researchers can employ to more clearly model what is driving sensitivity in their results.

## Materials and Methods

### Case Selection.

To evaluate and illustrate the value of our method, we examine the robustness of a diverse set of factors influencing four outcomes that have received extensive attention in political science: welfare state generosity, democratization, public good provision, and individuals’ trust in institutions (e.g., refs. 31–34). For each of these four topics, we select relevant quantitative variables (for an overview, see *SI Appendix*, section S0). First, for the case of the welfare state, we conducted our own extensive literature review, enabling us to identify the dependent and independent variables based on a wide range of previous theoretical and empirical contributions over the last decades. We then relied on a frequently used country-year dataset covering 33 developed economies (see *SI Appendix*, section S1.1 for more information). Second, for the case of institutional trust, we have used a previous literature review (35) to select variables and we then run our analyses on all waves of the European Social Survey—one of the most cited cross-country surveys (see *SI Appendix*, section S4.1 for more information). Third and fourth, we use the replication datasets of two recent excellent articles on democratization across the world and on regional public good provision, respectively (36, 37).^*^ We provide a detailed description of each dataset and the selected variables in *SI Appendix*, sections S1.1–S4.1.

Our case selection enables us to compare how our method performs when applied to various sample sizes and data structures, including regional panel, time-series cross-section for OECD+ countries, global panel, and survey data (see *SI Appendix*, section S0 for more information). This allows us to assess how model uncertainty fluctuates with different types of data and also to illustrate how institutional (i.e., democratization), government (welfare state generosity, regional public good provision), and individual behavior (trust in institutions) may be more or less subject to model uncertainty. In addition, our cases differ with respect to geographical coverage to provide evidence for a broader set of countries and regions, which is why we resorted to datasets that cover China (regional public good provision), Europe (individuals’ trust in institutions), OECD countries (welfare state generosity), and the whole world (democratization).

The next sections explain in more detail our methodological approach to quantifying the extent and nature of model uncertainty, the robustness checks and different restricted model spaces, and how we estimate the sources of model uncertainty.

### Quantifying the Extent and Nature of Unrestricted Model Uncertainty.

Building upon Leamer’s (1, 2) systematic approach to assess robustness toward the covariate space (*SI Appendix*, section S0.3), we expand its scope by incorporating additional empirical specification choices, specifically focusing on unit samples, period samples, operationalization of the dependent variable, fixed effect structure, and the type of SE. The rationale behind selecting these specification choices lies in their frequent use in quantitative social science research. Formally, our approach is based on the following linear model for each unit-period sample (denoted as s):

$$Y_{d}=\alpha_{0}+\beta_{1}X+\beta_{k,p}C_{k,p}+\vartheta_{i}+\delta_{t}+\epsilon ,$$

where *Y_d_* is the dependent variable where the subscript *d* reflects different operationalizations of the outcome variable *Y*; *ϑ_i_* and *δ_t_* are unit and time fixed effects, respectively; *β*_1_ is the estimated coefficient for the independent variable of interest, *X*; and *β_k_* are the coefficients vector of covariate space *C_k_* with *p* indicating the set of control variables *C* that is included in the estimation. We rerun the analysis with/without *ϑ_i_* and/or *δ_t_*. We then extract *β*_1_ (together with the accompanying SE*ϵ*) over all specifications of *d*, *s,* and *p* to construct the extreme bounds and significance shares for each independent variable *X*. Although we run this approach on all four cases varying the same number of dimensions, the total number of combinations depends particularly on how many independent variables are included. For instance, in the case of the welfare state, we have n=18 independent variables, so we create the following number of control sets:

∑k=1nCk(n)=∑k=1nnk=∑k=1nn!k!n-k!=2n-1=262,143.

Each of these sets is used in combination with seven country samples, three different time samples, four different combinations of country and time fixed effects structures, three types of SE, and six different dependent variables. Thus, in total, with 262,143 control sets and 1,512 (since 21 × 4 × 3 × 6 = 1,512) model specifications, we end up with 396,360,216 regressions in the whole model space. These regressions include over 3.56 billion estimates (coefficient and SE combinations) with 198,180,108 estimators for each of the 18 independent variables.

While the incorporation of numerous model choices may be theoretically and empirically desirable to comprehensively test the robustness of a relationship of interest, we acknowledge that the size of the model space may not be suitable for most scholars facing computational limitations. We provide an empirical demonstration for two potential solutions to this problem. First, the computing demands are a direct function of the number of model specifications under consideration. This quantity rises exponentially with the number of covariates, so the most effective way of increasing efficiency is to decrease the number of controls included in the analysis. We show that reducing the number of independent variables leads the number of regressions models to fall from 396 m (for the case of the welfare state) to smaller model spaces for the other three cases, ranging between 9 m and 12 m regressions depending on the case. A model space of this size can be easily estimated on a normal personal computer. Thus, even a full model space encompassing all possible combinations is feasible so long as the number of dimensions are not excessively large.

Second, we provide an empirical demonstration that imposing random reductions in the size of the full model space does not alter the key findings. Specifically, we map out the combinations of the 18 control variables for the case of the welfare state, alongside 1,512 other model specification choices to randomly select 150 model spaces for each independent variable. For each covariate-specification vector, we then construct and estimate the model space to determine the relevant significant shares (similar to the baseline analysis, see *SI Appendix*, section S1.1). These shares vary with the length of the covariate-specification vector, reflecting the size of the model space. This approach yields 150 significance shares for each independent variable based on 150 randomly generated but smaller model spaces. To assess how closely these smaller model spaces approximate the full model universe, we average the significance shares from the smaller spaces and compare them with the full set.

*SI Appendix*, Fig. S1.2.7 illustrates the significance shares of the welfare state determinants under consideration. The results from the reduced-form simulation approach indicate that the average significance shares from the smaller model spaces closely align with those of the full model space across all independent variables. In other words, even reduced model spaces converge relatively quickly to the values of the unrestricted model space. These findings suggest that neither the size nor the composition of the model space significantly alters our conclusions. Since averaging across smaller model spaces is more computational efficient, this makes it possible to implement the method on personal computers even for a very large set of modeling choices. In addition, these results further help alleviate concerns that with an extensive model space there would be a risk that a few specific model specifications might produce a disproportionately large share of insignificant estimates. This could misleadingly indicate high model uncertainty, when in fact it may be attributable to the inclusion of these specific but problematic choices. If this was the case, our initial results might appear overly pessimistic—but we find no evidence for this concern.

### Defining Restricted Model Spaces.

One criticism of the unrestricted EBA results is that there might be a large fraction of misspecified models with respect to empirical and/or statistical properties. If this is the case, the unrestricted model space can skew the results in both directions and thus bias the conclusions. We carry out three tests to explore whether this is an issue. First, we impose a priori restrictions on the unrestricted model universe by removing all estimates which fail to meet certain criteria. Specifically, we exclude all regression models that suffer from multicollinearity by dropping all regressions in which the independent variable of interest has a Pearson correlation coefficient (absolute size) that is larger than 0.5 with one (or more) covariates. In addition, we also drop all welfare state regressions which include more than one partisanship variable. After calculating the significance shares of this restricted model universe, we show that the results are very similar to the findings we derived from the unrestricted model universe (*SI Appendix*, Fig. S1.3.1).

Second, in addition to removing misspecified models that suffer from double measurement or multicollinearity, one can also shrink the model universe to estimations based on a goodness of fit measure. In this way, we remove the models with low explanatory power and only focus on the ones that can, in relative terms, more accurately model the outcome variable of interest. When we restrict the unrestricted model universe to the 10% best fitted models based on the Akaike Information Criterion, our results are very similar to the unrestricted baseline (*SI Appendix*, Fig. S1.3.2). In fact, most variables indicate smaller significance shares and we still find a sizable number of positive and negative coefficients—with the exception for the crisis dummy—for all determinants under consideration.

Third, instead of imposing restrictions on the model universe, we adopt a weighting approach that weighs the individual estimates with their model’s respective likelihood ratio index. This allows us to construct the cumulative density function to determine the robustness/fragility of an explanatory variable of interest. This approach comes with the advantage that it exploits the entire distribution of estimates without a priori setting potentially arbitrary or ad hoc exclusion criteria. Again, the results are in line with our baseline finding (*SI Appendix*, Figs. S1.3.3 and S1.3.4).

### Estimating the Sources of Model Uncertainty.

In addition to the systematic method for estimating sensitivity to modeling choices, we also apply several approaches to exploring the sources of this model uncertainty. This will allow researchers to understand the varied impact of different empirical choices on the final significance and sign of a coefficient for a given independent variable. We employ three approaches to assess these sources. The first is based on simulating different combinations of model choices and then describing the variation in full significance switches, while the other two implement classification models to predict the direction and statistical significance of a coefficient: a multinomial logistic regression and a deep learning approach. We discuss each in turn and compare their relative strengths and weaknesses.

In the first approach, we adopt a descriptive simulation of different random draws, which we call a 1-nearest neighbor approach. Specifically, for each case and each independent variable, we randomly draw a model from the model space. Next, we estimate the coefficient and define the significance class (negative, positive, not significant). Then, we randomly change one single specification of this original model, and we again determine the significance class of this new coefficient. We repeat this process 50,000 times. Finally, we calculate the share of how often the significance class fully changes when altering one model specification only.

In our second and third approaches, we conduct a multiclass classification exercise using the model specification choices as input features to predict the significance classification of an estimate. The outcome variable, which we call “significance class,” is a categorical variable denoting “positive significant,” “negative significant,” or “not significant” (reference category), based on the sign and *P*-value (significant if < 0.1) of the underlying estimate. Predictors are specified binaries assigned a value of 1 if a particular model specification is applied (e.g., whether a specific control variable is included, a certain dependent variable used, etc.) and 0 otherwise. The analytical problem is how best to model this predictive problem to identify the most important predictors of model uncertainty. We employ two estimation strategies: multinomial logistic regression and neural network/deep learning models.

Our second approach is to estimate a multinomial logistic regression to model the absolute change in predicted probabilities, measuring how the probabilities of an outcome change with a specific model specification. Specifically, to estimate these average changes in predicted probabilities, we extracted a random set of 250,000 regression coefficients from the full model space and then fit a multinomial logistic regression to predict whether an estimate is “negative significant,” “positive significant,” or “not significant.” Next, we estimate the average change in predicted probabilities to assess the relevance of each model specification choice (binary).

As our third approach, we use machine learning to more flexibly model the nonlinearities and interactive relationships between specification choices, and in this way enhance the predictive power of the classifier. Using the same random set of estimations as for the multinomial logistic, we employ a deep learning approach to build a classifier that predicts whether an estimate is “negative significant,” “positive significant” or “not significant.” By adopting a grid search approach combined with the careful tuning of hyperparameters, we aim to find the most suitable model structure and hyperparameters for each independent variable under considerations. In this way, we are able to identify a model structure that is robust and generalizes well to new, unseen data in the test set. We provide further information about the structure and details of the neural network in *SI Appendix*, section S5. To assess the importance of each specification binary, we select the best-performing model structure based on its classification accuracy, and then use this model to estimate SHAP (SHapley Additive exPlanations) values, proposed by Lundberg and Lee (38). These values determine the influence of each feature (in our case: the model specification binaries) on the prediction of the significance class. More specifically, the SHAP values are a method used to interpret the output of machine learning models by quantifying the contribution of each feature to a specific prediction. Based on cooperative game theory, SHAP values allocate the total prediction difference (between the model’s output and the average prediction) fairly across all features. Each feature’s SHAP value represents its individual contribution to the outcome, ensuring both consistency and local accuracy. This method allows for detailed, instance-level interpretation and can be aggregated across multiple predictions to understand the overall importance and impact of features in a model, making it ideal for both local and global interpretability.

As a robustness check, we also use the feature effect approach from the FeatureEffect R library to compute and plot individual feature effects on prediction models (39–41). We choose the Accumulated Local Effects (ALE) plots because they are computed as accumulated differences over the conditional distribution of features, making them more reliable in the presence of correlated features compared to Partial Dependence Plots. ALE plots break the feature space into small intervals and compute the effect of the feature within these intervals. The choice of ALE plots allows us to accurately capture nonlinear interactions and localized effects without bias from correlated features, which ensures more faithful representation of variable impacts. As the results in *SI Appendix*, Fig. S5.1.1 show, the country sample and dependent variables are the most important in three out of four cases, while the control set is the least important feature in all cases, with period sample. Fixed effects and SE choices occupy intermediary positions in most cases. Both the SHAP values and the ALE approach highlight that sampling and measurement has higher relevance for the significance class than the covariate set.

Although the results of the 1-nearest neighbor approach (Fig. 3) are broadly in line with what our multinomial logistic regression and our deep learning approach find (Fig. 4), each has distinct strengths and shortcomings which are presented in Table 1. First, in terms of ease of implementation and interpretation, the 1-nearest approach scores highest as it is most intuitive in capturing full switches in significance level. Next, the multinomial logistic regression displays a slightly more complicated approach since it estimates the marginal effects of different modeling choices on the predicted probability of a significance class (instead of the actual significance class), thereby capturing more subtle effects but complicating interpretation. The deep learning approach, while powerful in other aspects, is more challenging to implement and interpret. A key issue is that the scale of ML-based feature importance scores is less intuitive.

**Table 1.** The strengths and weaknesses of different approaches to modeling the sources of sensitivity

Strengths/weakness: Approach:	Ease of implementation and interpretation	Nonlinearities and complex interactions in prediction	Focuses on full switch in significance versus marginal effects on predicted probability	Predictive accuracy
Nearest 1-neighbor	High	Low	Full switch	Not measurable
Multinomial logistic regression	Medium	Medium	M.E. on pred. prob.	Medium
Deep learning approach	Low	High	M.E. on pred. prob.	High

Second, in terms of the underlying complexity and nonlinearity of the assumed relationship, the performance is reversed: While the deep learning approach is the most versatile and can accommodate all functional forms and nonlinearities by building a predictive model with multiple layers, nodes, and activation functions, the multinomial logistic approach makes more restrictive assumptions concerning the function form and linearity. Similarly, the nearest-1 neighbor approach by design allows for no complex relationships, since it does not evaluate sensitivity with reference to any underlying relationship with other modeling choices but only changes one specification choice at a time.

Third, linked to functional form on the right-hand side of a relationship, there are also key differences in the ways the key outcomes are conceptualized. In the nearest 1 neighbor approach, a change in a particular dimension either changes or does not change significance on the left-hand side. In a second step, these changes are then averaged across a large number of random changes in other parts of the specifications. Yet, this does not allow for marginal effects, for instance, that change the predicted probability of observing a particular significance class of a given coefficient. This is precisely what the other two approaches allow by setting up the problem as one of predictive rather than descriptive nature. Both approaches aim to model the predicted probability of a significance class as a function of multiple modeling choices in a first step, and then in a second step they estimate the marginal effects of different characteristics on the predicted probability (in the case of multinomial logistic regressions).

Finally, in contrast to the nearest 1-neighbor approach, the logistic and deep-learning can be evaluated based on standard measures quantifying predictive power. The neural networks clearly outperform multinomial logistic regression on all conventional measures. Indeed, the former shows good performance as the precision, recall, accuracy, and F1-score are consistently above 90% (*SI Appendix*, Fig. S5.1.2), indicating that the grid-search approach found well-performing models for all four cases under consideration. By comparison, the precision and recall (by class) of the multinomial logistic regression compares less favorably (*SI Appendix*, Fig. S5.1.3). The model performance is overall good but exhibits important variation, not least because this approach cannot model all nonlinearities/interaction effects simultaneously (due to the underlying linearity assumption of a multinomial logistic approach).

## Supplementary Material

Appendix 01 (PDF)

## Data Availability

All our code will be freely available (with no restrictions) on GitHub (https://doi.org/10.5281/zenodo.15480536) (42). In addition, the data for the case on the welfare state will also be available in our GitHub repository. For the two replication studies and the ESS survey dataset, we are not able to upload these datasets separately. However, all three datasets can be requested/downloaded free of charge from the following webpages: European Social Survey (ESS) from the ESS Data Portal: https://www.europeansocialsurvey.org/data-portal (43). Claassen, C. (2020). “Does Public Support Help Democracy Survive?” from Harvard Dataverse: https://dataverse.harvard.edu/dataset.xhtml?persistentId=doi:10.7910/DVN/HWLW0J (44). Hong, J. Y. (2018). “How Natural Resources Affect Authoritarian Leaders’ Provision of Public Services: Evidence from China.” from Harvard Dataverse: https://dataverse.harvard.edu/dataset.xhtml;jsessionid=3de4b986d641429e95902a1efc7a?persistentId=doi%3A10.7910%2FDVN%2FIZC5P1&version=&q=&fileTypeGroupFacet=&fileAccess=Public&fileSortField=type (45).

## Footnotes

^*^In these two datasets (see *SI Appendix*, sections S2.1 and S3.1 for more information), we include the main control variables that the authors themselves use in their own studies, but want to emphasize that our purpose is not to corroborate nor invalidate the excellent analyses conducted in these two articles, but instead to illustrate how our approach can help map out the relevant choices and assess sensitivity to these choices. Thus, our method provides a toolkit for applied researchers, and only represents a necessary but not sufficient set of results to evaluate robustness.
