# Supplementary material for: Estimating the extent and sources of model uncertainty in political science
Source: Proc Natl Acad Sci U S A. 2025 Jun 17;122(25):e2414926122. doi: 10.1073/pnas.2414926122 (PMC12207428; doi:10.1073/pnas.2414926122)
Supplement: Supplementary file 1 — Appendix 01 (PDF) [file pnas.2414926122.sapp.pdf]

PNAS

Supporting Information for

Estimating the Extent and Sources of Model Uncertainty in Political Science

Authors:
Michael Ganslmeier*^
Tim Vlandas^^

Affiliations:
^ Centre for Computational Social Science, University of Exeter, Address: Stocker Rd, Exeter EX4 4PY, United Kingdom

^^St Antony's College and Department of Social Policy and Intervention, University of Oxford. Address: 32 Wellington Square, OX1 2ER Oxford, United Kingdom.

***Corresponding author:** Michael Ganslmeier.
Email: m.ganslmeier@exeter.ac.uk

This PDF file includes:

Supporting text
Figures S1.2.1 to S5.1.3
Tables S0.2.1 to S4.1.1
SI References

S0: GENERAL REMARKS ABOUT DATA AND METHODS 3

S0.1. Introduction 3
S0.2. Summary cases and specifications 4
S0.3. Measuring model uncertainty 5

S1: CASE 1: WELFARE STATE DEVELOPMENT 6

S1.1: Literature review and data selection 6
S1.2: Significance shares by model specifications 10
S1.3: Significance shares of the restricted and weighted model space 17
S1.4: Impact of model specification on significance of estimates 21

S2: CASE 2: DEMOCRATIZATION 25

S2.1: Literature review and data selection 25
S2.2: Significance shares by model specifications 28
S2.3: Impact of model specification on significance of estimates 34

S3: CASE 3: REGIONAL PUBLIC GOOD PROVISION 38

S3.1: Literature review and data selection 38
S3.2: Significance shares by model specifications 41
S3.3: Impact of model specification on significance of estimates 47

S4: CASE 4: INDIVIDUAL TRUST IN INSTITUTIONS 51

S4.1: Literature review and data selection 51
S4.2: Significance shares by model specifications 54
S4.3: Impact of model specification on significance of estimates 60

S5: DETAILS ABOUT THE NEURAL NETWORKS AND THE GRID SEARCH APPROACH 64

S6: AN R PACKAGE FOR EMPIRICAL SOCIAL SCIENCE 68

S7: SI REFERENCES 69

S0: General remarks about data and methods

S0.1. Introduction

In this Supplementary Information (SI), we provide comprehensive details for each selected case study, elaborating on the identification and selection of independent variables, the datasets utilized, and the extensive model space explored for each case (see Table S0.2.1 and S0.2.2 below). We aim to enhance the transparency and reproducibility of our findings by offering an exhaustive description of the data sources, the modeling decisions made, and our analysis.

In the rest of this section (S0), we discuss the general framework of our analysis, highlighting the overarching data and methodological choices made throughout the study. The current sub-section S0.1 serves as an introduction, providing context for the approach used in the case studies. Next, sub-section S0.2 summarizes the various cases and model specifications that were employed. Finally, sub-section S0.3 elaborates on the approach taken to measure model uncertainty, detailing how changes in specification can lead to shifts in the results.

Each case study, discussed in sections S1 to S4, respectively, follows the same structure for consistency. First, in section S1 on Welfare State Development, we begin with a literature review and a discussion of data selection (sub-section S1.1) to ensure a comprehensive and well-motivated identification of the independent variables. Second, sub-sections S1.2 and S1.3 then present significance shares by model specifications, showing the impact of modelling choices on statistical significance, both in the unrestricted, restricted and weighted model universe. The other case studies (S2, S3, S4) follow a similar structure: section S2 covers democratization, section S3 focuses on regional public good provision, and section S4 introduces individual trust in institutions. Each case highlights the variability in outcomes due to different model choices, providing insights into the uncertainty of empirical research in the social sciences.

In section S5, we describe the machine learning approach used to assess model uncertainty, focusing on a deep learning model that classifies estimates into significance categories. The model is optimized using a grid search approach with hyperparameter tuning to achieve high predictive performance, accounting for non-linearities and interactions between model specification choices. We employ a feedforward neural network with multiple hidden layers, trained and validated on split datasets to ensure robustness. The final model achieves strong accuracy and F1-scores, and SHAP values are estimated to highlight the influence of each specification choice on significance outcomes.

In section S6, we provide information about the associated R package developed to facilitate the application of our empirical approach to future social science research. Specifically, this package is intended to assist researchers in conducting model uncertainty analysis, enabling the exploration of vast model spaces in an efficient and reproducible manner. Detailed instructions on how to install and use the package are provided, along with example workflows that mirror the analyses presented in this study.

S0.2. Summary cases and specifications

Table S0.2.1. Summary of cases.

No	Outcome	Units	Region	Time period	Data type	Level
1	welfare state generosity	33 countries	OECD+	1980-2016	panel	country
2	democratization	135 countries	Global	1987-2017	panel	country
3	public good provision	31 provinces	China	1992-2010	panel	province
4	trust in institutions	39 countries	Europe+	2002-2020	survey	individual

Table S0.2.2. Summary of model specifications per case.

No	Independent variables	Dependent variables	Unit samples	Period samples	Estimates samples
1	18	6	7	3	3.57bn
2	12	4	7	4	33.0m
3	11	9	7	3	25.5m
4	15	4	5	5	294.9m

S0.3. Measuring model uncertainty

Leamer's Extreme Bounds Analysis (EBA) method was one of the first attempts to quantify model uncertainty (Leamer, 1983, 1985). Unlike traditional approaches that rely on predefined sets of covariates, EBA systematically varies the inclusion and exclusion of specific control variables to comprehensively assess how the control variable selection impacts the variability in the statistical significance of an estimand of interest. In the original iteration of EBA, Leamer departs from a linear model in the form of:

$$Y_j = \alpha_j + \widehat{\beta_{X_J}} X + \widehat{\gamma_{F_J}} F + \widehat{\delta_{D_J}} D + \widehat{\epsilon_J}$$

where Y_j is the dependent variable of model specification j; X the independent variable of interest (focus variable); D a set of doubtful control variables; F a vector of fixed covariates (free variables) and which are included in all regression models; and ϵ as the error term. After each regressor is assigned to one of these buckets (X, F, D), the researcher permutates over all possible combinations of D and estimates the coefficients and standard deviations of X. The model universe M_j with $j = \{1, \dots, m\}$ then serves to determine the extreme bounds $B_{lower} = \min(\widehat{\beta_{X_J}} - \tau\widehat{\sigma_{X_J}})$ and $B_{upper} = \max(\widehat{\beta_{X_J}} + \tau\widehat{\sigma_{X_J}})$, which are used to assign the robustness/fragility classification to a given X. With a pre-determined test statistic τ, X is robust if $(B_{lower} \times B_{upper}) > 0$, and fragile otherwise. Beyond the binary classification of X, the range $|B_{lower} - B_{upper}|$ represents a general measurement for misspecification uncertainty (Leamer 1983), while a smaller (larger) range refers to smaller (larger) model uncertainty.

Building upon Leamer's established approach, we expand its scope by incorporating additional empirical specification choices, specifically focusing on (i) unit samples, (ii) period samples, (iii) operationalization of the dependent variable, (iv) fixed effect structure, and (v) the type of standard errors. The rationale behind selecting these specification choices lies in their frequent use in quantitative social science research. While the decisions related to subsampling and the operationalization of the dependent variable often stem from theoretical debates, the selection of the fixed effect structure and the type of standard error is a widely debated methodological issue within the social sciences (King and Roberts, 2015; Abadie et al., 2017; Neumayer and Plümper, 2017). Note that we do not consider the inclusion/exclusion of a lagged dependent variable (LDV) for three reasons: first, including an LDV would create a Nickell bias when including unit fixed effects; second, we already consider first differencing the dependent variable; and third, the LDV is often mechanically highly significant while risking absorbing the effects of other variables. Although not reported here, we have also run a version of our analyses of welfare state determinants which confirm that the LDV is the most stable variable and leads to lower significant shares of other variables.

Formally, our approach is based on the following linear model for each unit-period sample, denoted as s,

$$Y_d = \alpha_0 + \beta_1 X + \beta_{k,p} C_{k,p} + \vartheta_i + \delta_t + \epsilon$$

with Y_d as the dependent variable where the subscript d reflects different operationalizations of the outcome variable Y ; ϑ_i and δ_t as unit and time fixed effects, respectively; β_1 as the estimate of interest for the independent variable of interest, X; β_k as the coefficient vector of covariate space C_k with p indicating the set of control variables C that is included in the estimation. We re-run the analysis with/without ϑ_i and/or δ_t. We then extract β_1 (together with the accompanying standard errors) over all specifications of d, s and p to construct the extreme bounds and significance shares for each independent variable X.

S1: Case 1: Welfare State Development

S1.1: Literature review and data selection

While conducting a fully inclusive and comprehensive literature review is beyond the scope of this study, the determinants of welfare state development can be broadly classified into four main categories. Table S1.1.1 provides an overview of the different strands in the welfare state literature with examples of prominent references for each strand. First, domestic economic factors have often been used to advance functionalist arguments, highlighting the roles of economic growth, urbanization, (de-)industrialization, and democratization. Economic development and modernization, for instance, are argued to create the fiscal capacity necessary to expand the welfare state while also creating new risks leading to demands for more protection.

Second, the factors that capture international economic dynamics have aimed to account for the effects of globalization in goods and services, financialization, or Europeanization. Earlier analyses saw various forms of integration into the international economy as increasing the power of capital and limiting the ability of states to tax economic actors to expand the welfare state. Conversely, many prominent studies contend that internationalization heightens the risks of several key groups, leading them to demand more protection.

Third, political factors — most notably partisanship, trade union strength, and the organization and coordination of employers and firms — emphasize the relevance of the interests of political and economic actors. While trade unions and social democratic parties were traditionally seen as representing the interests of the working and lower middle classes in demanding greater decommodification and social services, recent studies question whether this is still the case or if these actors increasingly represent the interests of so-called insiders. In this latter view, the focus shifts to employment protection legislation, while all political parties neglect the interests of labor market outsiders. Similarly, a debate about the role of employers has been ongoing for several decades, particularly whether they always oppose all forms of welfare state policies or whether this depends on the type of capitalism in which they are located and/or the efficiency implications of these interventions.

Fourth, institutionalist approaches have investigated the effect of political institutions, such as state structures and veto players, or economic institutions, on welfare state generosity. Highly fragmented political systems appear less conducive to reforming the welfare state. At the same time, more inclusive (proportional representation) electoral systems may encourage all parties to cater to a wider spectrum of social risks, thereby leading to more redistribution and larger welfare states.

We apply our method first to the literature on the determinants of welfare state generosity, capitalizing on the many theoretical debates and disagreements in this topic. This approach affords us the flexibility to investigate a broad spectrum of plausible model specifications. For instance, as previous scholars have asserted, a country's welfare state regime plays a pivotal role in shaping the relevance of specific factors. Thus, one can expect a priori that a specific determinant can be more or less relevant in certain geographical and temporal contexts than in others.

Another prominent debate, referred to as the dependent variable problem, revolves around the proper conceptualization, operationalization, and measurement of generosity (Allan & Scruggs, 2004; Clasen & Siegel, 2007). Scholars have long debated whether spending- or entitlement-based indicators offer a more suitable framework for effectively capturing social policy outcomes.

Therefore, the study of welfare state determinants is an ideal case to assess empirical model uncertainty due to its relatively high level of theoretical ambiguity and large empirical literature.

To test the relevance of these empirical choices, we construct a panel dataset consisting of country-year observations covering 33 countries between 1960 and 2016 by selecting key independent variables based this in-depth literature review. Although the selection of independent variables requires some (inevitably subjective) judgment calls and may be influenced by the authors' understanding of the literature, many of these variables and data sources have been widely used in previous comparative empirical studies of the welfare state. Additionally, focusing on a finite number of determinants is necessary as the potential model space increases exponentially with the number of independent variables, which would quickly exceed the technical capacities even of the high-performance computing environments available to the authors. Based on this literature review, we select 18 independent variables to examine for model uncertainty. A description of all independent variables including summary statistics is provided in Table S1.1.2. We source the majority of variables from the Comparative Political Dataset (Armingeon et al., 2013) with the exception of GDP per capita (IMF, 2021) and index of corporatism (Jahn, 2016).

In addition to the control set, we define five further sets of model specifications. First, for the unit samples, we use the regime classification provided by Ferrera (1996), which consists of seven country sub-samples: Anglo-Saxon, Bismarkian, Scandinavian, Southern European, South-Eastern European, all countries without South-Eastern European welfare state regimes, and all countries. Second, we split each of these country datasets along three time periods: 1980-2000, 2000-2016, and 1980-2016. In this way, we end up with a total of 21 country-year panel datasets (7 x 3).

Third, to partially account for debates about the correct conceptualization and operationalization of the outcome variable, we estimate the model space with three different measures of welfare state generosity: total social expenditure as a percentage of GDP and social transfers as a percentage of GDP (both taken from Armingeon et al., 2013), as well as an index of welfare state entitlement (Scruggs, 2014). For each of these indicators, we run the sensitivity analysis in levels as well as their first differences, resulting in six different outcome measures.

Finally, fourth and fifth, we apply different fixed effect structures and standard error types. Specifically, we use four different fixed effect combinations: year fixed effects only, country fixed effects only, country and year fixed effects, and no fixed effects. Next, we use three types of standard errors: simple/unadjusted standard error, heteroscedasticity-robust standard error, and the Huber-White standard error clustered at the country level.

With 18 independent variables, we create a control set for each combination of variables, resulting in a total of 262,143 control sets. Each of these sets is used in combination with seven country samples, three period samples, six dependent variables, four fixed effect structures, and three standard error types. Thus, in total, with 262,143 control sets and 1,512 model specifications (7 country samples × 3 period samples × 6 dependent variables × 4 fixed effect structures × 3 standard error types), we end up with 396,360,216 regressions in the entire model space. These regressions include approximately 3.57 billion estimates (coefficient and standard error combinations).

Table S1.1.1. Overview of previous welfare state literature.

Main strands	Key claim	Periods	Key Authors	Variables
Functionalist	Economic developments drive welfare state development	1960s-1970s	Cutright 1965; Jackman 1974; Wilensky and Lebeaux 1958; Wilensky 1974	Economic growth, industrialization, urbanization
Marxist	Welfare state serves legitimation and accumulation functions	1960s-1970s	Offe 1972; Gough 1979; Bowles and Gintis 1982; Piven and Cloward 1982; Miliband 1969, Domhoff 1972; Block 1977; Prezworski 1986	Capitalist development, elites in government, structural dependence on capitalists
Democratisation	Democracy leads to more welfare state spending	1960s-1980s	Briggs 1961; Marshall and Bottomore 1992; Flora and Heidenheimer 1981; Haggard and Kaufmann 2020	Suffrage, democratic institutions, presence of civil rights
Power resources	Strength of labour movement leads to welfare state expansion	1970s-1980s	Cameron 1978; Shalev 1983; Castles 1993; Korpi 1978, 1980, 1985; Stephens 1979; Esping-Andersen 1990; Hicks 1999	Left control of cabinet and parliament, union density and bargaining coverage, organization of union movement
Regime approach	Historical class coalition dynamics institutionalize policies	1990s-2000s	Esping-Andersen 1990; Ferragina and Seelb-Kaiser 2011; Van Kersbergen and Vis 2015; Arts and Gelissen 2010	Nature of rural-urban and religious cleavage
Statist	Explains cross-national variation with state structure	1980s	Skocpol 1980; Skocpol and Amenta 1986; Flora and Heidenheimer 1981	Mostly qualitative and typology
New institutionalist	State institutions shape policy outcomes	1990s	Skocpol 1992; Immergut 1992; Steinmo 1993; Thelen 1999; Tsebelis 1995	Veto players, federalism, electoral system, policy legacies
Role of right	Right parties introduce social policies	1990s-2000s	Van Kersbergen 2003; Van Kersbergen and Manow 2009; Jensen 2014	Christian democratic control, right vs hegemonic left, religion
New politics of welfare state	Constrained by existing structure and generosity	1990s-2000s	Pierson 1994; Pierson 2001	Policy legacies, electoral system
Left irrelevance and insider-outsider	Left no more generous; high EPL lowers insurance	2000s	Pontusson 1995; Boix 2000; Rueda 2007; Vlandas 2013	EPL, union density
Varieties of Capitalism	Type of capitalism shapes welfare state	2000s	Hall and Soskice 2001; Hancke et al. 2009, Estevez-Abe 2005; Mares 2000; Swensson 1991	Employer coordination, stock market presence, EPL
Globalisation and financialisation	Limits welfare state due to market liberalisation	1990s-2000s	Garrett 1998; Boix 2000; Rudra 2002; Rodrik 1998; Strange 1996; Swank and Betz 2003; Swank and Duane 2002	Trade and financial openness
Deindustrialisation and new service economy	Risks from deindustrialisation and new economy	1990s-2000s	Iversen and Wren 1998; Wren 2013	Share of services, knowledge economy

Note: The table is not exhaustive, and some authors and key variables may overlap in different strands.

Table S1.1.2. Variables and summary statistics

Statistic	N	Mean	St. Dev.	Min	Max
social expenditure	985	20.198	4.762	5.702	34.649
social expenditure (FD)	945	0.235	1.054	−2.900	16.251
social transfers	1,538	12.540	3.857	3.476	23.894
social transfers (FD)	1,497	0.105	0.832	−5.878	5.649
social rights	755	31.188	6.983	10.800	46.600
social rights (FD)	734	0.141	0.835	−2.900	10.200
index of corporatism	1,312	−0.00004	0.671	−1.149	1.810
crisis dummy	1,576	0.124	0.329	0.000	1.000
public debt index of disproportionality	1,643	5.812	4.833	0.307	26.764
share of elderly	1,551	13.563	2.935	5.727	25.058
EMU membership	1,650	0.157	0.364	0	1
employment protection legislation	724	2.163	0.861	0.257	5.000
GDP per capita	1,623	19,268.170	18,767.640	478.995	118,823.600
centrist cabinet posts	1,638	23.451	31.349	0.000	100.000
centrist parliamentarians	1,638	24.023	32.337	0.000	100.000
left-wing cabinet posts	1,638	32.378	36.337	0.000	100.000
left-wing parliamentarians	1,638	33.721	38.101	0.000	100.000
industrial employment	1,413	0.312	0.086	0.048	0.670
capital account openness	1,260	0.735	0.320	0.000	1.000
trade openness	1,578	80.355	53.101	8.930	374.148
constitutional structure	1,633	1.536	1.962	0.000	7.000
union density	1,332	40.151	20.012	6.531	99.069
unemployment rate	1,580	6.376	4.371	0.000	27.500

S1.2: Significance shares by model specifications

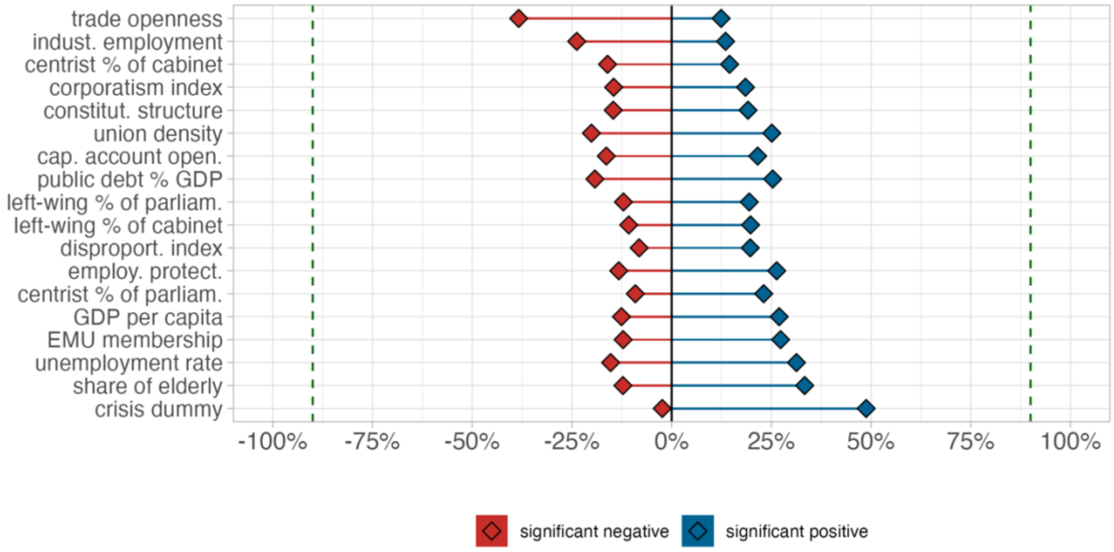

Figure S1.2.1. Significance shares of the independent variables. The figure plots the share of (positive and negative) significant coefficients (blue and red, respectively) of all independent variables in the full model space. A coefficient is classified as 'significant' if its p-value is below 0.1. The dashed line indicates 90%.

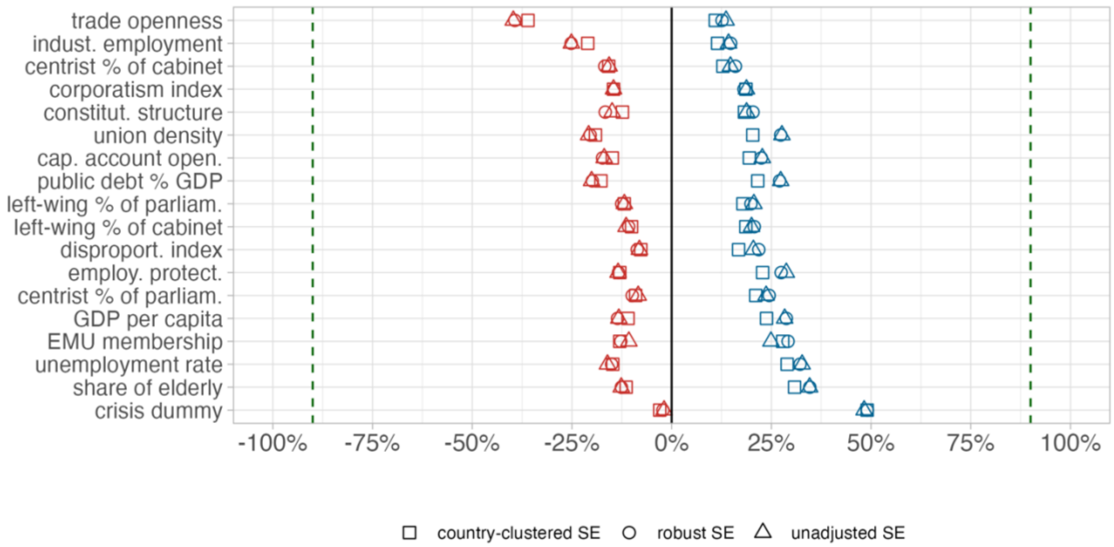

Figure S1.2.2. Significance shares of the independent variables by different standard error types. The figure plots the share of (positive and negative) significant coefficients (blue and red, respectively) of all independent variables by different standard error types in the full model space. A coefficient is classified as ‘significant’ if its p-value is below 0.1. The dashed line indicates 90%.

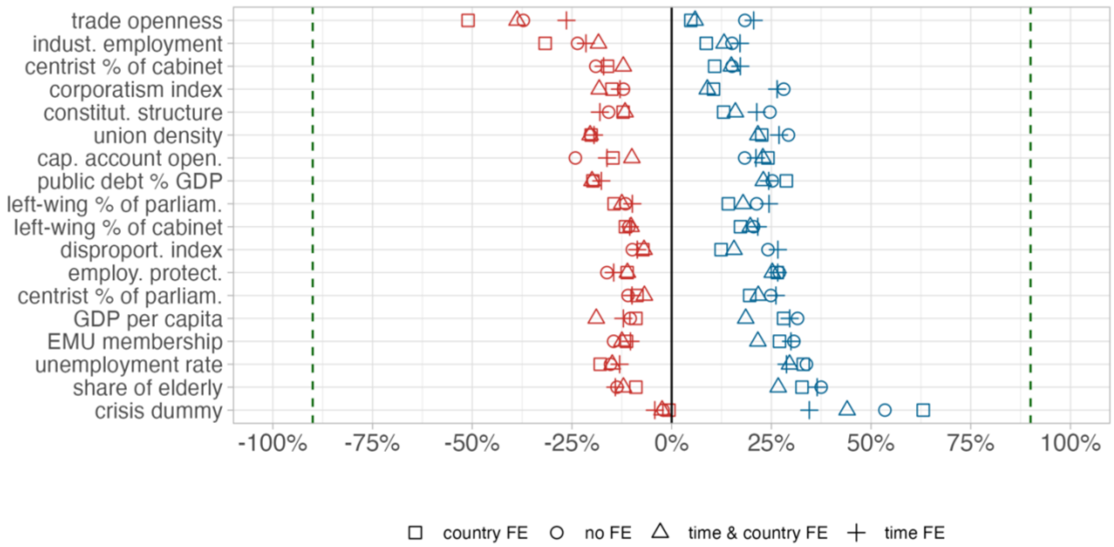

Figure S1.2.3. Significance shares of the independent variables by different fixed effect structures. The figure plots the share of (positive and negative) significant coefficients (blue and red, respectively) of all independent variables by different fixed effect structures in the full model space. A coefficient is classified as 'significant' if its p-value is below 0.1. The dashed line indicates 90%.

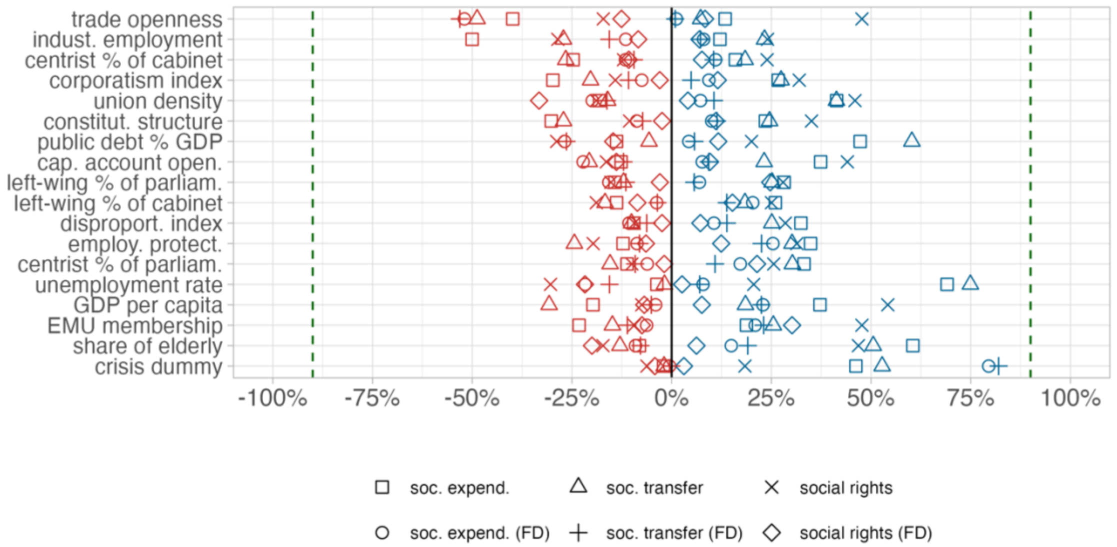

Figure S1.2.4. Significance shares of the independent variables by different dependent variables. The figure plots the share of (positive and negative) significant coefficients (blue and red, respectively) of all independent variables by different dependent variables in the full model space. A coefficient is classified as 'significant' if its p-value is below 0.1. The dashed line indicates 90%.

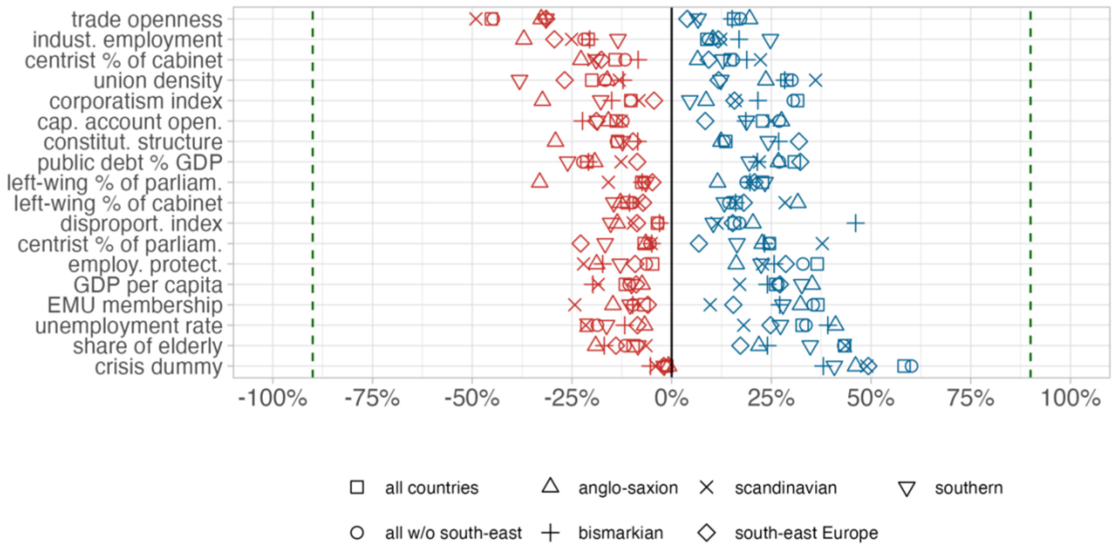

Figure S1.2.5. Significance shares of the independent variables by different country samples. The figure plots the share of (positive and negative) significant coefficients (blue and red, respectively) of all independent variables by different country samples in the full model space. A coefficient is classified as 'significant' if its p-value is below 0.1. The dashed line indicates 90%.

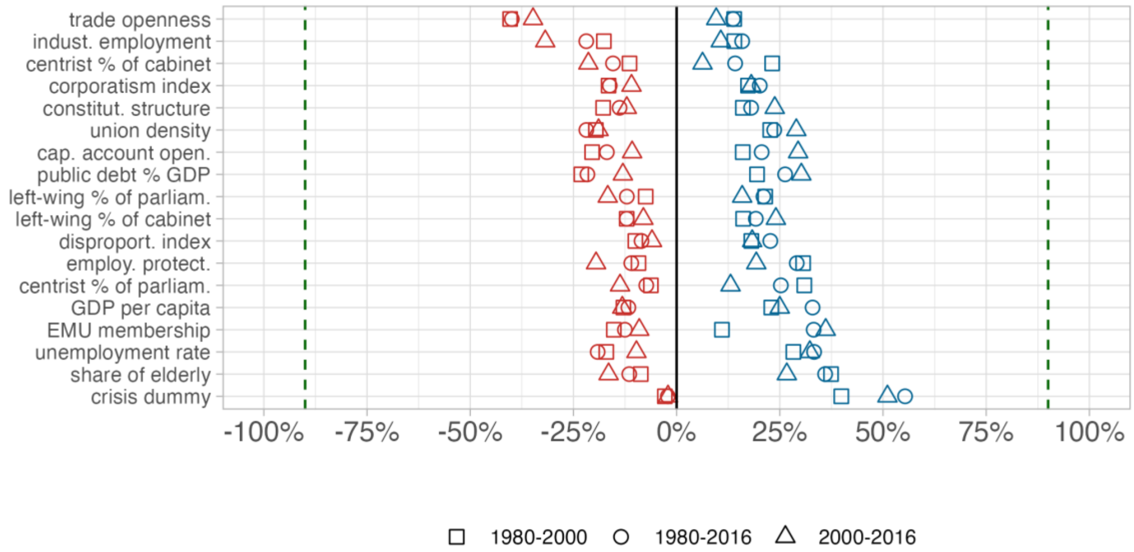

Figure S1.2.6. Significance shares of the independent variables by different period samples. The figure plots the share of (positive and negative) significant coefficients (blue and red, respectively) of all independent variables by different period samples in the full model space. A coefficient is classified as 'significant' if its p-value is below 0.1. The dashed line indicates 90%.

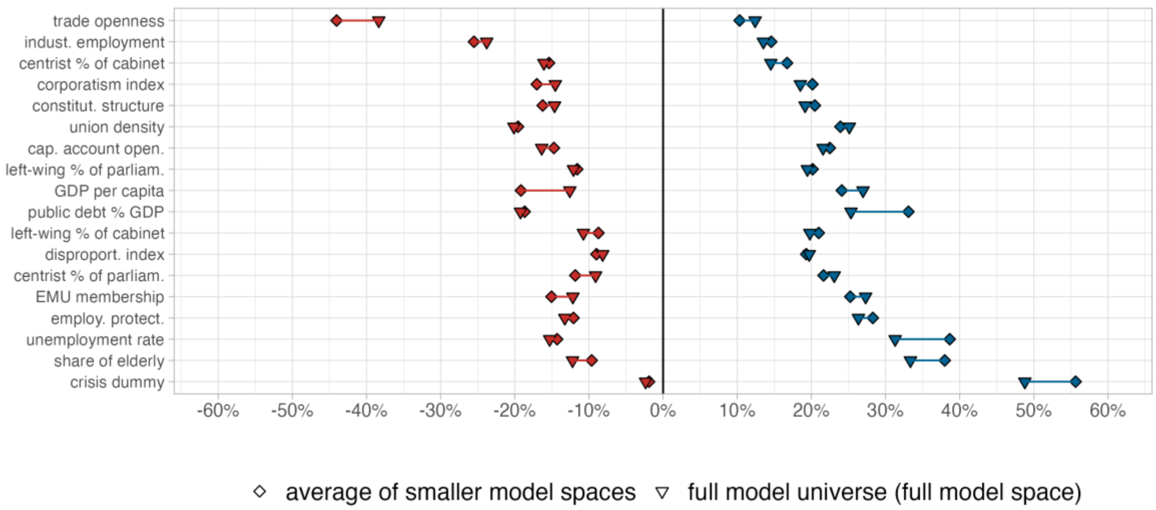

Figure S1.2.7. Significance shares of the independent variables across different model spaces. The figure shows the share of (positive and negative) significant coefficients (blue and red, respectively) for the full model spaces and the average significant shares of randomly-generated, smaller model spaces for all independent variables. The average significance shares of the small model spaces is based on 150 model spaces for each independent variable. The horizontal, connecting lines between the diamonds and triangles aims to highlight the (small) distance between the significance shares of the full model universe and the smaller model spaces.

S1.3: Significance shares of the restricted and weighted model space

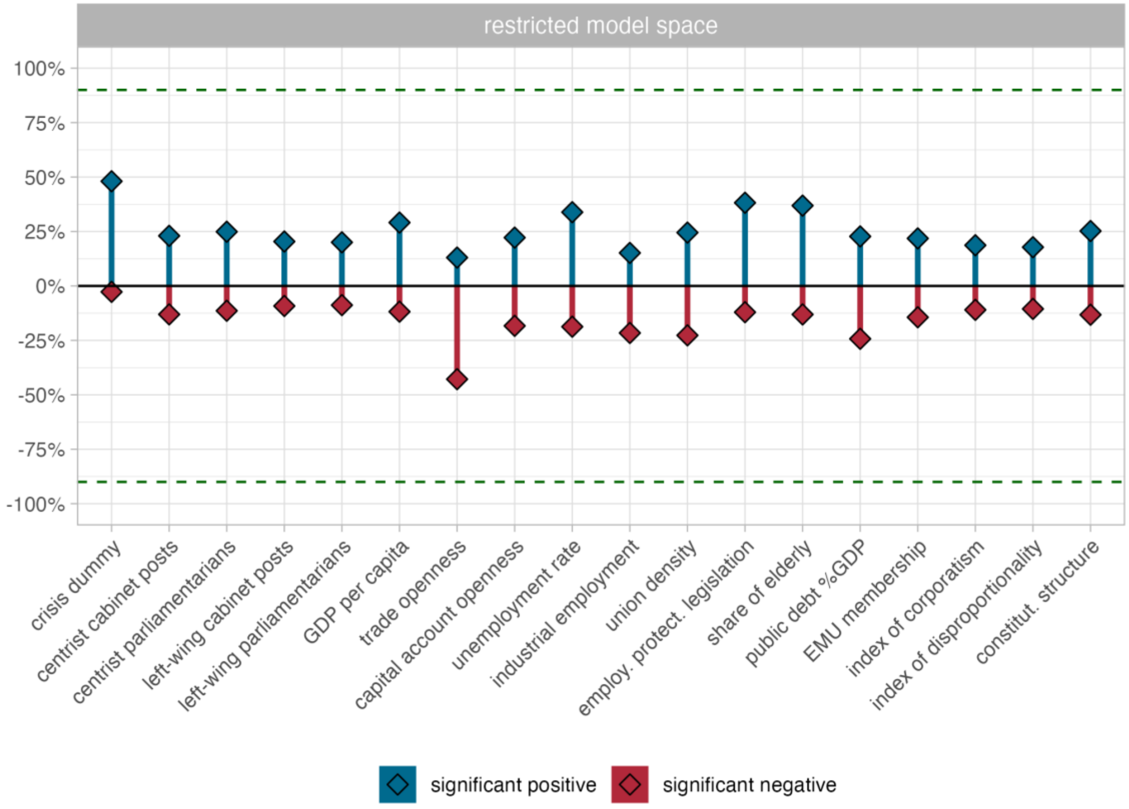

Figure S1.3.1. Significance shares of the independent variables in the restricted model space. The figure shows the share of (positive and negative) significant coefficients (blue and red, respectively) of all independent variables in the restricted model universe. The dashed line indicates 90%.

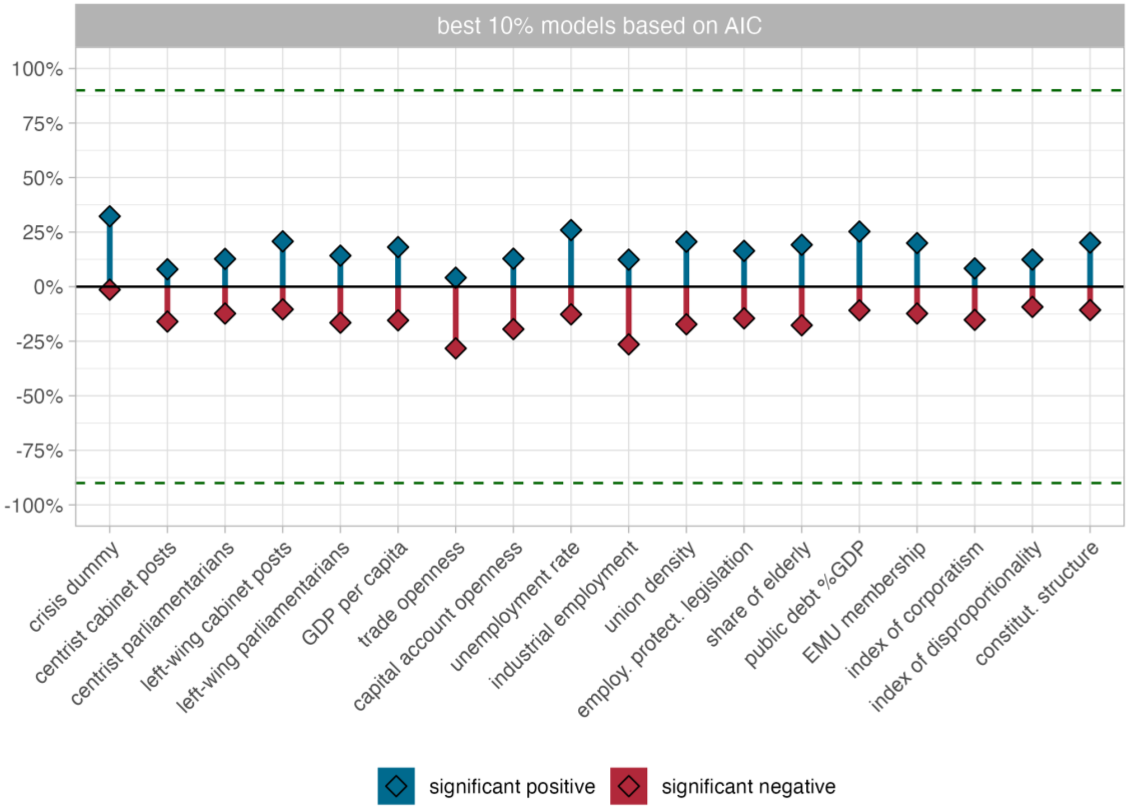

Figure S1.3.2. Significance shares of the independent variables in the model space with the 10% best-fitted models based on AIC. The figure shows the share of (positive and negative) significant coefficients (blue and red, respectively) of all independent variables in the restricted model universe which consists of models that indicate the 10% best-fitted models based on the Akaike Information Criterion (AIC). The dashed line indicates 90%.

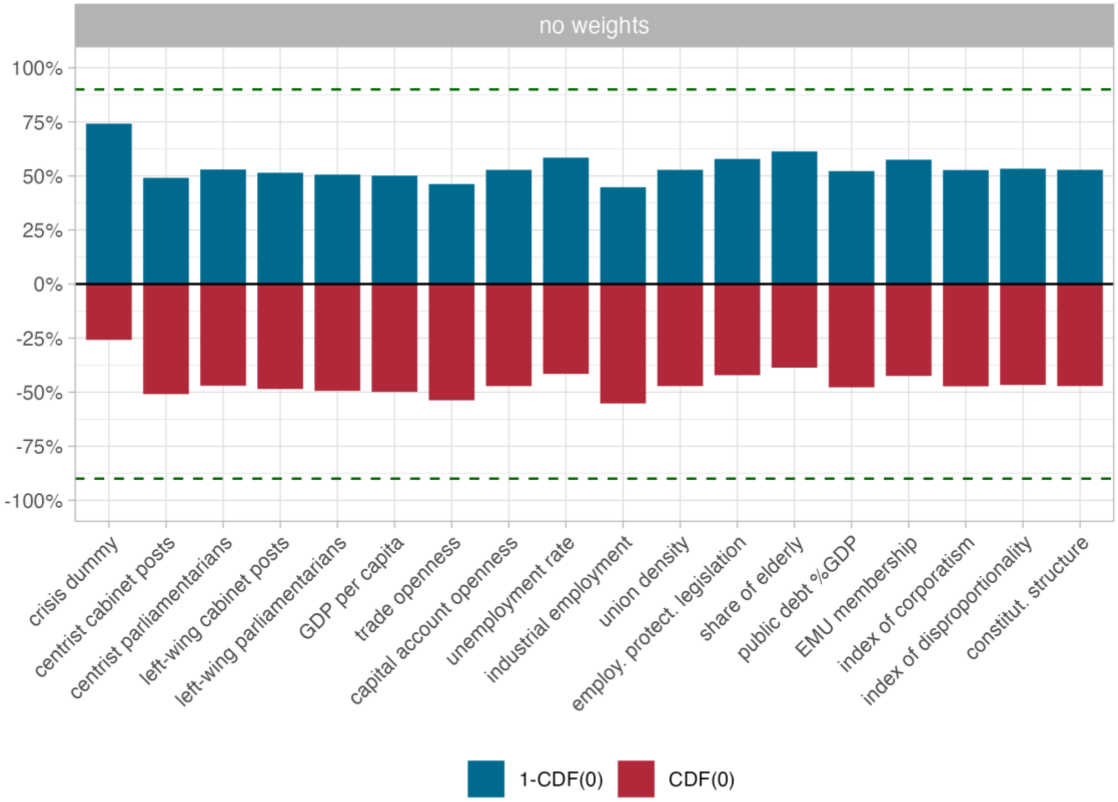

Figure S1.3.3. Cumulative density function (CDF) of the independent variables in the unrestricted model space without weights. The figure shows the cumulative distribution function (CDF) using the unrestricted model space. For each independent variable of interest, we construct the CDF without applying weights. If more than 90% (dashed vertical lines) of the probability mass of a variable is on either side of zero, this can be used as evidence that this determinant has significant uni-directional association with the outcome. The dashed line indicates 90%.

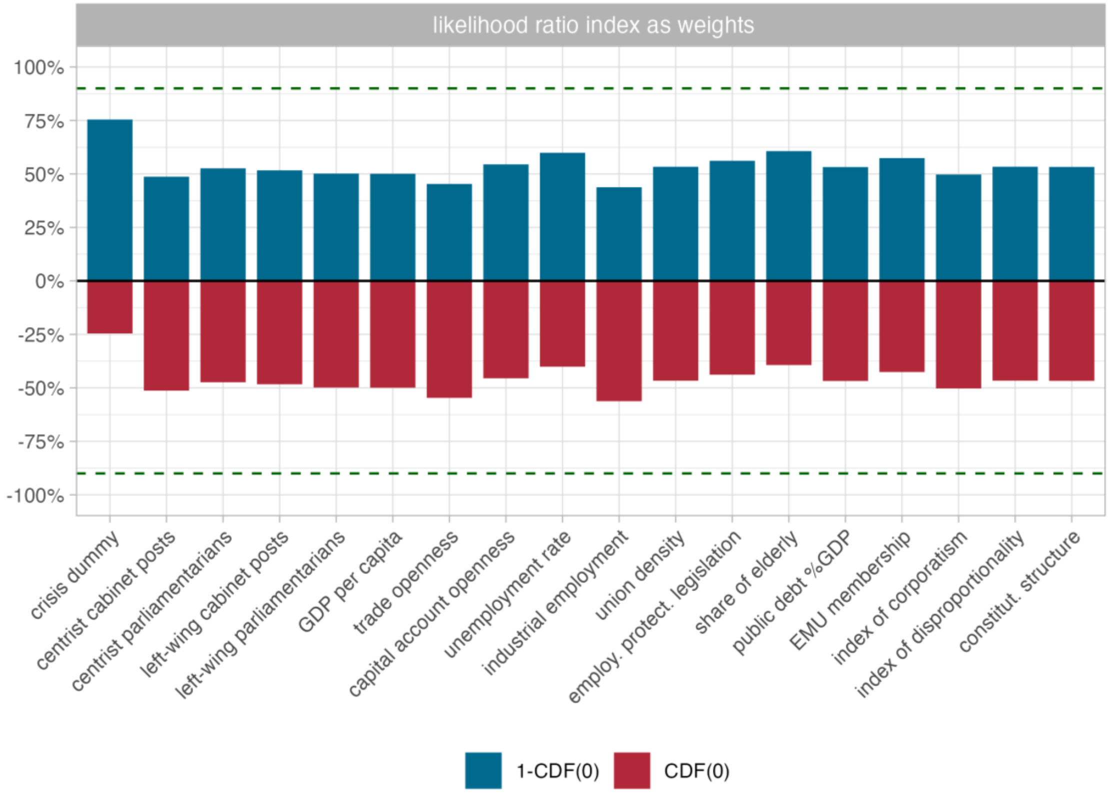

Figure S1.3.4. Cumulative density function (CDF) of the independent variables in the LRI-weighted model Space. The figure shows the cumulative distribution function (CDF) using the weighted model space. For each independent variable of interest, we construct the CDF using the likelihood ratio index as weights. If more than 90% (dashed vertical lines) of the probability mass of a variable is on either side of zero, this can be used as evidence that this determinant has significant uni-directional association with the outcome. The construction of the CDF using a goodness of fit measure as weights follows the approach adopted by Sala-i-Martin (1997). The dashed line indicates 90%.

S1.4: Impact of model specification on significance of estimates

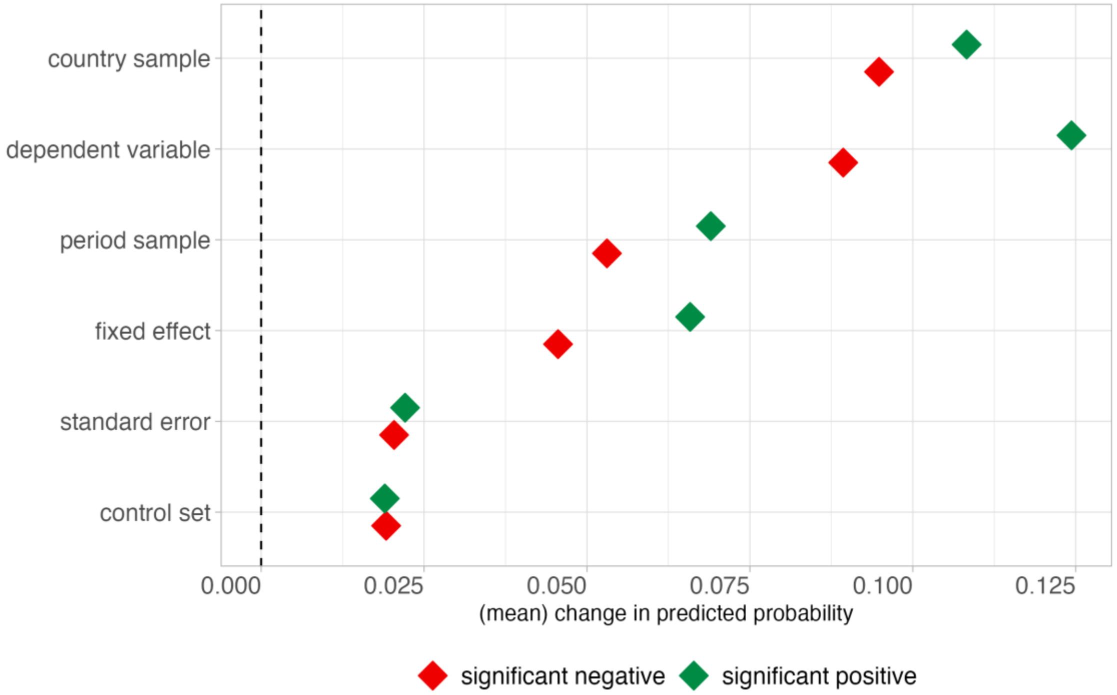

Figure S1.5.1. Average change in predicted probability for predicting the significance class after modifying one model specification. The figure displays the average change in predicted probabilities for six different model specification choices (y-axis), averaged across all independent variables. To estimate these average changes in predicted probabilities, we sampled a random set of 250,000 regression coefficients from the full model space and fit a multinomial logistic regression to predict whether an estimate is “negative significant,” “positive significant,” or “not significant” (significance class). We then calculated the average change in predicted probabilities by altering one model specification at a time to assess the impact of each specification choice, subsequently averaging across all independent variables.

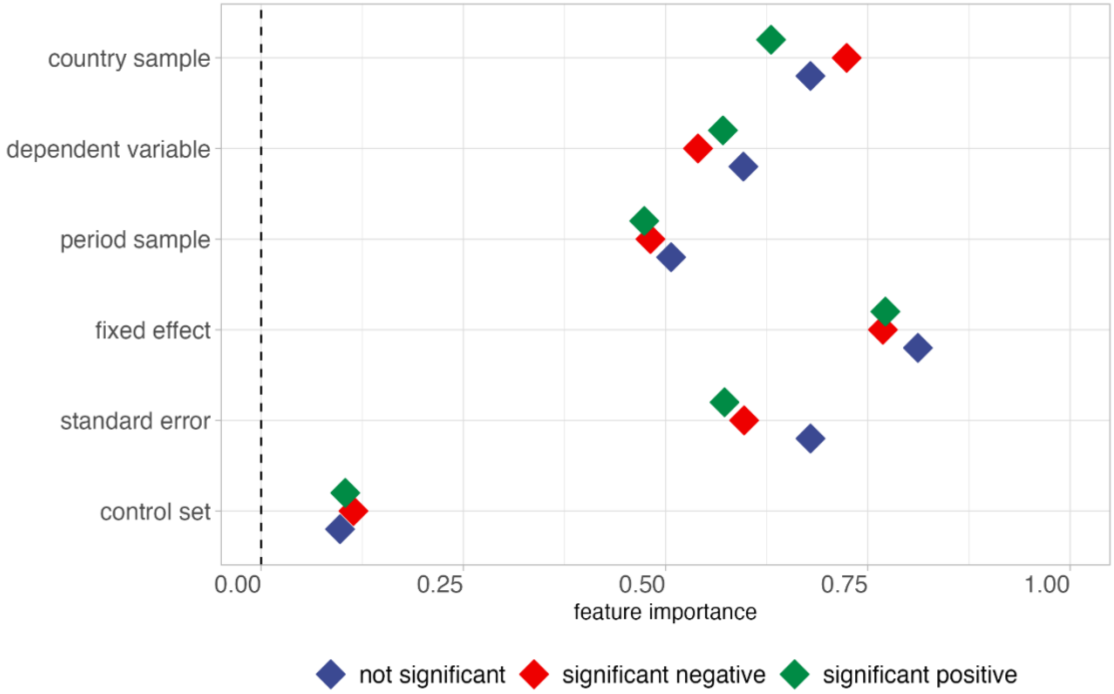

Figure S1.5.2. Feature importance scores (SHAP values) across all independent variables for the prediction of the significance class. The figure shows the average feature importance scores of six different model specification choices (x-axis) averaged across all independent variables under consideration. To estimate these scores, we extracted a random set of 250,000 regression coefficients from the full model space and then fit a deep learning model to build a classifier that predicts whether an estimate is “negative significant”, “positive significant” or “not significant”. Afterwards, we estimate the average SHAP values to assess the relevance of each model specification choice.

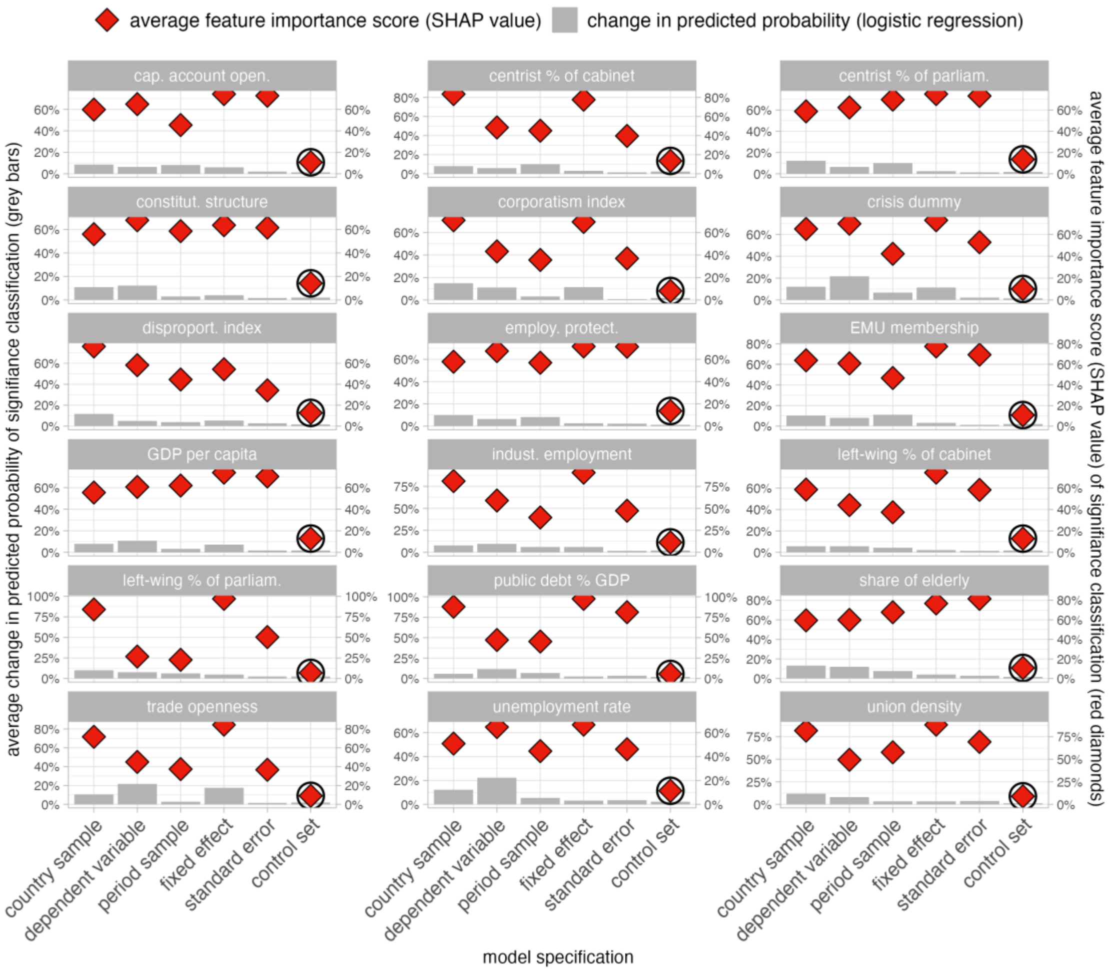

Figure S1.5.3. Average change in predicted probabilities and average feature importance scores (SHAP values) for each model specification binary in predicting the significance class. The figure presents the average change in predicted probabilities for six distinct model specification choices across the independent variables. To estimate these average changes in predicted probabilities (grey bars), we sampled a random set of 250,000 regression coefficients from the full model space and fit a multinomial logistic regression to predict whether an estimate is "negative significant," "positive significant," or "not significant" (significance class). We then calculated the average change in predicted probabilities by modifying one model specification at a time to assess the impact of each specification choice. For SHAP values (red diamonds), we used the same random sample of estimations as for the multinomial logistic regression. We applied a deep learning model to classify the significance class of each coefficient, then computed the average SHAP values to evaluate the importance of each model specification choice.

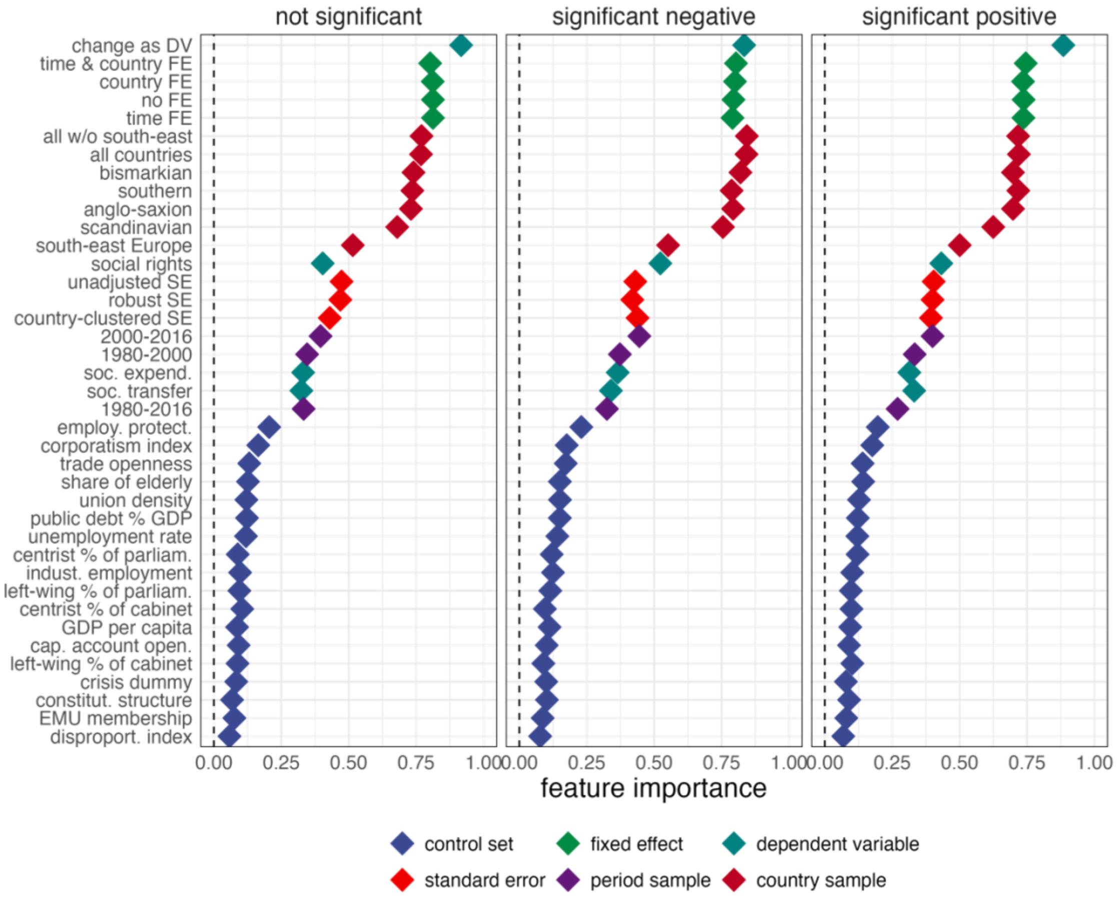

Figure S1.5.4. Feature importance scores (SHAP values) of specification binaries. The figure shows the average feature importance scores of all model specifications (x-axis) averaged across all independent variables under consideration. To estimate these scores, we extracted a random set of 250,000 regression coefficients from the full model space and then fit a deep learning model to build a classifier that predicts whether an estimate is “negative significant”, “positive significant” or “not significant”. Afterwards, we estimate the average SHAP values to assess the relevance of each model specification choice.

S2: Case 2: Democratization

S2.1: Literature review and data selection

In examining the determinants of democratization, we draw on the study by Claassen (2020), which provides a comprehensive analysis of various factors influencing democratic development. Democratization, a central topic in political science, involves the transition from non-democratic to democratic governance and the consolidation of democratic institutions. Numerous theoretical debates have emerged over the years, proposing different determinants of democracy, broadly categorized into economic, social, political, and institutional factors. We briefly discuss these factors in the next paragraphs.

First, economic development is often cited as a crucial driver of democratization. Key variables include the agricultural share of GDP, annual growth rate of GDP per capita, log GDP, and trade as a percentage of GDP (Acemoglu & Robinson, 2009). Higher levels of economic development, indicated by increased GDP and growth rates, are argued to create a more prosperous and educated middle class that demands greater political participation and accountability (Lipset, 1959). Conversely, a high agricultural share of GDP is often associated with less industrialization and slower democratic transitions. Additionally, economic openness, reflected in trade as a percentage of GDP, can expose countries to democratic norms and practices, fostering democratization (Milner, 1998).

Second, social composition and cultural values may also significantly correlate with democratization. Variables such as the percentage identifying as Muslim and the share of the population with secondary or tertiary education are critical in this debate (Inglehart, 1997). Higher educational attainment is often linked with greater political awareness and support for democratic norms (Barro, 1999). Cultural factors, including religion, can influence attitudes toward democracy; however, the relationship is complex and context-dependent (Huntington, 1993).

Third, political variables play a vital role in shaping democratic transitions. These include the Corruption Perceptions Index, support for democracy, satisfaction with democracy, and political institutions (Treisman, 2007). Lower levels of corruption are associated with higher trust in government and greater political stability, both of which are conducive to democratization (Sung, 2004). Public support for democracy and satisfaction with democratic processes are crucial for the legitimacy and sustainability of democratic institutions (Diamond, 1999).

Fourth, institutional quality and structural conditions also determine democratization. Variables such as the industrial share of employment, natural resources per capita, and log population are significant here (Ross, 2001). Industrial employment can foster democratization by creating a workforce that demands political representation (Huber et al., 1993). However, an abundance of natural resources, often referred to as the “resource curse,” can hinder democratization by enabling autocratic regimes to maintain power through resource revenues (Sachs & Warner, 2001). Population size and density can also impact democratization, influencing the complexity and scale of governance required (Ahlquist & Wibbels, 2010).

To explore these determinants of democratization, we use data from the study’s replication file available on Dataverse (Claassen, 2020) in addition to the World Development Indicator dataset (World Bank, 2020), but want to make clear that our purpose is not to carry out a replication study and our results should therefore not be interpreted in this way. These datasets comprise a cross-country panel covering nations worldwide, allowing us to carry out our analysis of the factors associated with democratic transitions. Specifically, we select 12 key independent variables to examine model uncertainty. A detailed description of all independent variables including summary statistics is provided in Table S2.1.1.

These variables encompass economic indicators, most notably agricultural share of GDP, annual growth rate of GDP per capita, log GDP, trade as a percentage of GDP, social and cultural factors such as the percentage identifying as Muslim, share with secondary or tertiary education (Barro, 1999; Inglehart, 1997), political measures including Corruption Perceptions Index, support for democracy, and satisfaction with democracy (Sung, 2004; Treisman, 2007), and structural factors, for example the industrial share of employment, natural resources per capita, log population (Ahlquist & Wibbels, 2010; Ross, 2001).

For our analysis, we define several country and period samples to account for regional and temporal variations. The country samples include all countries, Eastern Europe and Central Asia, Europe and North America, the Middle East and North Africa, South America, Sub-Saharan Africa, and East/South/Southeast Asia. The period samples are divided into four temporal groups: 1987-1997, 1998-2007, 2008-2018, and all periods.

Measuring democracy has been the focus of a longstanding methodological debate in political science. We use different democracy indicators, including the V-Dem Liberal Democracy Index (change), V-Dem Liberal Democracy Index (levels), V-Dem Electoral Democracy Index (levels), and V-Dem Regimes (ordinal scale) (Coppedge et al., 2011), which provide a comprehensive sets of variables measuring democratic transitions and consolidations across various contexts and time periods.

In addition to the control set, we apply different fixed effect structures, specifically using year fixed effects only, country fixed effects only, both country and year fixed effects, and no fixed effects. We also employ three types of standard errors: simple/unadjusted standard error, heteroscedasticity-robust standard error, and the Huber-White standard error clustered at the country level (Wooldridge, 2010).

With 12 independent variables, we create a control set for each combination of variables, resulting in a total of 4,095 control sets. Each of these sets is used in combination with seven country samples, four period samples, four dependent variables, four fixed effect structures, and three standard error types. Thus, in total, with 4,095 control sets and 1,344 model specifications (7 country samples × 4 period samples × 4 dependent variables × 4 fixed effect structures × 3 standard error types), we conduct 5,503,680 regressions in the entire model space. These regressions include approximately 33 million estimates (coefficient and standard error combinations).

Table S2.1.1. Variables and summary statistics

Statistic	N	Mean	St. Dev.	Min	Max
V-Dem Liberal Democracy index (levels)	4,170	44.130	27.205	1.221	90.342
V-Dem Electoral Democracy Index (levels)	4,185	55.460	26.613	1.504	93.998
V-Dem Regimes (ordinal scale)	4,185	1.712	1.036	0	3
V-Dem Liberal Democracy index (change)	4,169	0.582	4.314	−43.294	68.527
support for democracy	2,435	0.035	0.889	−2.108	2.719
natural resources per capita	3,810	0.064	0.244	0.000	1.000
percentage identifying as Muslim	4,185	22.633	34.610	0	100
satisfaction with democracy	2,549	−0.061	0.889	−2.841	2.451
log GDP	4,184	8.845	1.221	5.051	11.545
annual growth rate of GDP per capita	4,049	3.898	10.054	−88.139	255.182
Sign-log of inflation rate	4,042	1.688	1.501	−4.791	13.902
corruption perceptions index	2,452	59.437	22.045	4.000	100.000
(log) population	1,953	16.410	1.753	12.650	21.057
share with sec. or tert. education	1,368	78.531	28.684	5.850	164.080
trade as percentage of GDP	1,806	73.038	45.600	0.021	353.794
industrial share of employment	1,761	28.123	10.346	3.243	84.796
agricultural share of GDP	1,734	12.609	11.471	0.214	79.042

S2.2: Significance shares by model specifications

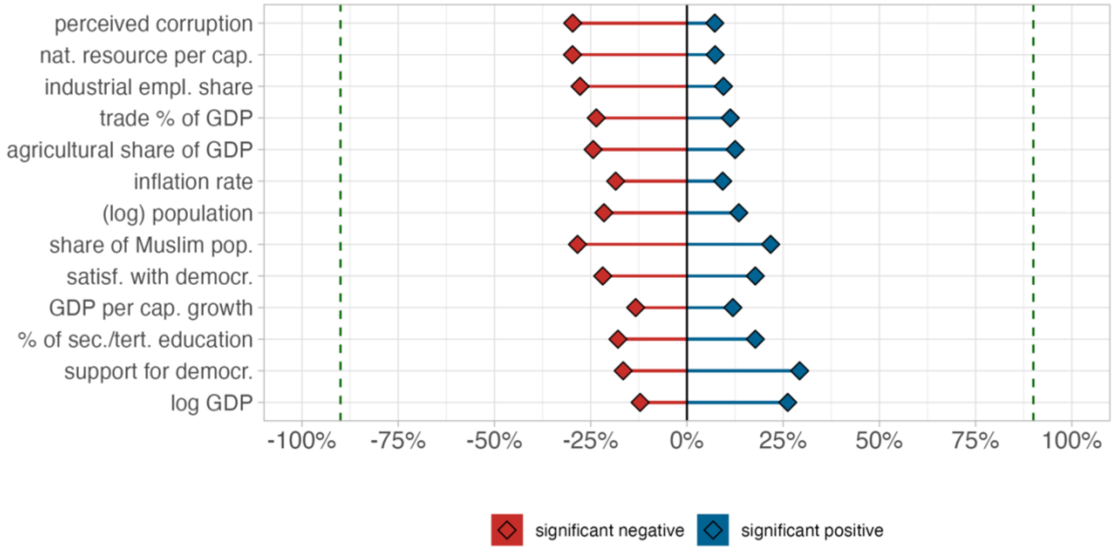

Figure S2.2.1. Significance shares of the independent variables. The figure plots the share of (positive and negative) significant coefficients (blue and red, respectively) of all independent variables in the full model space. A coefficient is classified as ‘significant’ if its p-value is below 0.1. The dashed line indicates 90%.

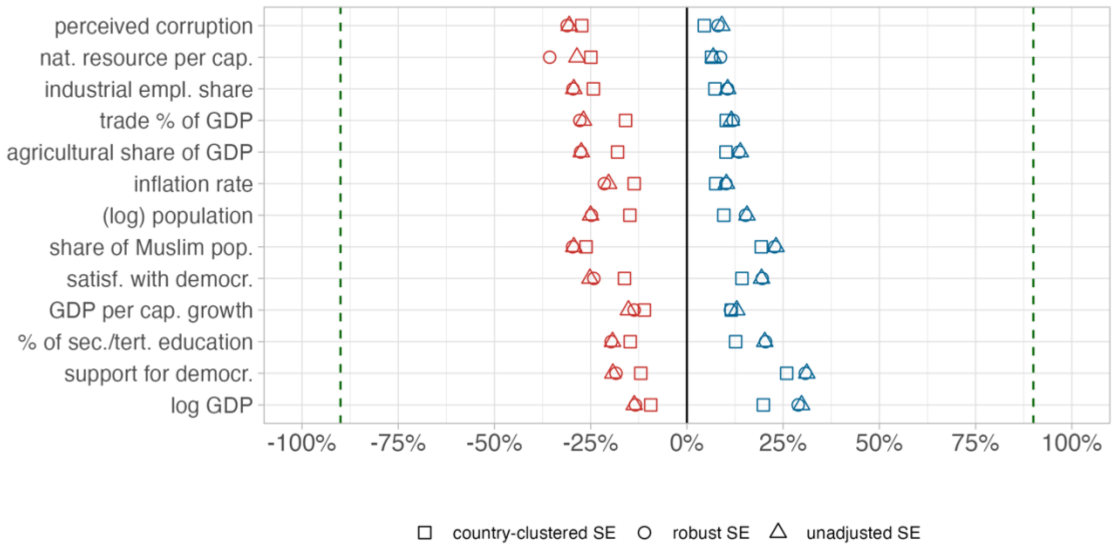

Figure S2.2.2. Significance shares of the independent variables by different standard error types. The figure plots the share of (positive and negative) significant coefficients (blue and red, respectively) of all independent variables by different standard error types in the full model space. A coefficient is classified as 'significant' if its p-value is below 0.1. The dashed line indicates 90%.

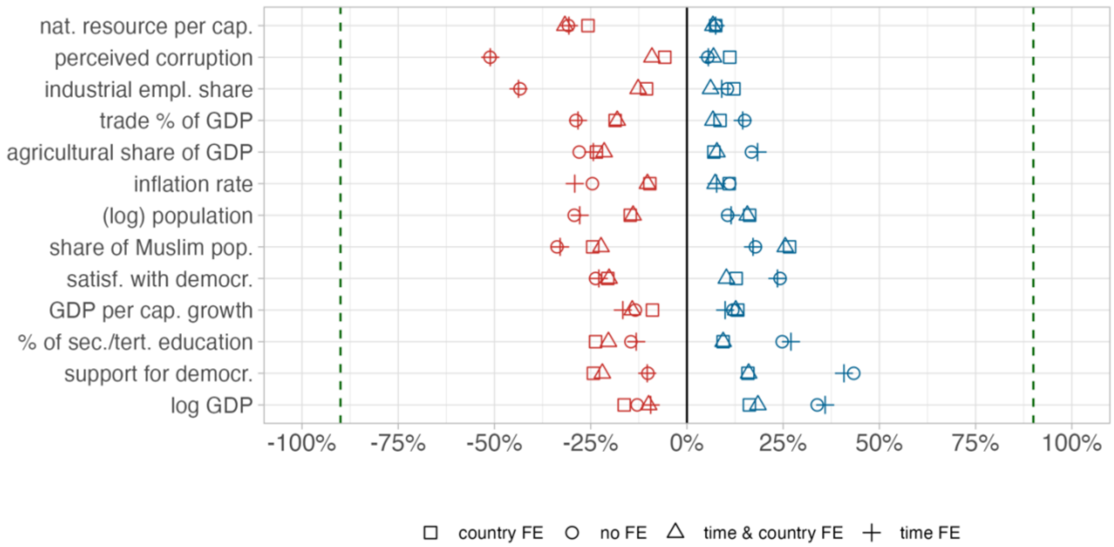

Figure S2.2.3. Significance shares of the independent variables by different fixed effect structures. The figure plots the share of (positive and negative) significant coefficients (blue and red, respectively) of all independent variables by different fixed effect structures in the full model space. A coefficient is classified as 'significant' if its p-value is below 0.1. The dashed line indicates 90%.

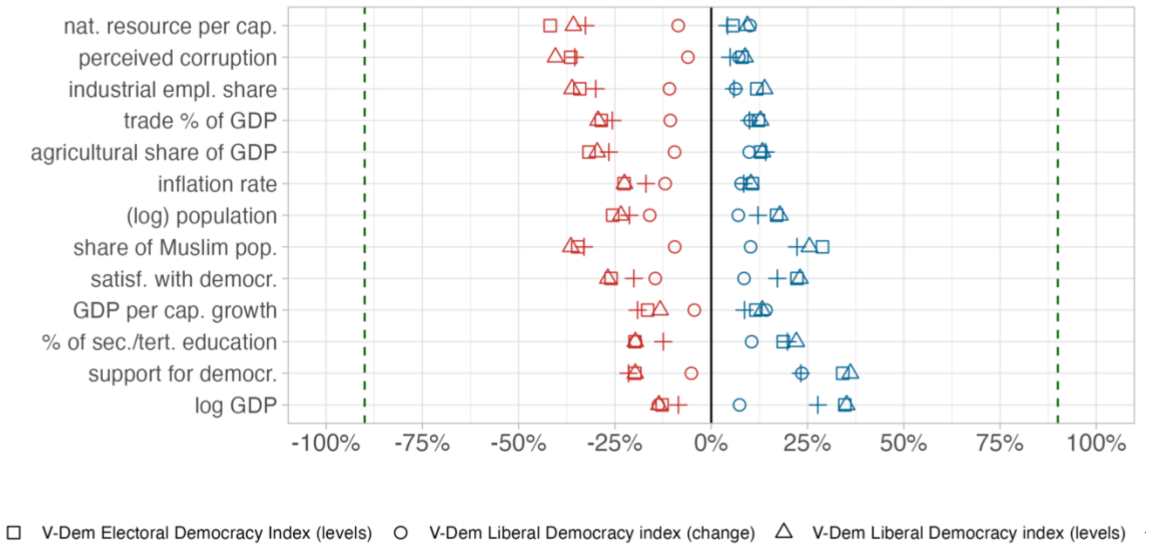

Figure S2.2.4. Significance shares of the independent variables by different dependent variables. The figure plots the share of (positive and negative) significant coefficients (blue and red, respectively) of all independent variables by different dependent variables in the full model space. A coefficient is classified as 'significant' if its p-value is below 0.1. The dashed line indicates 90%.

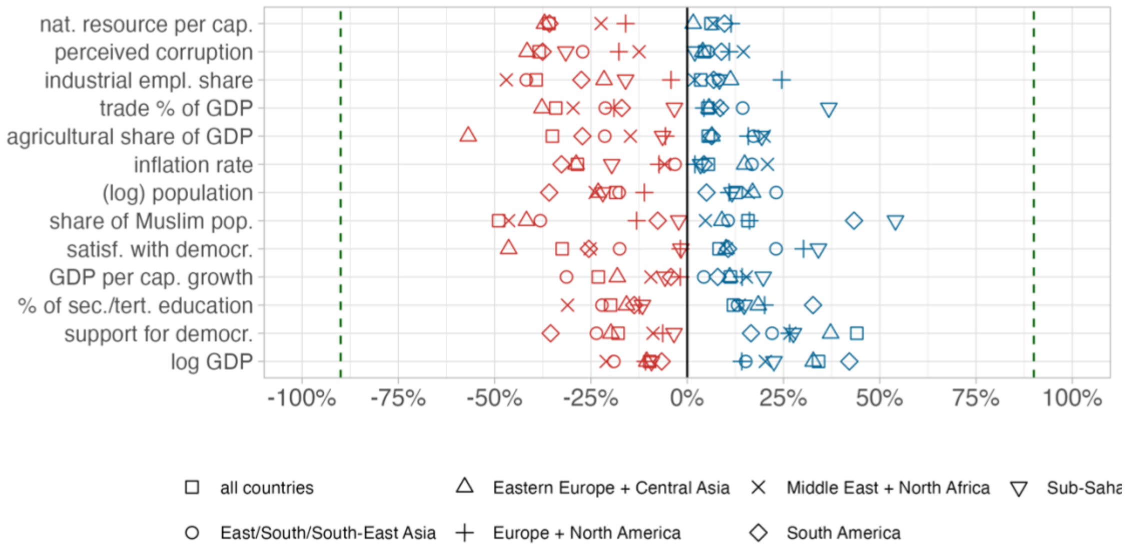

Figure S2.2.5. Significance shares of the independent variables by different country samples. The figure plots the share of (positive and negative) significant coefficients (blue and red, respectively) of all independent variables by different country samples in the full model space. A coefficient is classified as 'significant' if its p-value is below 0.1. The dashed line indicates 90%.

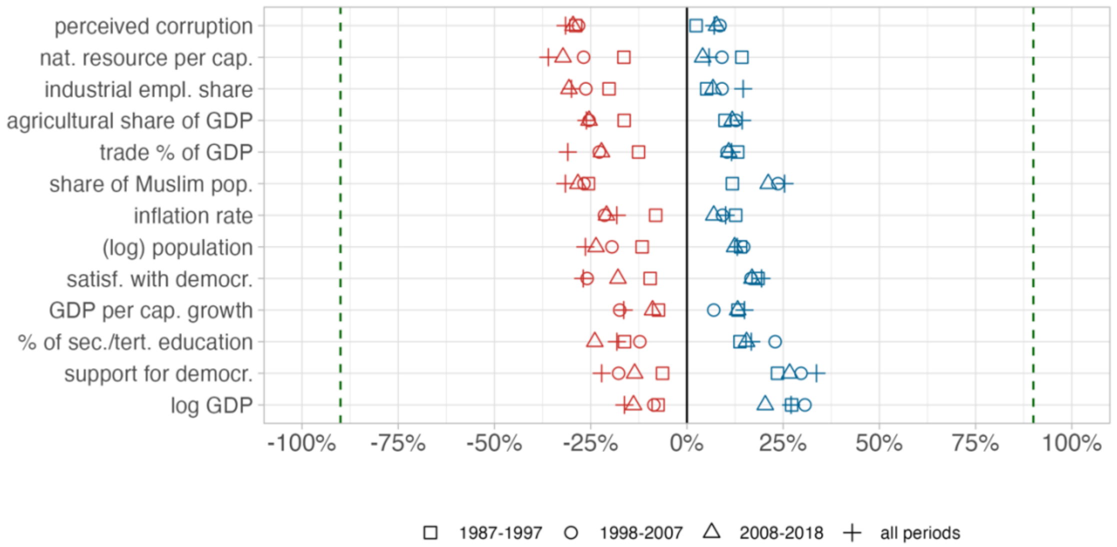

Figure S2.2.6. Significance shares of the independent variables by different period samples. The figure plots the share of (positive and negative) significant coefficients (blue and red, respectively) of all independent variables by different period samples in the full model space. A coefficient is classified as 'significant' if its p-value is below 0.1. The dashed line indicates 90%.

S2.3: Impact of model specification on significance of estimates

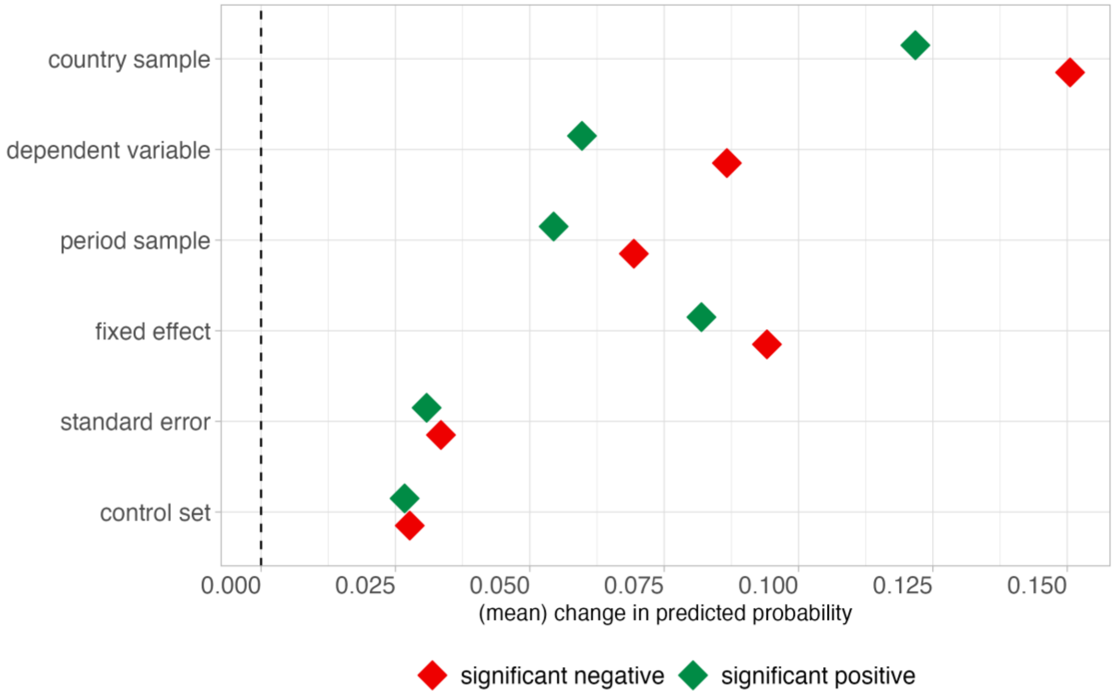

Figure S2.3.1. Average change in predicted probability for predicting the significance class after modifying one model specification. The figure displays the average change in predicted probabilities for six different model specification choices (y-axis), averaged across all independent variables. To estimate these average changes in predicted probabilities, we sampled a random set of 250,000 regression coefficients from the full model space and fit a multinomial logistic regression to predict whether an estimate is "negative significant," "positive significant," or "not significant" (significance class). We then calculated the average change in predicted probabilities by altering one model specification at a time to assess the impact of each specification choice, subsequently averaging across all independent variables.

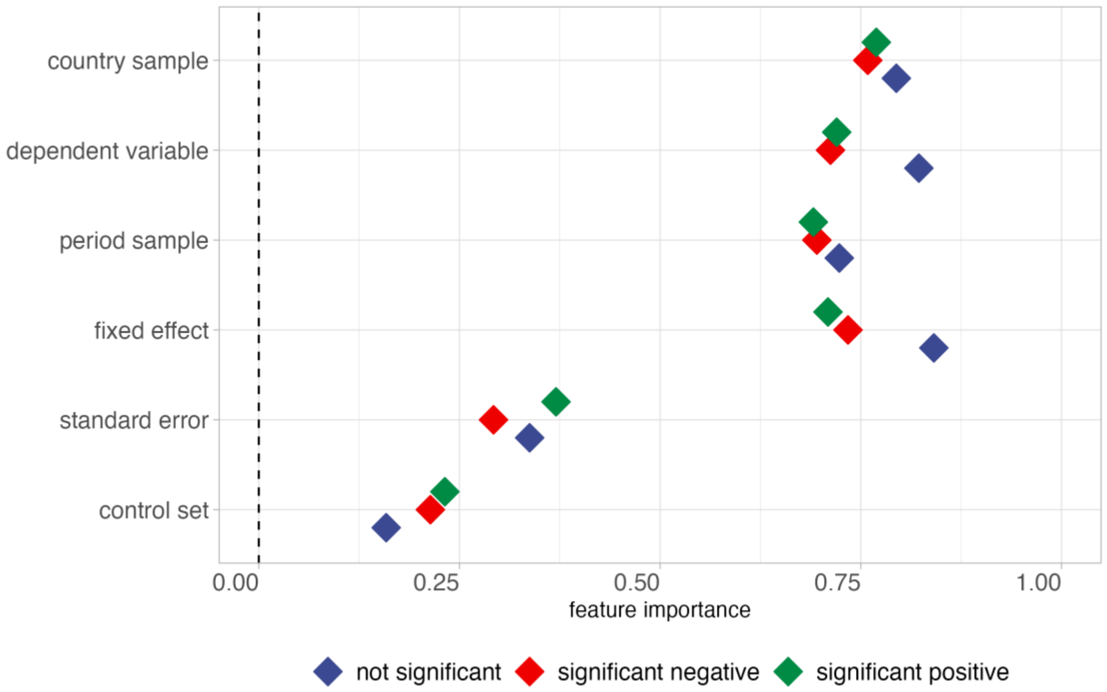

Figure S2.3.2. Average change in feature importance scores (SHAP values) across all independent variables for the prediction of the significance class. The figure shows the average change in predicted probabilities of six different model specification choices (x-axis) averaged across all independent variables under consideration. To estimate these averages changes in predicted probabilities, we extracted a random set of 250,000 regression coefficients from the full model space and then fit a deep learning model to build a classifier that predicts whether an estimate is “negative significant”, “positive significant” or “not significant”. Afterwards, we estimate the average SHAP values to assess the relevance of each model specification choice.

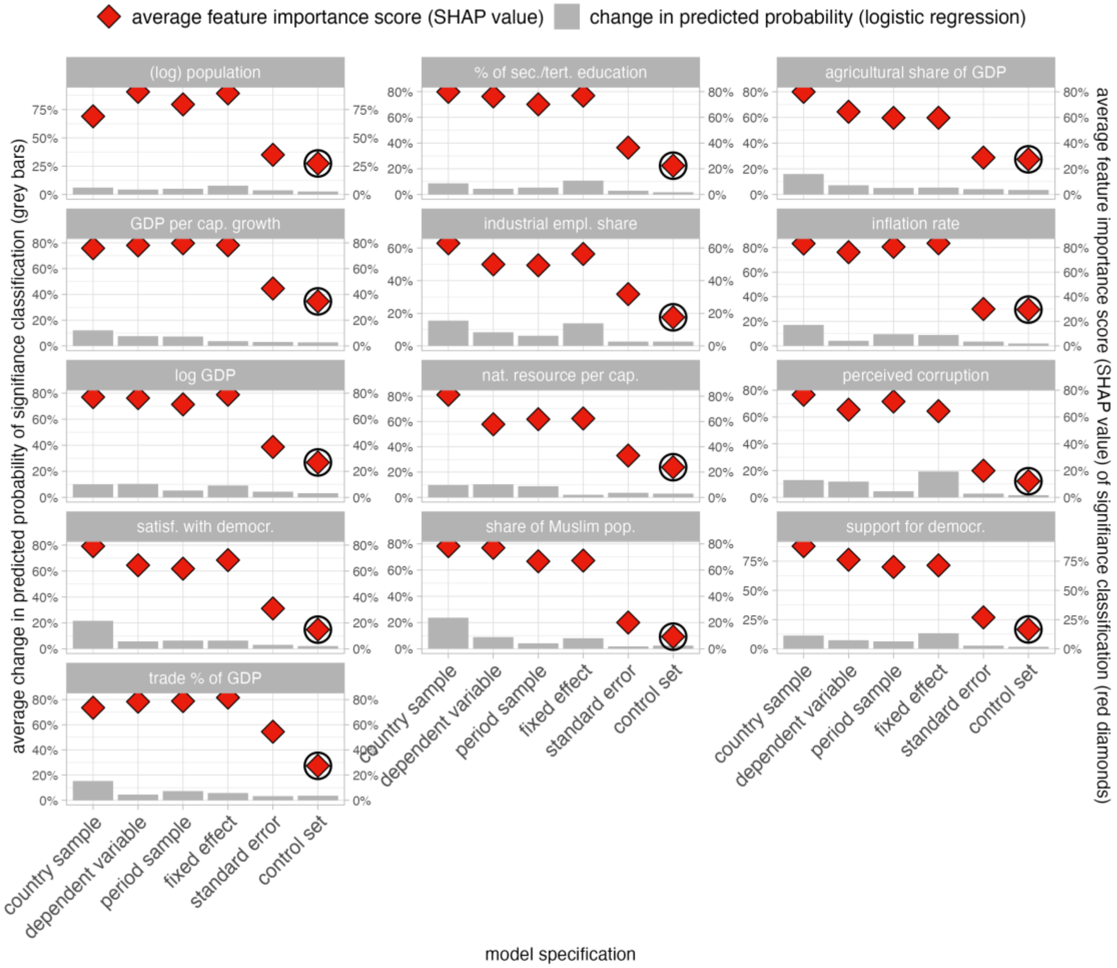

Figure S2.3.3. Average change in predicted probabilities and average feature importance scores (SHAP values) for each model specification binary in predicting the significance class. The figure presents the average change in predicted probabilities for six distinct model specification choices across the independent variables. To estimate these average changes in predicted probabilities (grey bars), we sampled a random set of 250,000 regression coefficients from the full model space and fit a multinomial logistic regression to predict whether an estimate is “negative significant,” “positive significant,” or “not significant” (significance class). We then calculated the average change in predicted probabilities by modifying one model specification at a time to assess the impact of each specification choice. For SHAP values (red diamonds), we used the same random sample of estimations as for the multinomial logistic regression. We applied a deep learning model to classify the significance class of each coefficient, then computed the average SHAP values to evaluate the importance of each model specification choice.

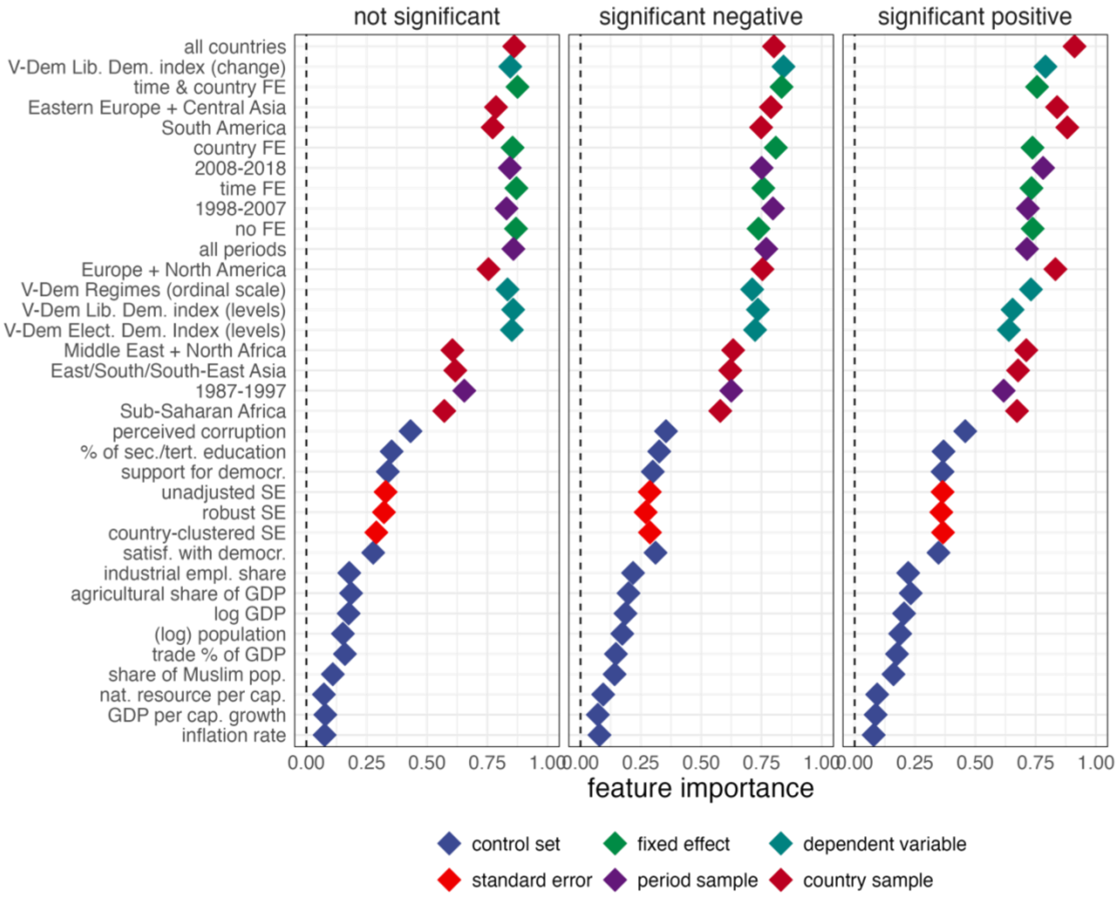

Figure S2.3.4. Feature importance scores (SHAP values) of specification binaries. The figure shows the average feature importance scores of all model specifications (x-axis) averaged across all independent variables under consideration. To estimate these scores, we extracted a random set of 250,000 regression coefficients from the full model space and then fit a deep learning model to build a classifier that predicts whether an estimate is “negative significant”, “positive significant” or “not significant”. Afterwards, we estimate the average SHAP values to assess the relevance of each model specification choice.

S3: Case 3: Regional Public Good Provision

S3.1: Literature review and data selection

In examining the determinants of regional public good provision, we draw upon a landmark article by Hong (2018), who carried out a comprehensive analysis of various factors influencing the provision of public services in China. The provision of public goods, such as education and healthcare, is critical for social welfare and development. Numerous theoretical debates have emerged over the years, proposing different determinants of public good provision, which can be broadly categorized into economic, political, and demographic factors.

First, economic conditions are fundamental in shaping the capacity and willingness of governments to provide public goods. Key variables in this context include GDP, coal, gas, and oil sales, foreign direct investment (FDI) in manufacturing, and the share of state-owned enterprises (SOEs) in production (Acemoglu et al., 2001; Barro, 1991). Higher levels of GDP indicate greater fiscal capacity to invest in public services. Revenues from natural resources, such as coal, gas, and oil sales, can provide substantial financial resources for public good provision. However, reliance on these revenues may also lead to inefficiencies and corruption, potentially undermining public service delivery (Sachs & Warner, 1995). FDI in manufacturing can spur economic growth and increase government revenues, thereby enabling greater investment in public goods (Borensztein et al., 1998). The dominance of SOEs can also impact public service provision, as these enterprises are often involved in sectors critical to public welfare.

Second, political variables play a significant role in determining the provision of public goods. The relationship between natural resources and public good provision is a central focus of Hong's (2018) study. It is argued that authoritarian leaders, especially in resource-rich regions, may strategically allocate resources to public goods to maintain political stability and legitimacy. This aligns with the broader theoretical debate on the "resource curse," where resource wealth can either foster public welfare through enhanced service provision or hinder it through corruption and mismanagement (Ross, 2001).

Third, the demographic characteristics of a region, such as population size, urbanization, and education levels, significantly impact the demand and supply of public goods. Variables such as population growth, urban population percentage, number of primary students, and land area are critical in this debate (Bloom et al., 2004; Easterly & Levine, 2003). Rapid population growth and urbanization increase the demand for public services, while regions with larger populations may benefit from economies of scale in public good provision. Education levels, indicated by the number of primary students, also influence public service demand and provision, as more educated populations typically demand higher quality services (Glaeser et al., 2004).

To test the relevance of these empirical choices, we use the data from the study's replication file available on Dataverse (Hong, 2018), but want to make clear that our purpose is not to carry out a replication study and our results should therefore not be interpreted in this way. This dataset is a cross-province panel covering the entirety of China, providing a broad and comprehensive analysis of public good provision across different regions. Based on Hong's study, we select 11 key independent variables to investigate model uncertainty. A detailed description of all independent variables, including summary statistics and sources, is provided in Table S3.1.1. These variables encompass economic indicators (coal sales, gas sales, oil sales, share of SOE production, GDP, FDI in manufacturing), demographic factors (population increase, population size, urban population percentage, number of primary students, land area), and political measures.

With 11 independent variables, we create a control set for each combination of variables, resulting in a total of 2,047 control sets. Each of these sets is used in combination with seven

regional samples, three period samples, nine dependent variables, four fixed effect structures, and three standard error types. Thus, in total, with 2,047 control sets and 2,268 model specifications (7 regional samples × 3 period samples × 9 dependent variables × 4 fixed effect structures × 3 standard error types), we conduct 4,642,596 regressions in the entire model space. These regressions include approximately 25.5 million estimates (coefficient and standard error combinations).

For our analysis, we define several regional and temporal samples to account for spatial and temporal variations. The regional samples include all regions, Central China, East China, North China, North-East China, North-West China, South China, and South-West China. The period samples are divided into three temporal groups: 1992-1999, 2000-2010, and all periods. The provision of public goods is measured using various indicators, including the number of hospital beds, doctors, hospitals, primary schools, secondary schools, tertiary schools, and the number of teachers at each educational level. These indicators provide a comprehensive view of public good provision across different contexts and time periods. Again, we use four different fixed effect structures: year fixed effects only, region fixed effects only, both region and year fixed effects, and no fixed effects. Additionally, we apply three types of standard errors: simple/unadjusted standard error, heteroscedasticity-robust standard error, and the Huber-White standard error clustered at the regional level.

Table S3.1.1. Variable and summary statistics

Variable	N	Mean	St. Dev.	Min	Max
(log) primary schools	4,063	6.826	0.937	2.079	9.697
(log) secondary schools	4,063	5.302	0.698	2.303	7.383
(log) tertiary schools	3,822	1.448	0.866	0.000	4.489
(log) primary teachers	4,622	9.526	0.762	6.410	11.704
(log) secondary teachers	4,622	9.304	0.774	5.043	11.576
(log) tertiary teachers	4,279	6.760	1.741	0.000	10.953
(log) hospitals	4,807	5.025	0.840	0.693	7.861
(log) hospital beds	4,807	8.981	0.758	6.412	11.493
(log) doctors	4,806	8.452	0.758	5.932	11.054
(lag) oil sales	5,452	0.172	1.028	0.000	14.405
(lag) gas sales	5,452	0.472	3.403	0.000	118.490
(lag) coal sales	5,452	0.275	0.798	0.000	15.795
(log) primary students	4,873	3.339	0.789	0.293	5.668
(log) GDP	4,612	14.900	1.181	10.561	18.829
(log) population	5,063	5.674	0.833	2.392	8.094
(log) land area	4,809	9.162	0.934	4.431	12.443
FDI in manufacturing	2,849	15.981	2.470	6.562	22.922
5y nat. pop. increase	3,429	5.728	2.865	−2.544	20.580

S3.2: Significance shares by model specifications

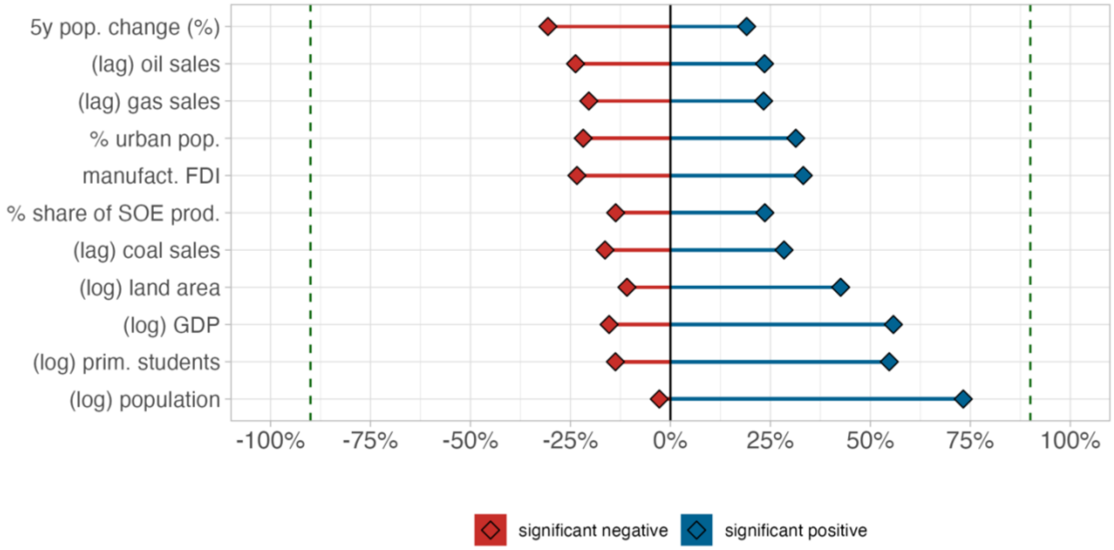

Figure S3.2.1. Significance shares of the independent variables. The figure plots the share of (positive and negative) significant coefficients (blue and red, respectively) of all independent variables in the full model space. A coefficient is classified as 'significant' if its p-value is below 0.1. The dashed line indicates 90%.

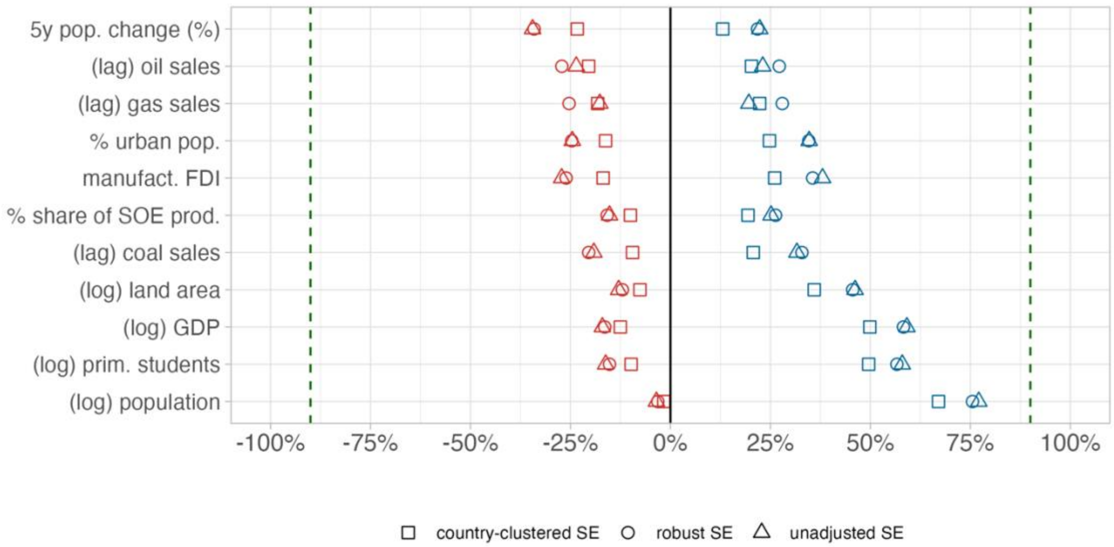

Figure S3.2.2. Significance shares of the independent variables by different standard error types. The figure plots the share of (positive and negative) significant coefficients (blue and red, respectively) of all independent variables by different standard error types in the full model space. A coefficient is classified as 'significant' if its p-value is below 0.1. The dashed line indicates 90%.

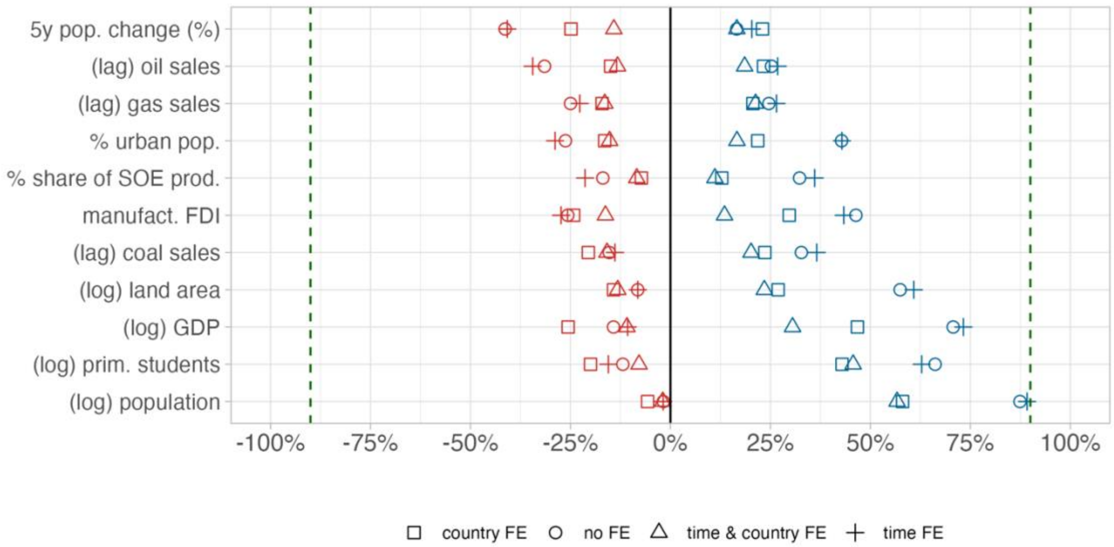

Figure S3.2.3. Significance shares of the independent variables by different fixed effect structures. The figure plots the share of (positive and negative) significant coefficients (blue and red, respectively) of all independent variables by different fixed effect structures in the full model space. A coefficient is classified as 'significant' if its p-value is below 0.1. The dashed line indicates 90%.

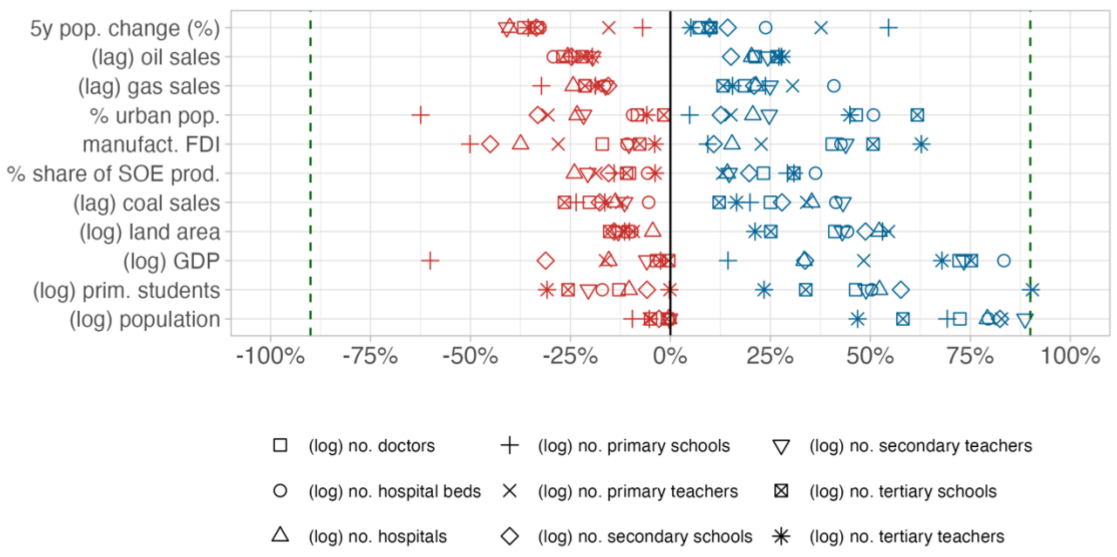

Figure S3.2.4. Significance shares of the independent variables by different dependent variables. The figure plots the share of (positive and negative) significant coefficients (blue and red, respectively) of all independent variables by different dependent variables in the full model space. A coefficient is classified as 'significant' if its p-value is below 0.1. The dashed line indicates 90%.

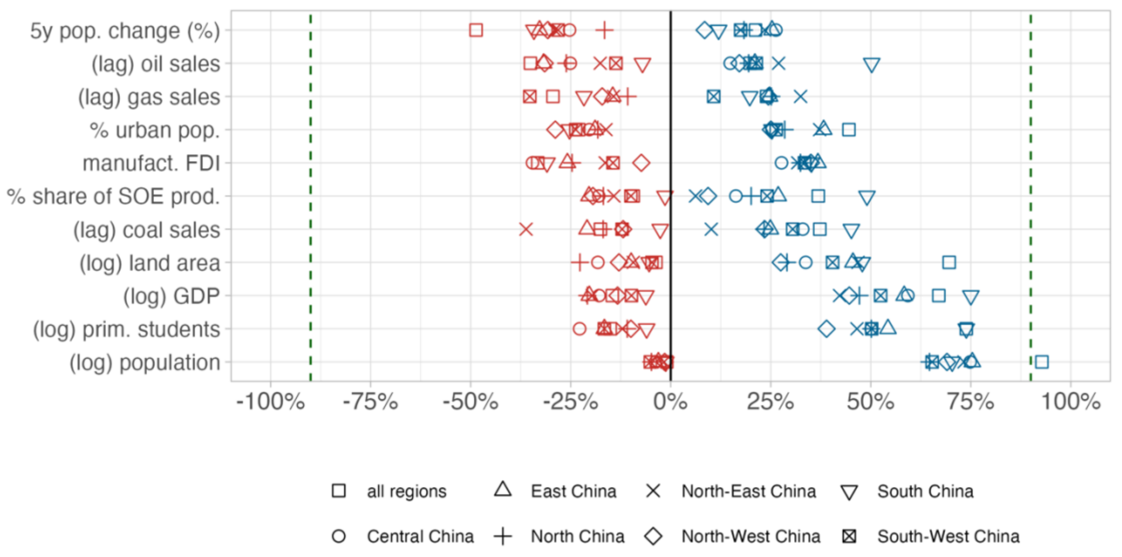

Figure S3.2.5. Significance shares of the independent variables by different country samples. The figure plots the share of (positive and negative) significant coefficients (blue and red, respectively) of all independent variables by different country samples in the full model space. A coefficient is classified as 'significant' if its p-value is below 0.1. The dashed line indicates 90%.

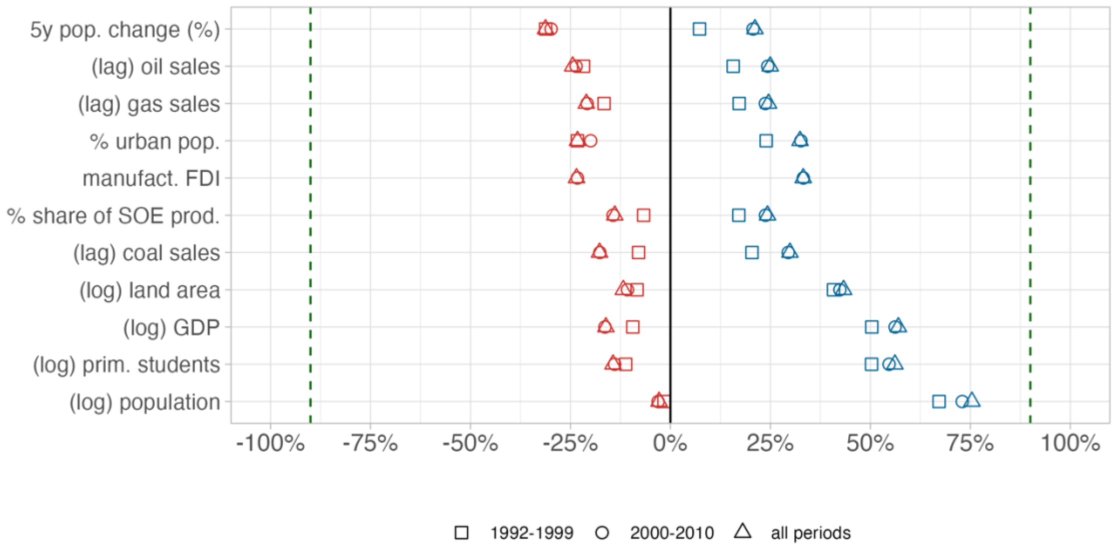

Figure S3.2.6. Significance shares of the independent variables by different period samples. The figure plots the share of (positive and negative) significant coefficients (blue and red, respectively) of all independent variables by different period samples in the full model space. A coefficient is classified as 'significant' if its p-value is below 0.1. The dashed line indicates 90%.

S3.3: Impact of model specification on significance of estimates

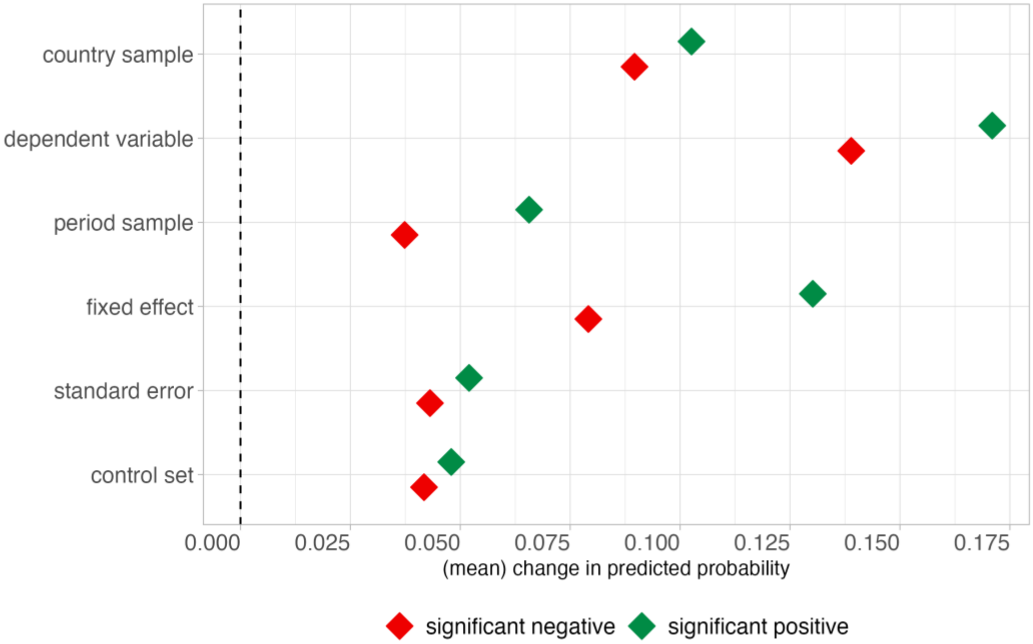

Figure S3.3.1. Average change in predicted probability for predicting the significance class after modifying one model specification. The figure displays the average change in predicted probabilities for six different model specification choices (y-axis), averaged across all independent variables. To estimate these average changes in predicted probabilities, we sampled a random set of 250,000 regression coefficients from the full model space and fit a multinomial logistic regression to predict whether an estimate is “negative significant,” “positive significant,” or “not significant” (significance class). We then calculated the average change in predicted probabilities by altering one model specification at a time to assess the impact of each specification choice, subsequently averaging across all independent variables.

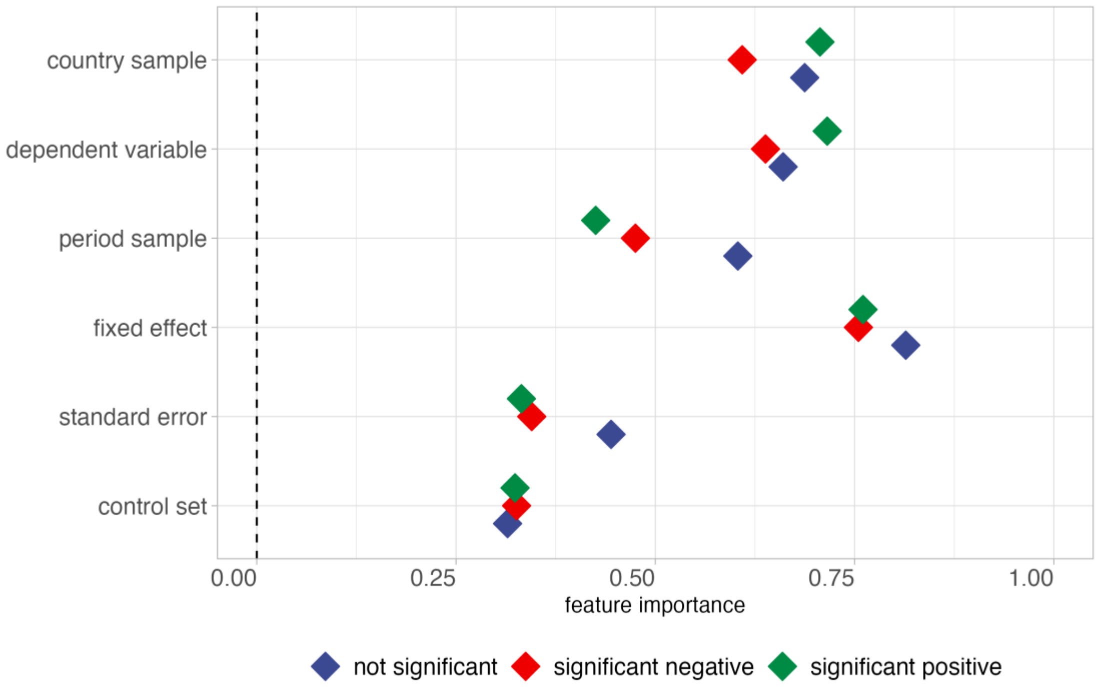

Figure S3.3.2. Average change in feature importance scores (SHAP values) across all independent variables for the prediction of the significance class. The figure shows the average change in predicted probabilities of six different model specification choices (x-axis) averaged across all independent variables under consideration. To estimate these averages changes in predicted probabilities, we extracted a random set of 250,000 regression coefficients from the full model space and then fit a deep learning model to build a classifier that predicts whether an estimate is “negative significant”, “positive significant” or “not significant”. Afterwards, we estimate the average SHAP values to assess the relevance of each model specification choice.

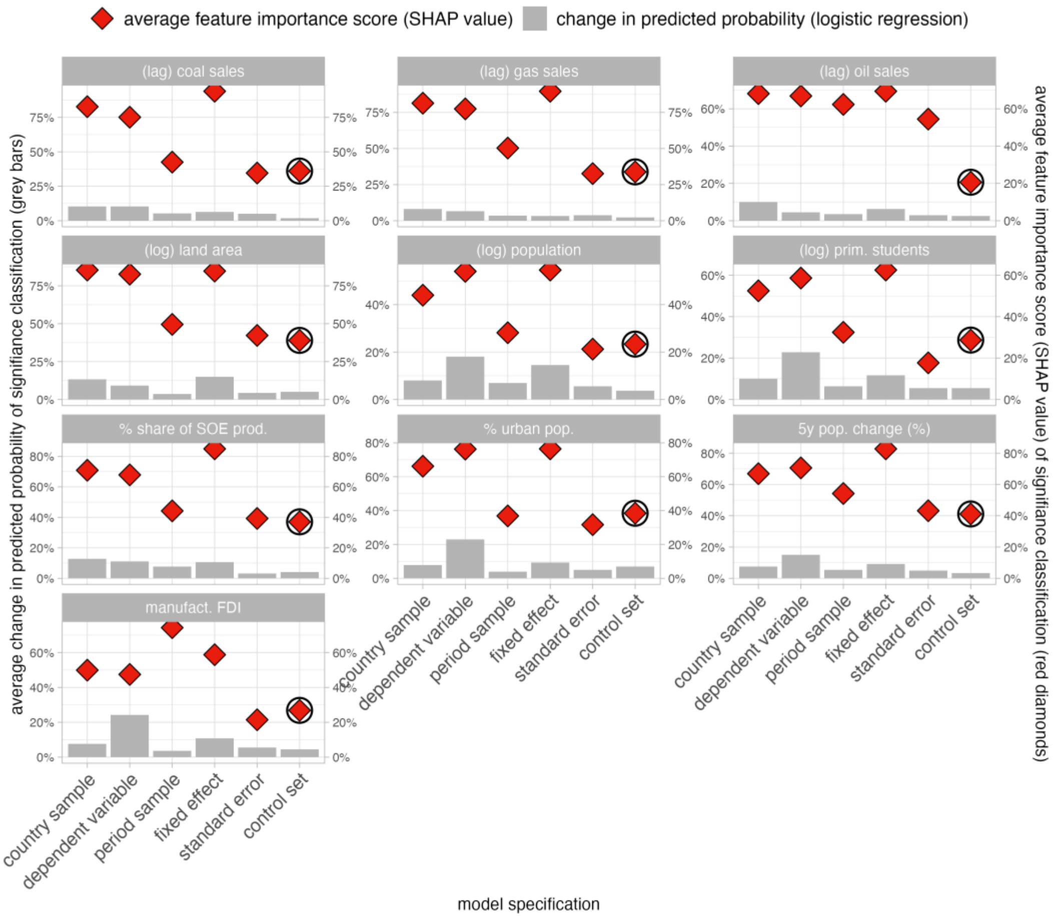

Figure S3.3.3: Average change in predicted probabilities and average feature importance scores (SHAP values) for each model specification binary in predicting the significance class. The figure presents the average change in predicted probabilities for six distinct model specification choices across the independent variables. To estimate these average changes in predicted probabilities (grey bars), we sampled a random set of 250,000 regression coefficients from the full model space and fit a multinomial logistic regression to predict whether an estimate is "negative significant," "positive significant," or "not significant" (significance class). We then calculated the average change in predicted probabilities by modifying one model specification at a time to assess the impact of each specification choice. For SHAP values (red diamonds), we used the same random sample of estimations as for the multinomial logistic regression. We applied a deep learning model to classify the significance class of each coefficient, then computed the average SHAP values to evaluate the importance of each model specification choice.

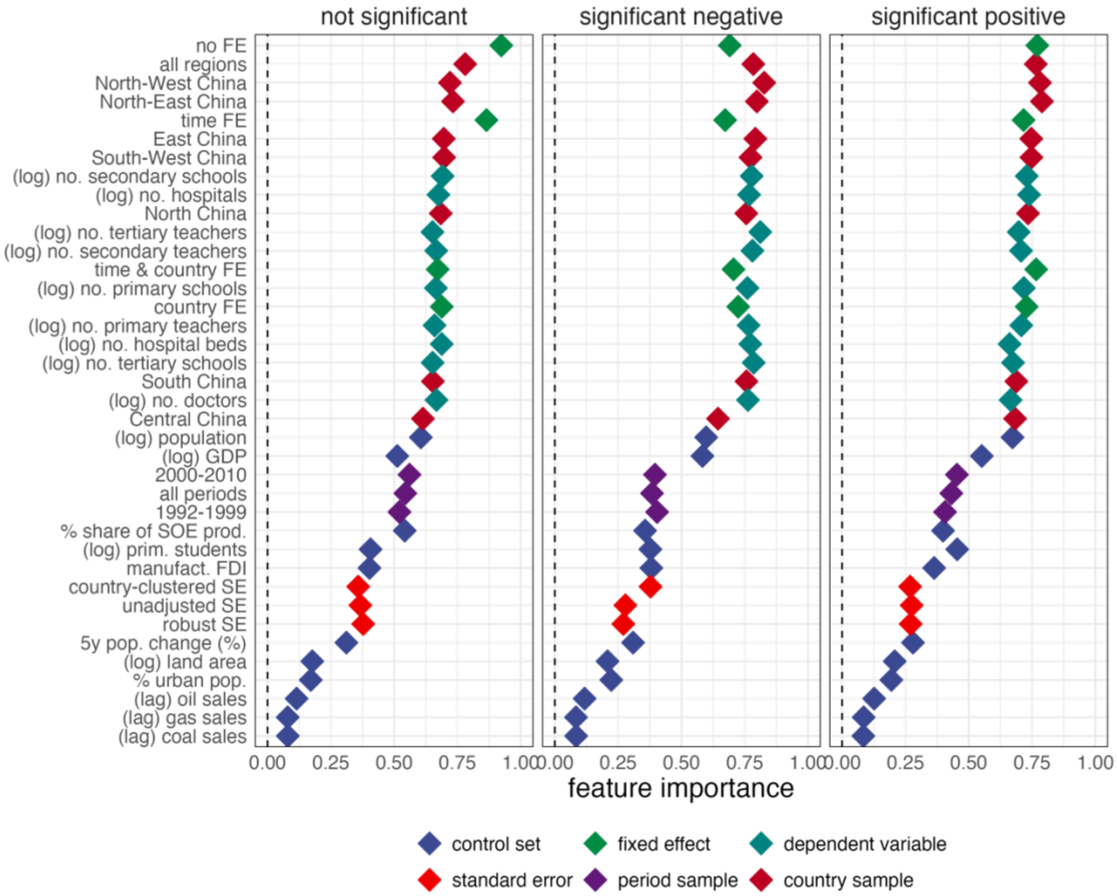

Figure S3.3.4. Feature importance scores (SHAP values) of specification binaries. The figure shows the average feature importance scores of all model specifications (x-axis) averaged across all independent variables under consideration. To estimate these scores, we extracted a random set of 250,000 regression coefficients from the full model space and then fit a deep learning model to build a classifier that predicts whether an estimate is "negative significant", "positive significant" or "not significant". Afterwards, we estimate the average SHAP values to assess the relevance of each model specification choice.

S4: Case 4: Individual Trust in Institutions

S4.1: Literature review and data selection

To identify the determinants of individuals' trust in public institutions, we draw on the comprehensive literature review by Nannestad (2008). The factors associated with trust can be grouped into broader theoretical debates in political science. Socioeconomic status, encompassing factors such as education, income satisfaction, and subjective well-being (including happiness and health), plays a critical role in determining trust in public institutions (Helliwell, 2002; Putnam, 2000). Higher levels of education often correlate with greater trust, as education enhances individuals' understanding of institutional functions and fosters civic engagement (Bouckaert, 2002). Subjective economic satisfaction also contributes significantly: those content with their income tend to trust institutions more, perceiving them as effective in maintaining economic stability (Bjornskov, 2008). Furthermore, subjective well-being, including happiness and health, aligns with higher trust levels, as healthier and happier individuals may view institutions as supportive and effective in ensuring their well-being (Huppert, 2005). In the next paragraphs, we discuss these determinants in more detail.

First, demographic characteristics, including age, gender, and household size, offer insights into variations in institutional trust (Delhey & Newton, 2003). Older individuals generally exhibit higher trust levels, possibly due to their longer-term interactions and experiences with institutions. Gender differences, while context-dependent, suggest that women might have varied trust levels based on their experiences with institutional bias or their levels of political participation. Household size can influence trust as well since larger households may face resource constraints and internal conflicts, which can reduce collective trust in external institutions (Luhmann, 1979).

Second, the urban-rural divide is another significant theoretical lens through which trust in institutions can be understood. Urban residents often display lower trust levels compared to their rural counterparts, potentially due to the higher levels of diversity, anonymity, and perceived inefficiencies in urban settings (Kasara & Suryanarayan, 2008). This dynamic highlights the role of community cohesion and perceived institutional responsiveness in shaping trust.

Third, political engagement and ideological orientation are crucial in understanding trust in public institutions (Dalton, 2004). Individuals with higher political interest tend to exhibit greater trust, as their engagement reflects a deeper understanding and more informed perspective on institutional roles. Past voting behavior can also signal different trust levels: those who participate in elections are more likely to view institutions as legitimate and responsive (Norris, 1999). Additionally, political ideology influences trust levels, with those aligned with mainstream or ruling parties generally exhibiting higher trust, while those on political fringes may distrust institutions, viewing them as biased or ineffective (Rose, 2001).

Fourth, social capital, reflected in factors such as union membership and religiosity, significantly impacts institutional trust (Putnam, 1995). Union members often trust institutions more due to their involvement in organized groups that engage with governmental bodies, fostering a sense of influence and efficacy. Religiosity also correlates with higher trust levels, as religious teachings and community activities promote social cohesion and reinforce positive perceptions of societal structures (Paxton, 2002). Furthermore, satisfaction with government performance is a direct predictor of institutional trust; positive evaluations of government effectiveness and fairness enhance trust (Levi & Stoker, 2000).

Fifth, psychological and emotional factors, including subjective happiness and health, play a vital role in shaping trust in public institutions. Happier and healthier individuals may have a more positive outlook on life and institutions, believing that the system supports their well-being

effectively. This connection underscores the importance of perceived personal well-being in fostering institutional trust (Dolan et al., 2008).

To explore these potential determinants of trust in public institutions, we use data from the European Social Surveys (ESS) wave 1-10 (European Social Survey, 2020), which covers bi-yearly surveys from 2002 to 2020. This dataset provides extensive coverage of European countries, enabling a robust analysis of trust in public institutions across different contexts and time periods. Based on the literature review by Nannestad (2008), we select 15 key independent variables to examine model uncertainty. A detailed description of all independent variables including summary statistics is provided in Table S4.1.1. These variables include demographic factors (age, gender, household size), socioeconomic indicators (years of education, income satisfaction), political engagement (left-right scale, union membership, political interest, voting behavior), and personal well-being metrics (urban living, religiosity, satisfaction with government, subjective happiness, subjective health).

For our analysis, we define several country and period samples to account for regional and temporal variations. The country samples are categorized as follows: core EU countries, including Germany, France, Great Britain, Italy, and Spain; small EU countries, including Austria, Belgium, Cyprus, Ireland, Portugal, Luxembourg, and the Netherlands; Scandinavian countries, including Sweden, Finland, Denmark, Norway, and Iceland; and Eastern European countries, including Bulgaria, Czech Republic, Estonia, Croatia, Lithuania, Latvia, Poland, Romania, Slovenia, and Slovakia. Additionally, we include an all-encompassing sample covering all these countries.

The period samples are divided into five temporal groups based on the ESS waves: wave 1-2, wave 3-4, wave 5-6, wave 7-8, and wave 9-10. To address debates about the operationalization of the outcome variable, we use different trust indicators. These include trust in politicians, trust in parliament, trust in the European Parliament, and trust in the legal system (Newton, 2001). These indicators are based on the survey question “How much trust do you have in institution X?” which can be answered on a four-point scale ranging from 1 (no trust at all) to 4 (very high trust).

Similar to the previous cases, we apply different fixed effect structures, specifically using year fixed effects only, country fixed effects only, both country and wave fixed effects, and no fixed effects. Finally, we use three types of standard errors: simple/unadjusted standard error, heteroscedasticity-robust standard error, and the Huber-White standard error clustered at the country level.

With 15 independent variables, we create a control set for each combination of variables, resulting in a total of 32,767 control sets. Each of these sets is used in combination with five country samples, five period samples, four dependent variables, four fixed effect structures, and three standard error types. Thus, in total, with 32,767 control sets and 1,200 model specifications (5 country samples × 5 period samples × 4 dependent variables × 4 fixed effect structures × 3 standard error types), we end up with 39,320,400 regressions in the entire model space. These regressions include approximately 294.9 million estimates (coefficient and standard error combinations).

Table S4.1.1. Variable and summary statistics

Variable	N	Mean	St. Dev.	Min	Max
trust in EU parliament	438,259	4.432	2.537	0	10
trust in legal system	477,862	5.102	2.734	0	10
trust in politicians	480,507	3.487	2.433	0	10
trust in parliament	477,382	4.380	2.634	0	10
average trust (pca)	429,089	0.000	1.668	−3.368	4.399
average trust (mean)	429,089	4.374	2.143	0.000	10.000
female	489,588	0.537	0.499	0	1
age	487,263	48.536	18.629	13	123
years of education	483,047	12.456	4.109	0.000	76.000
union member	483,780	0.174	0.379	0	1
urban living	490,555	0.332	0.471	0	1
religiosity	485,578	4.679	3.054	0	10
household size	489,163	2.724	1.444	1	24
left-right scale	421,392	5.115	2.265	0	10
political interest	488,914	2.626	0.913	1	4
feeling about income	479,959	2.058	0.883	1	4
satisfaction with gov.	472,628	4.216	2.515	0	10
voted in last election	485,469	0.721	0.448	0	1
subjective health	489,656	2.230	0.933	1	5

S4.2: Significance shares by model specifications

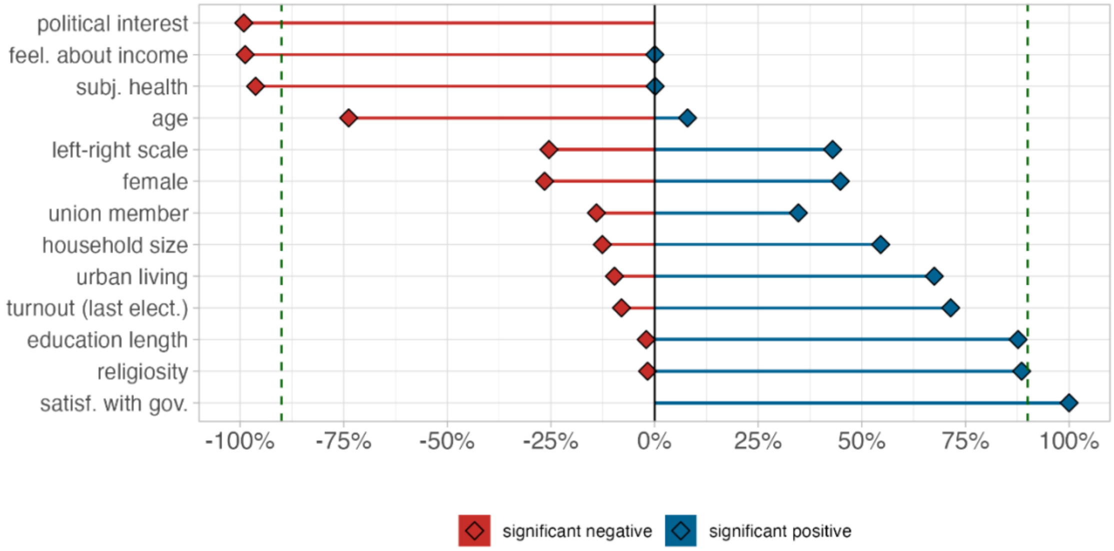

Figure S4.2.1: Significance shares of the independent variables. The figure plots the share of (positive and negative) significant coefficients (blue and red, respectively) of all independent variables in the full model space. A coefficient is classified as ‘significant’ if its p-value is below 0.1. The dashed line indicates 90%.

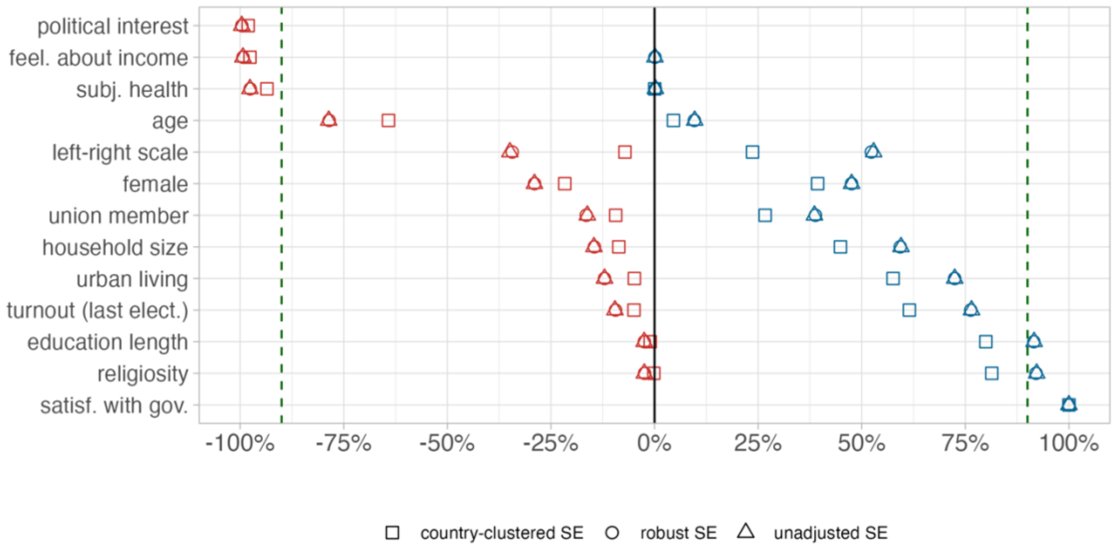

Figure S4.2.2: Significance shares of the independent variables by different standard error types. The figure plots the share of (positive and negative) significant coefficients (blue and red, respectively) of all independent variables by different standard error types in the full model space. A coefficient is classified as 'significant' if its p-value is below 0.1. The dashed line indicates 90%.

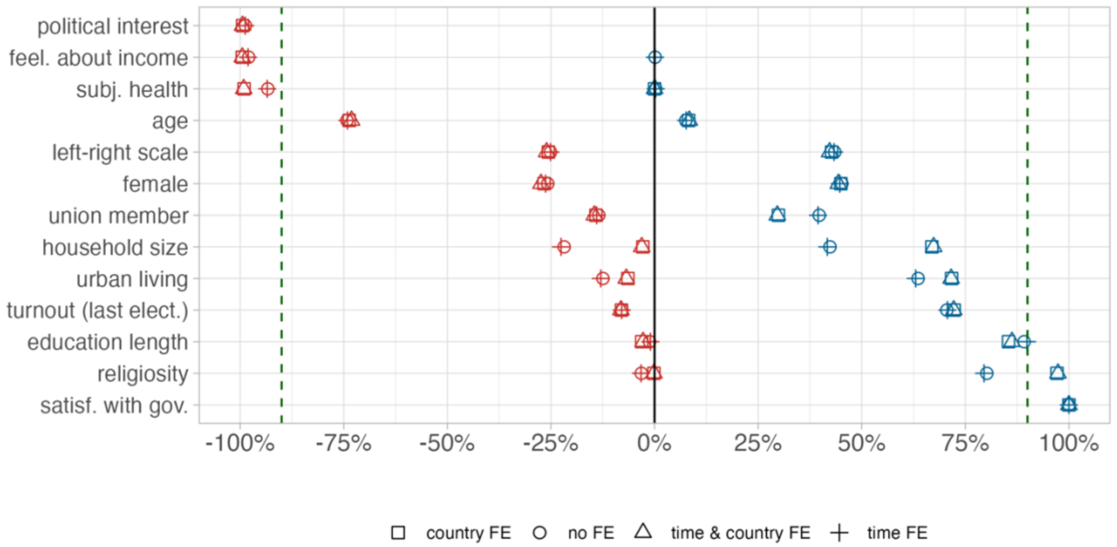

Figure S4.2.3: Significance shares of the independent variables by different fixed effect structures. The figure plots the share of (positive and negative) significant coefficients (blue and red, respectively) of all independent variables by different fixed effect structures in the full model space. A coefficient is classified as 'significant' if its p-value is below 0.1. The dashed line indicates 90%.

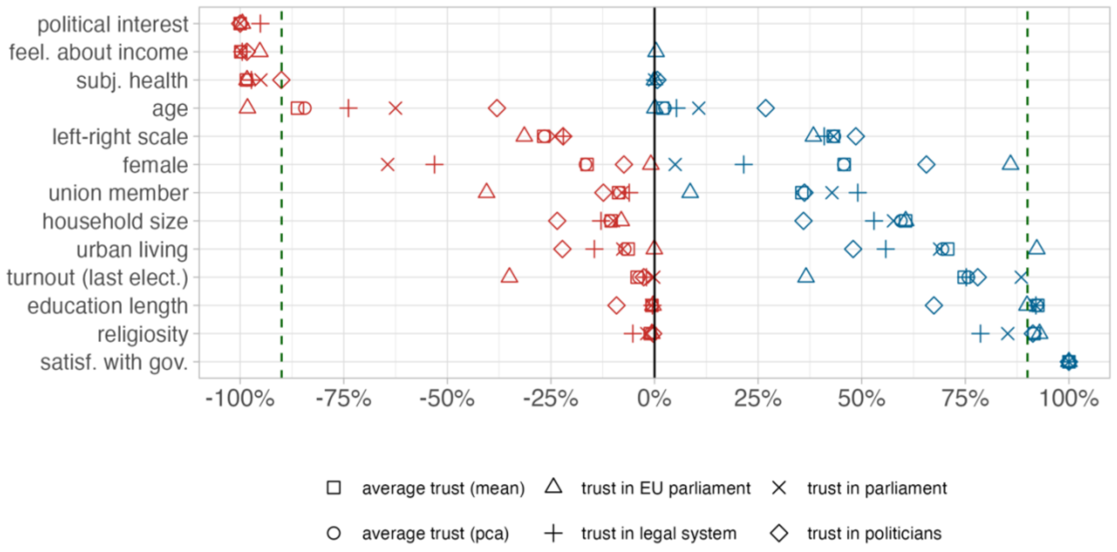

Figure S4.2.4: Significance shares of the independent variables by different dependent variables. The figure plots the share of (positive and negative) significant coefficients (blue and red, respectively) of all independent variables by different dependent variables in the full model space. A coefficient is classified as 'significant' if its p-value is below 0.1. The dashed line indicates 90%.

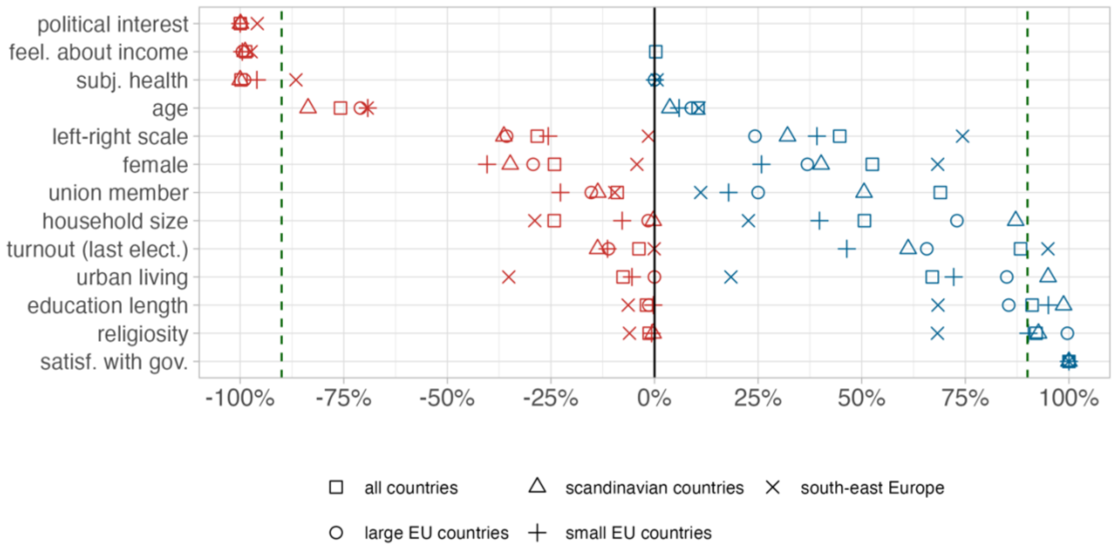

Figure S4.2.5: Significance shares of the independent variables by different country samples. The figure plots the share of (positive and negative) significant coefficients (blue and red, respectively) of all independent variables by different country samples in the full model space. A coefficient is classified as ‘significant’ if its p-value is below 0.1. The dashed line indicates 90%.

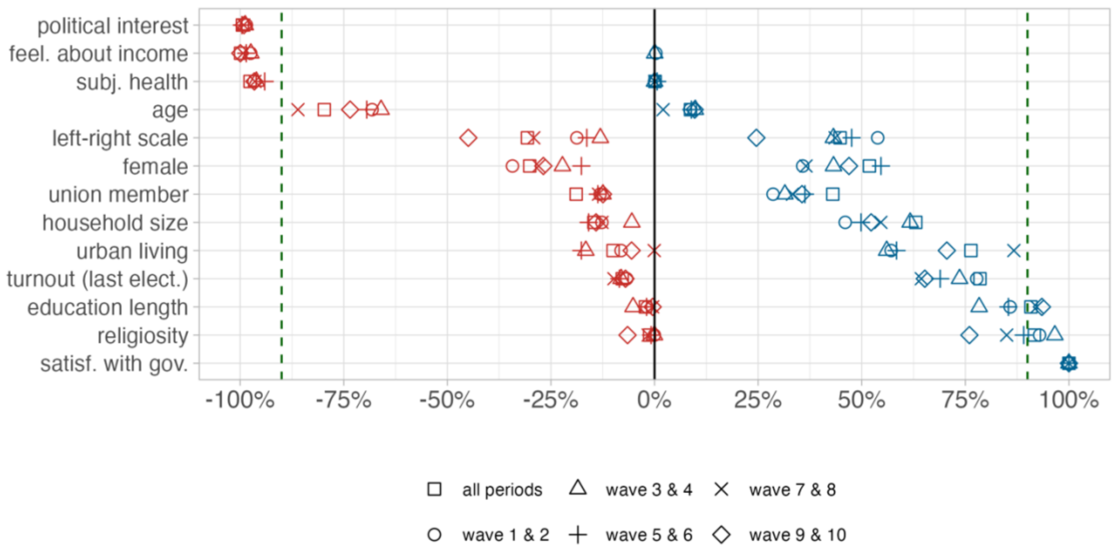

Figure S4.2.6: Significance shares of the independent variables by different period samples. The figure plots the share of (positive and negative) significant coefficients (blue and red, respectively) of all independent variables by different period samples in the full model space. A coefficient is classified as 'significant' if its p-value is below 0.1. The dashed line indicates 90%.

S4.3: Impact of model specification on significance of estimates

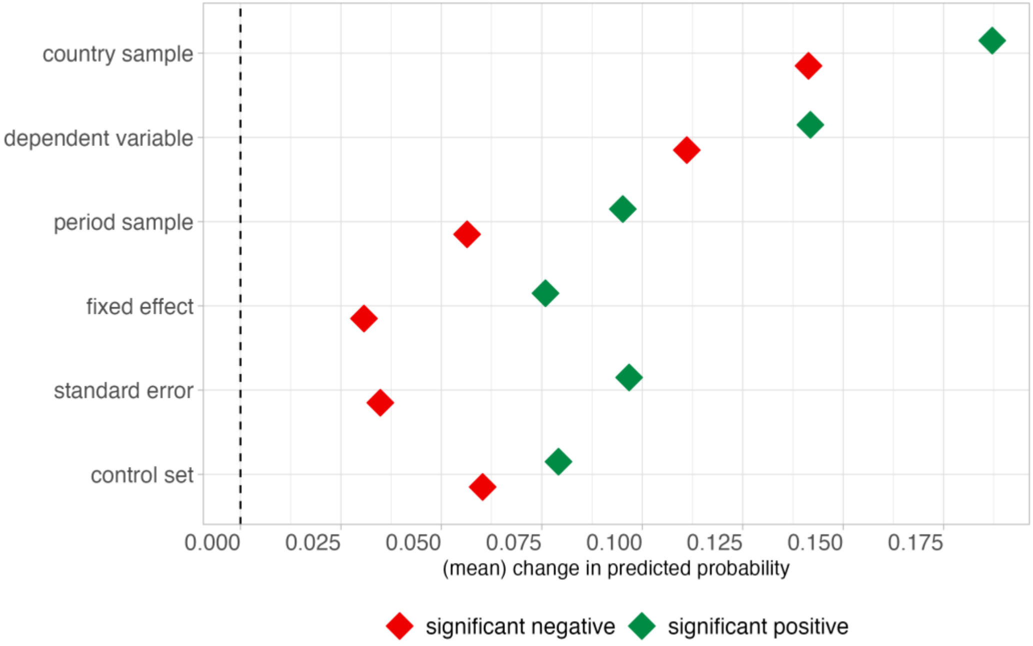

Figure S4.3.1: Average change in predicted probability for predicting the significance class after modifying one model specification. The figure displays the average change in predicted probabilities for six different model specification choices (y-axis), averaged across all independent variables. To estimate these average changes in predicted probabilities, we sampled a random set of 250,000 regression coefficients from the full model space and fit a multinomial logistic regression to predict whether an estimate is "negative significant," "positive significant," or "not significant" (significance class). We then calculated the average change in predicted probabilities by altering one model specification at a time to assess the impact of each specification choice, subsequently averaging across all independent variables.

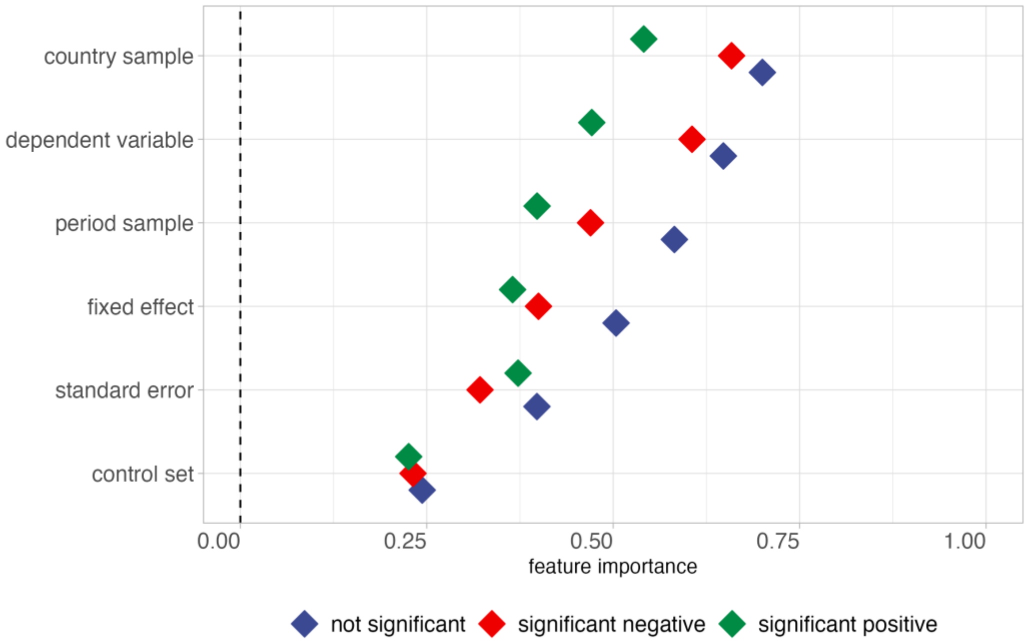

Figure S4.3.2: Average change in feature importance scores (SHAP values) across all independent variables for the prediction of the significance class. The figure shows the average change in predicted probabilities of six different model specification choices (x-axis) averaged across all independent variables under consideration. To estimate these averages changes in predicted probabilities, we extracted a random set of 250,000 regression coefficients from the full model space and then fit a deep learning model to build a classifier that predicts whether an estimate is “negative significant”, “positive significant” or “not significant”. Afterwards, we estimate the average SHAP values to assess the relevance of each model specification choice.

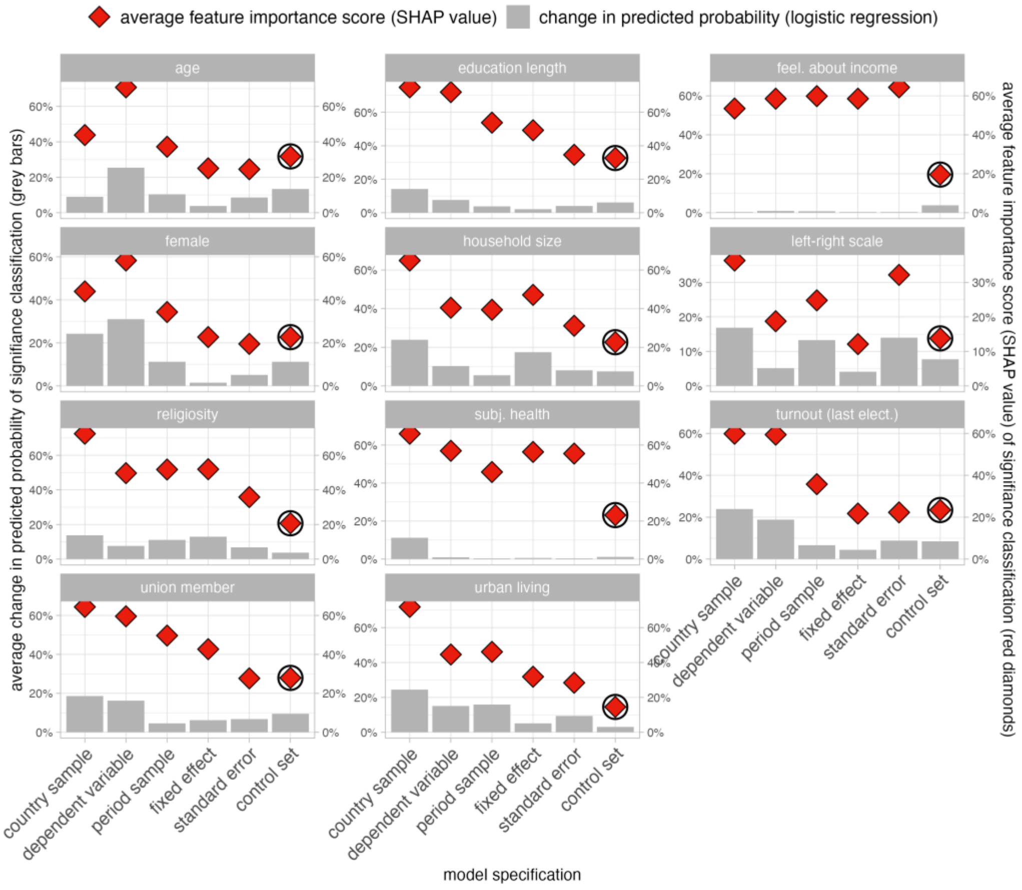

Figure S4.3.3: Average change in predicted probabilities and average feature importance scores (SHAP values) for each model specification binary in predicting the significance class. The figure presents the average change in predicted probabilities for six distinct model specification choices across the independent variables. To estimate these average changes in predicted probabilities (grey bars), we sampled a random set of 250,000 regression coefficients from the full model space and fit a multinomial logistic regression to predict whether an estimate is "negative significant," "positive significant," or "not significant" (significance class). We then calculated the average change in predicted probabilities by modifying one model specification at a time to assess the impact of each specification choice. For SHAP values (red diamonds), we used the same random sample of estimations as for the multinomial logistic regression. We applied a deep learning model to classify the significance class of each coefficient, then computed the average SHAP values to evaluate the importance of each model specification choice.

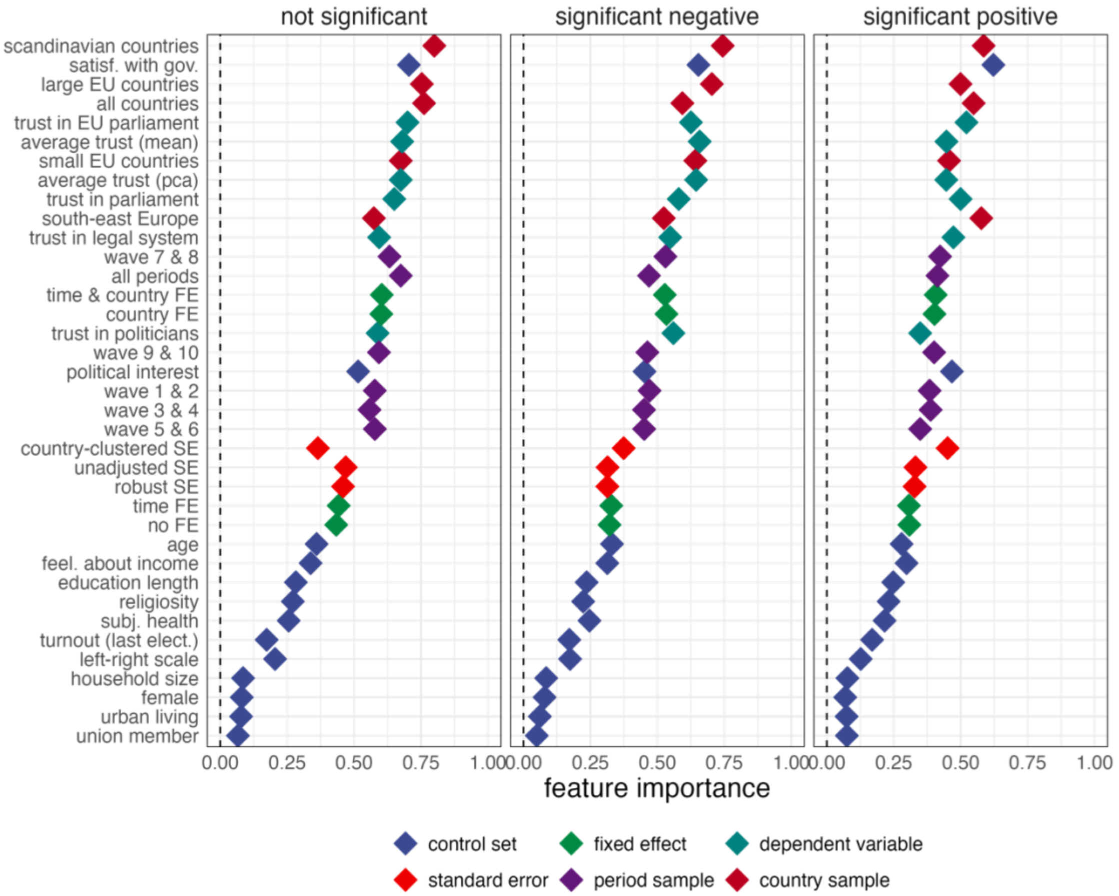

Figure S4.3.4. Feature importance scores (SHAP values) of specification binaries. The figure shows the average feature importance scores of all model specifications (x-axis) averaged across all independent variables under consideration. To estimate these scores, we extracted a random set of 250,000 regression coefficients from the full model space and then fit a deep learning model to build a classifier that predicts whether an estimate is "negative significant", "positive significant" or "not significant". Afterwards, we estimate the average SHAP values to assess the relevance of each model specification choice.

S5: Details about the neural networks and the grid search approach

To enhance the predictive performance and better account for non-linearities and interactions between specification choices, we adopt a machine learning approach. Using the same random set of estimations as in the multinomial logistic regression, we implement a deep learning model to classify estimates as “negative significant,” “positive significant,” or “not significant.” By employing a grid search and carefully tuning hyperparameters, we identify the optimal model structure and parameters for each independent variable. This ensures that the model generalizes well to unseen data, achieving strong precision, recall, accuracy, and F1-scores consistently above 90% (Figure S5.1.2).

The data is split into training, cross-validation (CV), and test sets in a 60-20-20 ratio. The model is trained for 10 epochs on the training set, with optimization based on performance on the validation set. The output layer comprises two units with a softmax activation function for class probability output. The model is compiled with the RMSprop optimizer, binary cross-entropy as the loss function, and accuracy and AUC as performance metrics. After training for 10 epochs, the best model configuration is chosen based on the highest validation accuracy. This grid search, paired with hyperparameter tuning, ensures a robust model that generalizes well to new data.

The neural network is a feedforward sequential model for binary classification, implemented using Keras. It includes an input layer matching the input data's dimensionality, followed by multiple hidden layers with ReLU activation. The number of hidden layers (5 or 7) and units per layer (32 or 64) are optimized through grid search, alongside the dropout rate (0 or 0.3) and learning rate (0.001). Dropout is applied after each hidden layer to prevent overfitting by randomly ignoring neurons during training. This comprehensive grid search optimizes the network's hyperparameters, allowing for the estimation of SHAP values that highlight the influence of each specification binary on the predicted significance class.

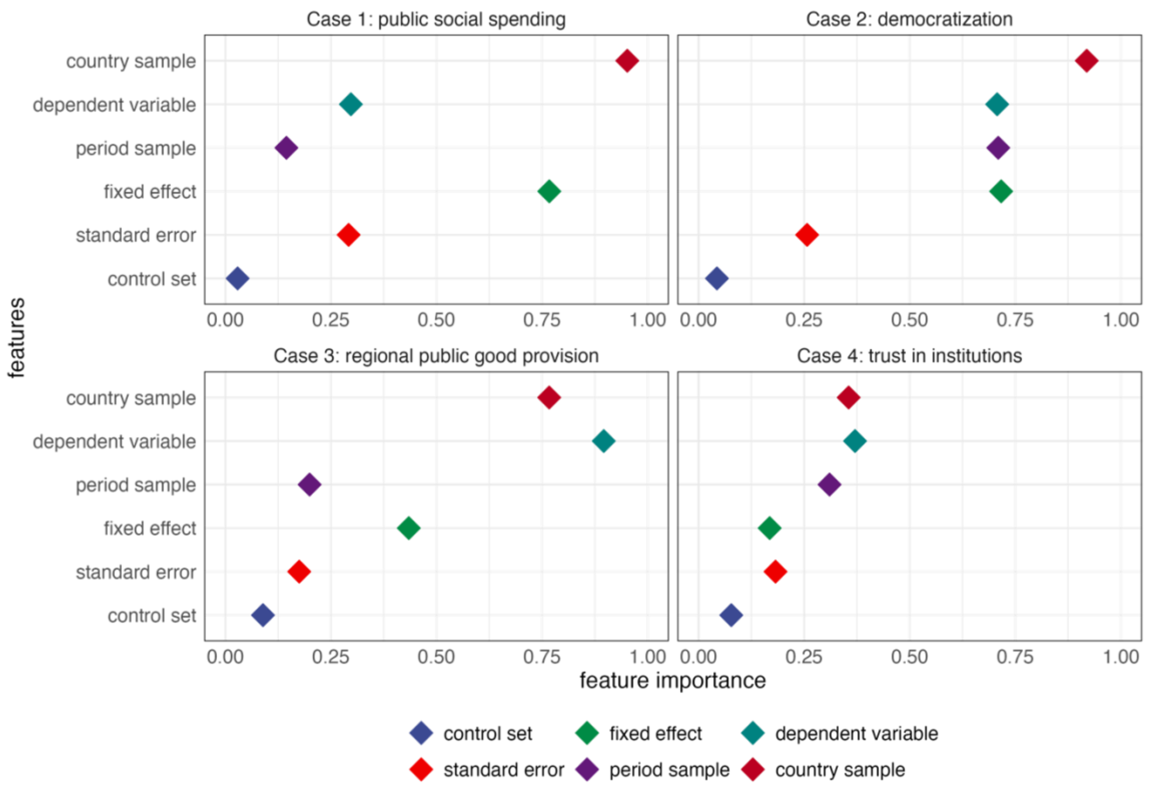

Figure S5.1.1. Feature Effect scores of model specification decisions. The panels show the feature effect scores for different model specification choices. To estimate them, we extracted a random set of 250,000 regression coefficients from the unrestricted model universe for each test case, and then fit a multi-layer neural network to predict whether an estimate is "negative significant", "positive significant" or "not significant". After employing a grid-search algorithm to define the most suitable hyperparameter structure, we use the best model with the highest classification accuracy to estimate the feature effect values (Accumulated Local Effects (ALE)) of each model specification binary.

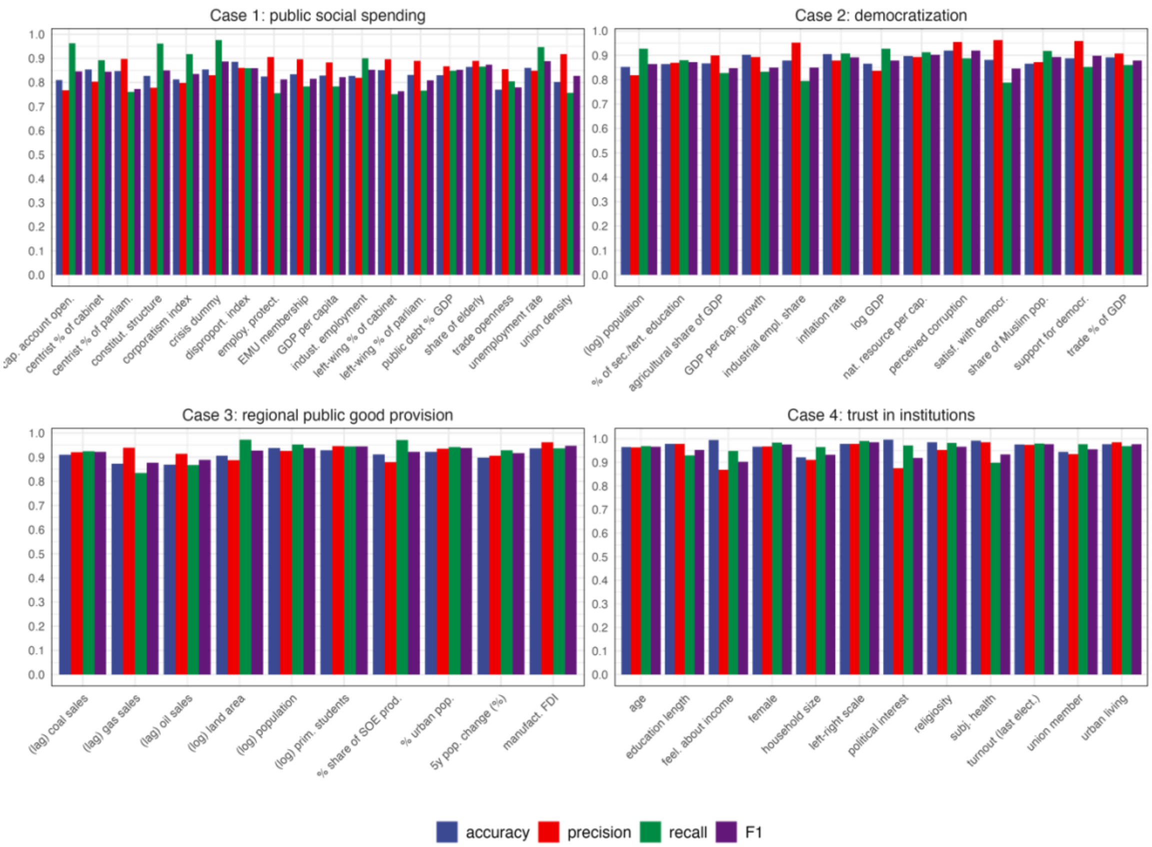

Figure S5.1.2. Classification performance of deep learning approach across different independent variables and performance metrices. The figure shows different performance metrices of the classifier based on the deep learning approach on the test set. The deep learning model is based on a grid-search approach for which we extracted a random set of 250,000 regression coefficients from the full model space and then fit a deep learning model to build a classifier that predicts whether an estimate is "negative significant", "positive significant" or "not significant". Afterwards, we apply the estimated model to the test set to determine accuracy, precision, recall and F1 for each independent variable within each case.

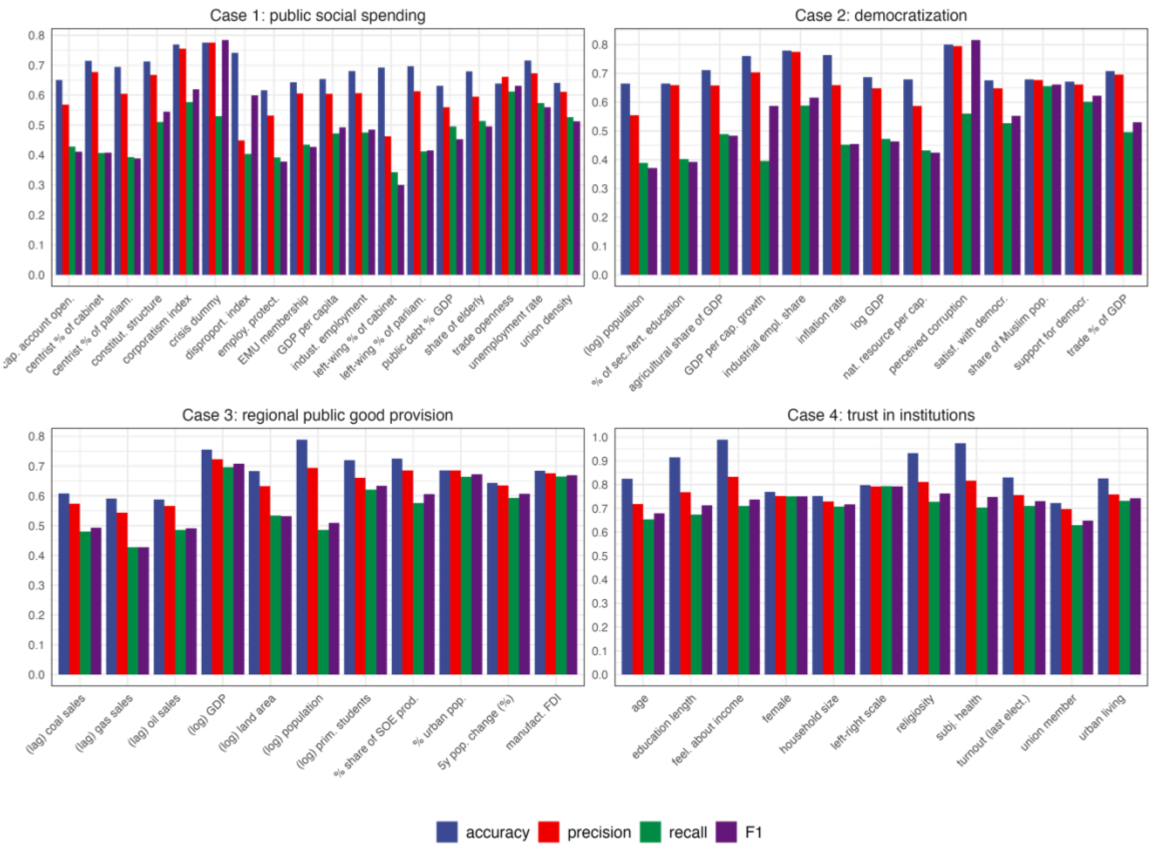

Figure S5.1.3. Classification performance of multinomial logistic regression across different independent variables and performance metrices. The figure shows different performance metrices of the classifier based on the multinomial logistic regression on the test set. To estimate the multinomial logistic regression, we extracted a random set of 250,000 regression coefficients from the full model space and then fit the model to build a classifier that predicts whether an estimate is “negative significant”, “positive significant” or “not significant”. Afterwards, we apply the estimated model to the test set to determine accuracy, precision, recall and F1 for each independent variable within each case**.**

S6: An R package for empirical social science

To facilitate the applied research of social science scholars conducting empirical analysis, we develop an R library that provides an off-the-shelf solution to identify the sources of model uncertainty and estimate sensitivity towards these model specification choices. The package is based on the code we have used for the analysis of this paper. The library will be available on CRAN and on our Github page. In general, beyond several visualization and helper functions, two functions will be at the core of the library.

The first function creates an object that contains the model universe along with the relevant meta data. Here, we focus on the key model specification assumptions that have been discussed in this paper, namely the dependent variable choice, fixed effect structure, standard error type, and sample selection. In addition, since the size of the model space increases exponentially with the number of model specifications and control variables, we allow the user to parallelize the estimation of the model universe.

The second function uses the result object of the estimation function as an input to conduct multinomial logistic regression analysis and the (grid-search-based) deep learning approach to predict the significance classification based on the model specification. To execute this function, users are required to install Keras and Tensorflow beforehand. The function will enable users to set pre-defined hyperparameters, e.g. type of loss function, number of layers, number of units, type of activation function, amongst others. In addition to the grid search, the function will also implement the estimation of the odds ratios, changes in predicted probabilities and the feature importance scores for each model specification. To provide most flexibility to the user, the function will enable users to select among different feature importance metrices, e.g. SHAP, LIME, amongst others.

S7: SI References

1. Leamer, E. E. (1983). Let's take the con out of econometrics. *The American Economic Review*, *73*(1), 31–43. [cite: 113]

2. Leamer, E. E. (1985). Sensitivity analyses would help. *The American Economic Review*, *75*(3), 308–313. [cite: 114]

3. King, G., & Roberts, M. E. (2015). How robust standard errors expose methodological problems they do not fix, and what to do about it. *Political Analysis*, *23*(2), 159–179. [cite: 99]

4. Abadie, A., Athey, S., Imbens, G. W., & Wooldridge, J. (2017). When should you adjust standard errors for clustering? (w24003). [cite: 1]

5. Neumayer, E., & Plümper, T. (2017). *Robustness tests for quantitative research*. Cambridge University Press. [cite: 132]

6. Allan, J. P., & Scruggs, L. (2004). Political partisanship and welfare state reform in advanced industrial societies. *American Journal of Political Science*, *48*(3), 496–512. [cite: 6, 7, 8]

7. Clasen, J., & Siegel, N. A. (Eds.). (2007). *Investigating welfare state change: The 'dependent variable problem' in comparative analysis*. Edward Elgar Publishing. [cite: 37, 38]

8. Armingeon, K., Isler, C., Knöpfel, L., Weisstanner, D., & Engler, S. (2013). *Codebook: Comparative political data set 1960-2013*. Institute of Political Science, University of Bern. [cite: 10, 11]

9. IMF. (2021). *World economic outlook database: October 2021*. https://www.imf.org/en/Publications/WEO/weo-database/2021/October [cite: 87]

10. Jahn, D. (2016). Changing of the guard: Trends in corporatist arrangements in 42 highly industrialized societies from 1960 to 2010. *Socio-Economic Review*, *14*(1), 47–71. [cite: 96]

11. Scruggs, L. (2014). *Social welfare generosity scores in CWED 2: A methodological genealogy* (1). Comparative Welfare Entitlements Dataset Working Paper. [cite: 158]

12. Claassen, C. (2020). Does public support help democracy survive? *American Journal of Political Science*, *64*(1), 118–134. [cite: 36, 37]

13. Acemoglu, D., & Robinson, J. A. (2009). *Economic origins of dictatorship and democracy*. Cambridge University Press. [cite: 4]

14. Lipset, S. M. (1959). Some social requisites of democracy: Economic development and political legitimacy. *American Political Science Review*, *53*(1), 69–105. [cite: 120]

15. Milner, H. V. (1998). International trade and environmental policy in the 1990s. *International Organization*, *52*(3), 759–760. [cite: 129, 130]

16. Inglehart, R. (1997). *Modernization and postmodernization: Cultural, economic, and political change in 43 societies*. Princeton University Press. [cite: 88]

17. Barro, R. J. (1999). Determinants of democracy. *Journal of Political Economy*, *107*(S6), S158–S183. [cite: 15]

18. Huntington, S. P. (1993). The clash of civilizations? *Foreign Affairs*, *72*(3), 22–49. [cite: 85]

19. Treisman, D. (2007). What have we learned about the causes of corruption from ten years of cross-national empirical research? *Annual Review of Political Science*, *10*, 211–244. [cite: 178, 179]

20. Sung, H.-E. (2004). Democracy and political corruption: A cross-national comparison. *Crime, Law and Social Change*, *41*(2), 179–193. [cite: 170]

21. Diamond, L. (1999). *Developing democracy: Toward consolidation*. Johns Hopkins University Press. [cite: 45]

22. Ross, M. L. (2001). Does oil hinder democracy? *World Politics*, *53*(3), 325–361. [cite: 150]

23. Huber, E., Ragin, C., & Stephens, J. D. (1993). Social democracy, Christian democracy, constitutional structure, and the welfare state. *American Journal of Sociology*, *99*(3), 711–749. [cite: 81]

24. Hong, J. Y. (2018). How natural resources affect authoritarian leaders' provision of public services: Evidence from China. *The Journal of Politics*, *80*(1), 178–194. [cite: 79, 80]

25. Acemoglu, D., Johnson, S., & Robinson, J. A. (2001). The colonial origins of comparative development: An empirical investigation. *American Economic Review*, *91*(5), 1369–1401. [cite: 2, 3]

26. Barro, R. J. (1991). Economic growth in a cross section of countries. *Quarterly Journal of Economics*, *106*(2), 407–443. [cite: 14]

27. Sachs, J. D., & Warner, A. M. (1995). Natural resource abundance and economic growth. *NBER Working Paper*, *5398*. [cite: 154, 155]

28. Borensztein, E., De Gregorio, J., & Lee, J.-W. (1998). How does foreign direct investment affect economic growth? *Journal of International Economics*, *45*(1), 115–135. [cite: 21, 22]

29. Bloom, D. E., Canning, D., & Sevilla, J. (2004). The impact of health on economic growth: A production function approach. *World Development*, *32*(1), 1–13. [cite: 17, 18]

30. Easterly, W., & Levine, R. (2003). Tropics, germs, and crops: How endowments influence economic development. *Journal of Monetary Economics*, *50*(1), 3–39. [cite: 50, 51]

31. Glaeser, E. L., La Porta, R., Lopez-de-Silanes, F., & Shleifer, A. (2004). Do institutions cause growth? *Journal of Economic Growth*, *9*(3), 271–303. [cite: 68, 69]

32. Cutright, P. (1965). Political structure, economic development, and national social security programs. *American Journal of Sociology*, *70*(5), 537–550. [cite: 41]

33. Jackman, R. W. (1974). Political democracy and social equality: A comparative analysis. *American Sociological Review*, *39*(1), 29–45. [cite: 94, 95]

34. Wilensky, H. L., & Lebeaux, C. N. (1958). *Industrial society and social welfare: The impact of industrialization on the supply and organization of social welfare services in the united states*. [cite: 188]

35. Wilensky, H. L. (1974). *The welfare state and equality: Structural and ideological roots of public expenditures* (Vol. 140). University of California Press. [cite: 187]

36. Offe, C. (1972). Advanced capitalism and the welfare state. *Politics & Society*, *2*(4), 479–488. [cite: 135]

37. Gough, I. (1979). *The political economy of the welfare state*. Macmillan International Higher Education. [cite: 70]

38. Bowles, S., & Gintis, H. (1982). The crisis of liberal democratic capitalism: The case of the united states. *Politics & Society*, *11*(1), 51–93. [cite: 25, 26]

39. (No "Piven & Cloward, 1982" in provided references)

40. Miliband, R. (1969). *The state in capitalist society*. Weidenfeld; Nicolson. [cite: 128, 129]

41. Domhoff, G. W. (1972). *Fat cats and democrats: The role of the big rich in the party of the common man*. Prentice-Hall. [cite: 49]

42. (No "Block, 1987" in provided references)

43. (No "Przeworski, 1986" in provided references)

44. Briggs, A. (1961). The welfare state in historical perspective. *European Journal of Sociology/Archives Européennes de Sociologie/Europäisches Archiv für Soziologie*, *2*(2), 221–258. [cite: 27]

45. Marshall, T. H., & Bottomore, T. B. (1992). *Citizenship and social class* (Vol. 2). Pluto Press. [cite: 126]

46. Flora, P., & Heidenheimer, A. J. (Eds.). (1981). *The development of welfare states in Europe and America*. Transaction Publishers. [cite: 60, 61]

47. Haggard, S., & Kaufman, R. R. (2020). *Development, democracy, and welfare states*. Princeton University Press. [cite: 71]

48. Cameron, K. (1978). Measuring organizational effectiveness in institutions of higher education. *Administrative Science Quarterly*, 604–632. [cite: 34]

49. Shalev, M. (1983). The social democratic model and beyond: Two generations of comparative research on the welfare state. *Comparative Social Research*, *6*(3), 315–351. [cite: 159, 160]

50. Castles, F. G., & Mitchell, D. (1993). World of welfare and families of nations. In *Families of nations: Patterns of public policy in western democracies*. Aldershot, Dartmouth. [cite: 35, 36]

51. Korpi, W. (1978). *The working class in welfare capitalism: Work, unions and politics in Sweden*. Routledge & Kegan Paul. [cite: 103]

52. Korpi, W. (1980). Social policy and distributional conflict in the capitalist democracies. A preliminary comparative framework. *West European Politics*, *3*(3), 296–316. [cite: 104, 105]

53. (No "Hicks, 1999" in provided references)

54. Stephens, J. D. (1979). *The transition from capitalism to socialism*. Springer. [cite: 168]

55. Esping-Andersen, G. (1990). *The three worlds of welfare capitalism*. Princeton University Press. [cite: 53]

56. Ferragina, E., & Seeleib-Kaiser, M. (2011). Thematic review: Welfare regime debate: Past, present, futures? *Policy & Politics*, *39*(4), 583–611. [cite: 58, 59]

57. Van Kersbergen, K., & Vis, B. (2015). Three worlds' typology: Moving beyond normal science? *Journal of European Social Policy*, *25*(1), 111–123. [cite: 184]

58. Arts, W. A., & Gelissen, J. (2010). Models of the welfare state. In *The oxford handbook of the welfare state*. [cite: 11, 12]

59. Skocpol, T. (1980). Political response to capitalist crisis: Neo-marxist theories of the state and the case of the new deal. *Politics & Society*, *10*(2), 155–201. [cite: 161, 162]

60. Skocpol, T., & Amenta, E. (1986). States and social policies. *Annual Review of Sociology*, *12*(1), 131–157. [cite: 164]

61. Skocpol, T. (1992). State formation and social policy in the united states. *American Behavioral Scientist*, *35*(4-5), 559–584. [cite: 163]

62. Immergut, E. M. (1992). *Health politics: Interests and institutions in western Europe*. CUP Archive. [cite: 88]

63. Steinmo, S. (1993). *Taxation and democracy: Swedish, British, and American approaches to financing the modern state*. Yale University Press. [cite: 165]

64. Thelen, K. (1999). Historical institutionalism in comparative politics. *Annual Review of Political Science*, *2*(1), 369–404. [cite: 176]

65. Tsebelis, G. (1995). Decision making in political systems: Veto players in presidentialism, parliamentarism, multicameralism and multipartyism. *British Journal of Political Science*, *25*(3), 289–325. [cite: 180, 181]

66. Van Kersbergen, K. (2003). *Social capitalism: A study of Christian democracy and the welfare state*. Routledge. [cite: 182]

67. Van Kersbergen, K., & Manow, P. (Eds.). (2009). *Religion, class coalitions, and welfare states*. Cambridge University Press. [cite: 183]

68. Jensen, C. (2014). *The right and the welfare state*. OUP Oxford. [cite: 97]

69. Pierson, P. (1994). *Dismantling the welfare state?: Reagan, Thatcher and the politics of retrenchment*. Cambridge University Press. [cite: 139]

70. Pierson, P. (2001). Post-industrial pressures on the mature welfare state. In *The new politics of the welfare state* (pp. 80–104). [cite: 140]

71. Pontusson, J. (1995). From comparative public policy to political economy: Putting political institutions in their place and taking interests seriously. *Comparative Political Studies*, *28*(1), 117–147. [cite: 142, 143]

72. Boix, C. (2000). Partisan governments, the international economy, and macroeconomic policies in advanced nations, 1960-93. *World Politics*, *52*(1), 38–73. [cite: 19]

73. Rueda, D. (2007). *Social democracy inside out: Partisanship and labor market policy in advanced industrialized democracies*. Oxford University Press on Demand. [cite: 153]

74. Vlandas, T. (2013). Mixing apples with oranges? Partisanship and active labour market policies in Europe. *Journal of European Social Policy*, *23*(1), 3–20. [cite: 185, 186]

75. Hall, P. A., & Soskice, D. (Eds.). (2001). *Varieties of capitalism: The institutional foundations of comparative advantage*. OUP Oxford. [cite: 73]

76. Hancké, B. (Ed.). (2009). *Debating varieties of capitalism: A reader*. Oxford University Press on Demand. [cite: 74]

77. Estevez-Abe, M. (2005). Gender bias in skills and social policies: The varieties of capitalism perspective on sex segregation. *Social Politics: International Studies in Gender, State & Society*, *12*(2), 180–215. [cite: 54, 55]

78. Mares, I. (2000). Strategic alliances and social policy reform: Unemployment insurance in comparative perspective. *Politics & Society*, *28*(2), 223–244. [cite: 125]

79. (No "Swenson, 1991" in provided references)

80. Garrett, G. (1998). *Partisan politics in the global economy*. Cambridge University Press. [cite: 63]

81. Rudra, N. (2002). Globalization and the decline of the welfare state in less-developed countries. *International Organization*, *56*(2), 411–445. [cite: 151, 152]

82. Rodrik, D. (1998). Why do more open economies have bigger governments? *Journal of Political Economy*, *106*(5), 997–1032. [cite: 149]

83. Strange, S. (1996). *The retreat of the state: The diffusion of power in the world economy*. Cambridge University Press. [cite: 169]

84. Swank, D., & Betz, H.-G. (2003). Globalization, the welfare state and right-wing populism in western Europe. *Socio-Economic Review*, *1*(2), 215–245. [cite: 172, 173]

85. Swank, D., & Steinmo, S. (2002). The new political economy of taxation in advanced capitalist democracies. *American Journal of Political Science*, *46*(3), 642–655. [cite: 174, 175]

86. Iversen, T., & Wren, A. (1998). Equality, employment, and budgetary restraint: The trilemma of the service economy. *World Politics*, *50*(4), 507–546. [cite: 93]

87. Wren, A. (Ed.). (2013). *The political economy of the service transition*. Oxford University Press. [cite: 190]

88. Sachs, J. D., & Warner, A. M. (2001). The curse of natural resources. *European Economic Review*, *45*(4-6), 827–838. [cite: 155, 156]

89. Ahlquist, J. S., & Wibbels, E. (2010). Inequality and democratization: A contractarian approach. *Comparative Political Studies*, *43*(12), 1543–1574. [cite: 5, 6]

90. World Bank. (2020). *World Development Indicators*. https://data.worldbank.org/indicator. [cite: 190]

91. Coppedge, M., Gerring, J., Lindberg, S. I., Skaaning, S.-E., Teorell, J., Altman, D., Bernhard, M., Fish, M. S., Glynn, A., Hicken, A., Knutsen, C. H., Krusell, J., Lührmann, A., Marquardt, K. L., McMann, K., Mechkova, V., Olin, M., Paxton, P., Pemstein, D., & Stepanova, N. (2011). V-dem: A new way to measure democracy. *Journal of Democracy*, *25*(3), 159–169. [cite: 39, 40]

92. Wooldridge, J. M. (2010). *Econometric analysis of cross section and panel data*. MIT Press. [cite: 189]

93. Nannestad, P. (2008). What have we learned about generalized trust, if anything? *Annual Review of Political Science*, *11*, 413–436. [cite: 131]

94. Helliwell, J. F. (2002). *Well-being and social capital: Does suicide pose a puzzle?* National Bureau of Economic Research. [cite: 78]

95. Putnam, R. D. (2000). *Bowling alone: The collapse and revival of american community*. Simon & Schuster. [cite: 146]

96. Bouckaert, G. (2002). Trust and the management of international relations: The role of public administration and political leadership. *Administrative Science Quarterly*, *47*(4), 583–597. [cite: 23, 24]

97. Bjornskov, C. (2008). The determinants of trust. *Social Indicators Research*, *88*(2), 271–292. [cite: 16]

98. Huppert, F. A. (2005). The psychology of health and well-being. *Health and Quality of Life Outcomes*, *3*(1), 1–7. [cite: 86]

99. Delhey, J., & Newton, K. (2003). Does inequality breed social mistrust? *European Journal of Political Research*, *42*(4), 481–502. [cite: 45]

100. Luhmann, N. (1979). *Trust and power*. John Wiley & Sons. [cite: 121]

101. Kasara, K., & Suryanarayan, P. (2008). Urban bias in political decision making in developing countries. *Quarterly Journal of Political Science*, *3*(2), 193–208. [cite: 98]

102. Dalton, R. J. (2004). *Democratic challenges, democratic choices: The erosion of political support in advanced industrial democracies*. Oxford University Press. [cite: 42, 43]

103. Norris, P. (1999). Critical citizens: Global support for democratic governance. *Oxford University Press*. [cite: 134]

104. Rose, R. (2001). *Trust and democratic governance*. Russell Sage Foundation. [cite: 149, 150]

105. Putnam, R. D. (1995). Tuning in, tuning out: The strange disappearance of social capital in America. *PS: Political Science & Politics*, *28*(4), 664–683. [cite: 144, 145]

106. Paxton, P. (2002). Social capital and democracy: An interdependent relationship. *American Sociological Review*, *67*(2), 254–277. [cite: 137, 138]

107. Levi, M., & Stoker, L. (2000). Political trust and trustworthiness. *Annual Review of Political Science*, *3*(1), 475–507. [cite: 115, 116]

108. Dolan, P., Peasgood, T., & White, M. (2008). We don’t really know what makes us happy: A review of the economic literature on the factors associated with subjective well-being. *Journal of Economic Psychology*, *29*(1), 94–122. [cite: 47, 48]

109. European Social Survey. (2020). *ESS Round 1-10 Data*. https://www.europeansocialsurvey.org. [cite: 56]

110. Newton, K. (2001). Trust, social capital, civil society, and democracy. *International Political Science Review*, *22*(2), 201–214. [cite: 133]
